# Supplementary material for: Differential Oligomerization of Alpha versus Beta Amino Acids and Hydroxy Acids in Abiotic Proto-Peptide Synthesis Reactions
Source: Life (Basel). 2022 Feb 10;12(2):265. doi: 10.3390/life12020265 (PMC8876357; doi:10.3390/life12020265)
Supplement: Supplementary file 1 [file life-12-00265-s001.zip › life-1553370-supplementary.pdf]

Supporting Information

Differential Oligomerization of Alpha Versus Beta Amino Acids and Hydroxy Acids in Abiotic Proto-peptide Synthesis Reactions

Moran Frenkel-Pinter[a,b,c†], Kaitlin C Jacobson[a,b†], Jonathan Eskew-Martin[a,d], Jay G Forsythe[a,d], Martha A Grover[a,e], Loren Dean Williams[a,b*] and Nicholas V Hud[a,b*]

[a] NSF-NASA Center for Chemical Evolution, Georgia Institute of Technology, Atlanta, GA 30332 (USA)

[b] School of Chemistry & Biochemistry, Georgia Institute of Technology, Atlanta, GA 30332 (USA)

[c] Institute of Chemistry, The Hebrew University of Jerusalem, Israel 91904

[d] Department of Chemistry and Biochemistry, College of Charleston, Charleston, SC, 29424, USA

[e] School of Chemical & Biomolecular Engineering, Georgia Institute of Technology, Atlanta, GA 30332 (USA)

†Authors contributed equally

Table of Contents

Supplementary Methods 9

Synthesis of glcβ-Aba 9

Synthesis of lacβ-Aba 9

Synthesis of hpaβ-Aba 10

Table S1. Summary of MALDI mass spectrometry analysis of single-step dry-down reactions and dry/wet cycling reactions of alpha and beta hydroxy acids and amino acids. 11

Supplementary Figures 13

Figure S1. MS of a single-step dry-down reaction of glycolic acid (glc) and glycine (Gly) supports the formation of depsipeptides. 14

Figure S2. MS of a single-step dry-down reaction of glycolic acid (glc) and alanine (Ala) supports the formation of depsipeptides. 15

Figure S3. MS of a single-step dry-down reaction of glycolic acid (glc) and beta-alanine (β-Ala) supports the formation of depsipeptides. 16

Figure S4. MS of a single-step dry-down reaction of glycolic acid (glc) and beta-aminobutyric acid (β-Aba) supports the formation of depsipeptides. 17

Figure S5. MS of a single-step dry-down reaction of lactic acid (lac) and glycine (Gly) supports the formation of depsipeptides. 18

Figure S6. MS of a single-step dry-down reaction of lactic acid (lac) and alanine (Ala) supports the formation of depsipeptides. 19

Figure S7. MS of a single-step dry-down reaction of lactic acid (lac) and beta-alanine (β-Ala) supports the formation of depsipeptides. 20

Figure S8. MS of a single-step dry-down reaction of lactic acid (lac) and beta-aminobutyric acid (β-Aba) supports the formation of depsipeptides. 21

Figure S9. MS of a single-step dry-down reaction of 3-hydroxypropanoic acid (hpa) and glycine (Gly) supports the formation of depsipeptides. 23

Figure S10. MS of a single-step dry-down reaction of 3-hydroxypropanoic acid (hpa) and alanine (Ala) supports the formation of depsipeptides. 24

Figure S11. MS of a single-step dry-down reaction of 3-hydroxypropanoic acid (hpa) and beta-alanine (β-Ala) supports the formation of depsipeptides. 25

Figure S12. MS of a single-step dry-down reaction of 3-hydroxypropanoic acid (hpa) and beta-aminobutyric acid (β-Aba) supports the formation of depsipeptides. 26

Figure S13. MS of a single-step dry-down reaction of 3-hydroxybutanoic acid (hba) and glycine (Gly) supports the formation of depsipeptides. 27

Figure S14. MS of a single-step dry-down reaction of 3-hydroxybutanoic acid (hba) and alanine (Ala) supports the formation of depsipeptides. 28

Figure S15. MS of a single-step dry-down reaction of 3-hydroxybutanoic acid (hba) and beta-alanine (β-Ala) supports the formation of depsipeptides. 30

Figure S16. MS of a single-step dry-down reaction of 3-hydroxybutanoic acid (hba) and beta-aminobutyric acid (β-Aba) supports the formation of depsipeptides. 31

Figure S17. ESI-MS analysis of control single-step dry-down reactions of the hydroxy acids in the absence of amino acids. 32

Figure S18. Analysis of control single-step dry-down reactions of the amino acids in the absence of hydroxy acids. 33

Figure S19. ESI-MS analysis of stock solutions of β-amino acids before single-step dry-down reactions shows the existence of some peptide dimers are present prior to reaction. 34

Figure S20. FTIR spectra changes upon single-step dry-down reactions of glycolic acid (glc) and glycine (Gly) support the formation of depsipeptides. 35

Figure S21. FTIR spectra changes upon single-step dry-down reactions of glycolic acid (glc) and alanine (Ala) support the formation of depsipeptides. 36

Figure S22. FTIR spectra changes upon single-step dry-down reactions of glycolic acid (glc) and beta-alanine (β-Ala) support the formation of depsipeptides. 37

Figure S23. FTIR spectra changes upon single-step dry-down reactions of glycolic acid (glc) and beta-aminobutyric acid (β-Aba) support the formation of depsipeptides. 38

Figure S24. FTIR spectra changes upon single-step dry-down reactions of lactic acid (lac) and glycine (Gly) support the formation of depsipeptides. 39

Figure S25. FTIR spectra changes upon single-step dry-down reactions of lactic acid (lac) and alanine (Ala) support the formation of depsipeptides. 40

Figure S26. FTIR spectra changes upon single-step dry-down reactions of lactic acid (lac) and beta-alanine (β-Ala) support the formation of depsipeptides. 41

Figure S27. FTIR spectra changes upon single-step dry-down reactions of lactic acid (lac) and beta-aminobutryic acid (β-Aba) support the formation of depsipeptides.42

Figure S28. FTIR spectra changes upon single-step dry-down reactions of 3-hydroxypropanoic acid (hpa) and glycine (Gly) support the formation of depsipeptides. 43

Figure S29. FTIR spectra changes upon single-step dry-down reactions of 3-hydroxypropanoic acid (hpa) and alanine (Ala) support the formation of depsipeptides. 44

Figure 30. FTIR spectra changes upon single-step dry-down reactions of 3-hydroxypropanoic acid (hpa) and beta-alanine (β-Ala) support the formation of depsipeptides. 45

Figure S31. FTIR spectra changes upon single-step dry-down reactions of 3-hydroxypropanoic acid (hpa) and beta-aminobutyric acid (β-Aba) support the formation of depsipeptides. 46

Figure S32. FTIR spectra changes upon single-step dry-down reactions of 3-hydroxybutanoic acid (hba) and glycine (Gly) support the formation of depsipeptides. 47

Figure S33. FTIR spectra changes upon single-step dry-down reactions of 3-hydroxybutanoic acid (hba) and alanine (Ala) support the formation of depsipeptides. 48

Figure S34. FTIR spectra changes upon single-step dry-down reactions of 3-hydroxybutanoic acid (hba) and beta-alanine (β-Ala) support the formation of depsipeptides. 49

Figure S35. FTIR spectra changes upon single-step dry-down reactions of 3-hydroxybutanoic acid (hba) and beta-aminobutyric acid (β-Aba) support the formation of depsipeptides. 50

Figure S36. Circular Dichroism spectra of glycolic acid (glc) single-step dry-down reactions and dry/wet cycling reactions with the amino acids. 51

Figure S37. Circular Dichroism spectra of lactic acid (lac) single-step dry-down reactions and dry/wet cycling reactions with the amino acids. 52

Figure S38. Circular Dichroism spectra of 3-hydroxypropanoic acid (hpa) single-step dry-down reactions and dry/wet cycling reactions with the amino acids. 53

Figure S39. Circular Dichroism spectra of 3-hydroxybutanoic acid (hba) single-step dry-down reactions and dry/wet cycling reactions with the amino acids. 54

Figure S40. C18-HPLC analysis verifies the formation of depsipeptides following single-step dry-down reactions and dry/wet cycling reactions of glycolic acid (glc) and glycine (Gly). 55

Figure S41. C18-HPLC analysis verifies the formation of depsipeptides following single-step dry-down reactions and dry/wet cycling reactions of glycolic acid (glc) and alanine (Ala). 56

Figure S42. C18-HPLC analysis verifies the formation of depsipeptides following single-step dry-down reactions and dry/wet cycling reactions of glycolic acid (glc) and beta-alanine (β-Ala). 57

Figure S43. C18-HPLC analysis verifies the formation of depsipeptides following single-step dry-down reactions and dry/wet cycling reactions of glycolic acid (glc) and beta-aminobutyric acid (β-Aba). 59

Figure S44. C18-HPLC analysis verifies the formation of depsipeptides following single-step dry-down reactions and dry/wet cycling reactions of lactic acid (lac) and glycine (Gly). 60

Figure S45. C18-HPLC analysis verifies the formation of depsipeptides following single-step dry-down reactions and dry/wet cycling reactions of lactic acid (lac) and alanine (Ala). 61

Figure S46. C18-HPLC analysis verifies the formation of depsipeptides following single-step dry-down reactions and dry/wet cycling reactions of lactic acid (lac) and beta-alanine (β-Ala). 62

Figure S47. C18-HPLC analysis verifies the formation of depsipeptides following single-step dry-down reactions and dry/wet cycling reactions of lactic acid (lac) and beta-aminobutyric acid (β-Aba). 63

Figure S48. C18-HPLC analysis verifies the formation of depsipeptides following single-step dry-down reactions and dry/wet cycling reactions of 3-hydroxypropanoic acid (hpa) and glycine (Gly). 64

Figure S49. C18-HPLC analysis verifies the formation of depsipeptides following single-step dry-down reactions and dry/wet cycling reactions of 3-hydroxypropanoic acid (hpa) and alanine (Ala). 65

Figure S50. C18-HPLC analysis verifies the formation of depsipeptides following single-step dry-down reactions and dry/wet cycling reactions of 3-hydroxypropanoic acid (hpa) and beta-alanine (β-Ala). 67

Figure S51. C18-HPLC analysis verifies the formation of depsipeptides following single-step dry-down reactions and dry/wet cycling reactions of 3-hydroxypropanoic acid (hpa) and beta-aminobutyric acid (β-Aba). 68

Figure S52. C18-HPLC analysis verifies the formation of depsipeptides following single-step dry-down reactions and dry/wet cycling reactions of 3-hydroxybutanoic acid (hba) and glycine (Gly). 69

Figure S53. C18-HPLC analysis verifies the formation of depsipeptides following single-step dry-down reactions and dry/wet cycling reactions of 3-hydroxybutanoic acid (hba) and alanine (Ala). 70

Figure S54. C18-HPLC analysis verifies the formation of depsipeptides following single-step dry-down reactions and dry/wet cycling reactions of 3-hydroxybutanoic acid (hba) and beta-alanine (β-Ala). 71

Figure S55. C18-HPLC analysis verifies the formation of depsipeptides following single-step dry-down reactions and dry/wet cycling reactions of 3-hydroxybutanoic acid (hba) and beta-aminobutyric acid (β-Aba). 72

Figure S56. C18-HPLC analysis of single-step dry-down reactions of beta-aminobutyric acid (β-Aba) overlaid with authentic standards. 74

Figure S57. C18-HPLC analysis of control single-step dry-down and dry/wet cycling reactions of the hydroxy acids in the absence of amino acids. 75

Figure S58. C18-HPLC analysis of control single-step dry-down reactions of the amino acids in the absence of hydroxy acids. 76

Figure S59. ^{1}H NMR spectra exhibit the extent of depsipeptide formation upon (A) single-step dry-down reactions and (B) dry/wet cycling of glycolic acid (glc) and glycine (Gly). 77

Figure S60. ^{1}H NMR spectra exhibit the extent of depsipeptide formation upon (a) single-step dry-down reactions and (b) dry/wet cycling of glycolic acid (glc) and alanine (Ala). 78

Figure S61. ^{1}H NMR spectra exhibit the extent of depsipeptide formation upon (a) single-step dry-down reactions and (b) dry/wet cycling of glycolic acid (glc) and beta-alanine (β-Ala). 79

Figure S62. ^{1}H NMR spectra exhibit the extent of depsipeptide formation upon (a) single-step dry-down reactions and (b) dry/wet cycling of glycolic acid (glc) and beta-aminobutyric acid (β-Aba). 80

Figure S63. ^{1}H NMR spectra exhibit the extent of depsipeptide formation upon (a) single-step dry-down reactions and (b) dry/wet cycling of lactic acid (lac) and glycine (Gly). 82

Figure S64. ^{1}H NMR spectra exhibit the extent of depsipeptide formation upon (a) single-step dry-down reactions and (b) dry/wet cycling of lactic acid (lac) and alanine (Ala). 83

Figure S65. ^{1}H NMR spectra exhibit the extent of depsipeptide formation upon (a) single-step dry-down reactions and (b) dry/wet cycling of lactic acid (lac) and beta-alanine (β-Ala). 84

Figure S66. ^{1}H NMR spectra exhibit the extent of depsipeptide formation upon (a) single-step dry-down reactions and (b) dry/wet cycling of lactic acid (lac) and beta-aminobutyric acid (β-Aba). 85

Figure S67. ^1H NMR spectra exhibit the extent of depsipeptide formation upon (a) single-step dry-down reactions and (b) dry/wet cycling of 3-hydroxypropanoic acid (hpa) and glycine (Gly). 86

Figure S68. ^1H NMR spectra exhibit the extent of depsipeptide formation upon (a) single-step dry-down reactions and (b) dry/wet cycling of 3-hydroxypropanoic acid (hpa) and alanine (Ala). 87

Figure S69. ^1H NMR spectra exhibit the extent of depsipeptide formation upon (a) single-step dry-down reactions and (b) dry/wet cycling of 3-hydroxypropanoic acid (hpa) and beta-alanine (β-Ala). 89

Figure S70. ^1H NMR spectra exhibit the extent of depsipeptide formation upon (a) single-step dry-down reactions and (b) dry/wet cycling of 3-hydroxypropanoic acid (hpa) and beta-aminobutyric acid (β-Aba). 90

Figure S71. ^1H NMR spectra exhibit the extent of depsipeptide formation upon (a) single-step dry-down reactions and (b) dry/wet cycling of 3-hydroxybutanoic acid (hba) and glycine (Gly). 91

Figure S72. ^1H NMR spectra exhibit the extent of depsipeptide formation upon (a) single-step dry-down reactions and (b) dry/wet cycling of 3-hydroxybutanoic acid (hba) and alanine (Ala). 92

Figure S73. ^1H NMR spectra exhibit the extent of depsipeptide formation upon (a) single-step dry-down reactions and (b) dry/wet cycling of 3-hydroxybutanoic acid (hba) and beta-alanine (β-Ala). 93

Figure S74. ^1H NMR spectra exhibit the extent of depsipeptide formation upon (a) single-step dry-down reactions and (b) dry/wet cycling of 3-hydroxybutanoic acid (hba) and beta-aminobutyric acid (β-Aba). 94

Figure S75. ^1H NMR spectra of replicates of lactic acid (lac) and alanine (Ala) single-step dry-down reactions confirms reproducibility of extent of amino acid conversion. 96

Figure S76. ESI-MS of the last cycle in the extended dry/wet cycling reaction of lactic acid (lac) and alanine (Ala) supports the formation of depsipeptides. 97

Figure S77. ESI-MS of the last cycle in the extended dry/wet cycling reaction of lactic acid (lac) and β-aminobutyric acid (β-Aba) supports the formation of depsipeptides. 98

Figure S78. ESI-MS of the last cycle in the extended dry/wet cycling reaction of 3-hydroxybutanoic acid (hba) and alanine (Ala) supports the formation of depsipeptides. 99

Figure S79. ESI-MS of the last cycle in the extended dry/wet cycling reaction of 3-hydroxybutanoic acid (hba) and β-aminobutyric acid (β-Aba) supports the formation of depsipeptides. 100

Figure S80. C18-HPLC analysis following weekly dry/wet cycling of lactic acid (lac) and Alanine (Ala) verifies the formation of depsipeptides. 101

Figure S81. C18-HPLC analysis following weekly dry/wet cycling of lactic acid (lac) and β-aminobutyric acid (β-Aba) verifies the formation of depsipeptides. 102

Figure S82. C18-HPLC analysis following weekly dry/wet cycling of 3-hydroxybutanoic acid (hba) and alanine (Ala) verifies the formation of depsipeptides. 103

Figure S83. C18-HPLC analysis following weekly dry/wet cycling of 3-hydroxybutanoic acid (hba) and β-aminobutyric acid (β-Aba) verifies the formation of depsipeptides. 104

Figure S84. ^{1}H NMR spectra exhibit the extent of depsipeptide formation upon the final cycle of the extended dry/wet cycling of lactic acid (lac) and alanine (Ala). .. 106

Figure S85. ^{1}H NMR spectra exhibit the extent of depsipeptide formation upon the final cycle of the extended dry/wet cycling of lactic acid (lac) and β-aminobutyric acid (β-Aba). 107

Figure S86. ^{1}H NMR spectra exhibit the extent of depsipeptide formation upon the final cycle of the extended dry/wet cycling of hydroxybutanoic acid (hba) and alanine (Ala). 108

Figure S87. ^{1}H NMR spectra exhibit the extent of depsipeptide formation upon the final cycle of the extended dry/wet cycling of hydroxybutanoic acid (hba) and β-aminobutyric acid (β-Aba). 109

Supplementary Methods

Synthesis of glcβ-Aba

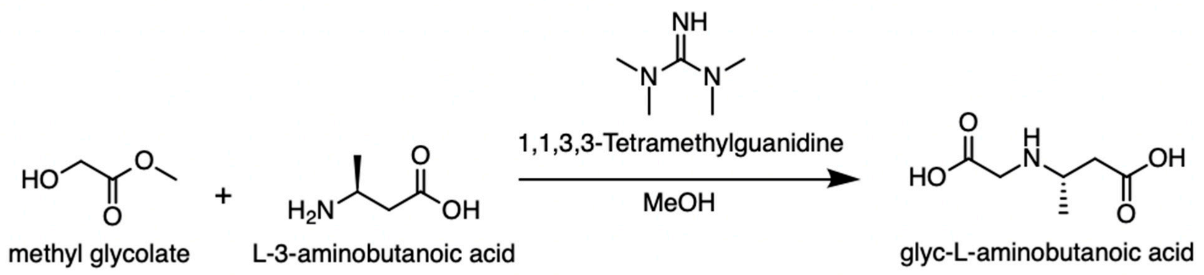

Methyl glycolate (300 µL, 3.88 mmol) and L-3-Aminobutanoic acid (400 mg, 3.88 mmol) were dissolved in a 1,1,3,3-Tetramethylguanidine (975 µL) and Methanol (777 µL) and reacted at 180 °C for 12 hours. Product was purified using preparative HPLC using a C18 column.

^{1}H NMR (500 MHz, D2O): δ = 4.325 (m, 1H), 4.04 (s, 2H), 2.59 (br d, 2H), 1.23 (d, 3H).

Synthesis of lacβ-Aba

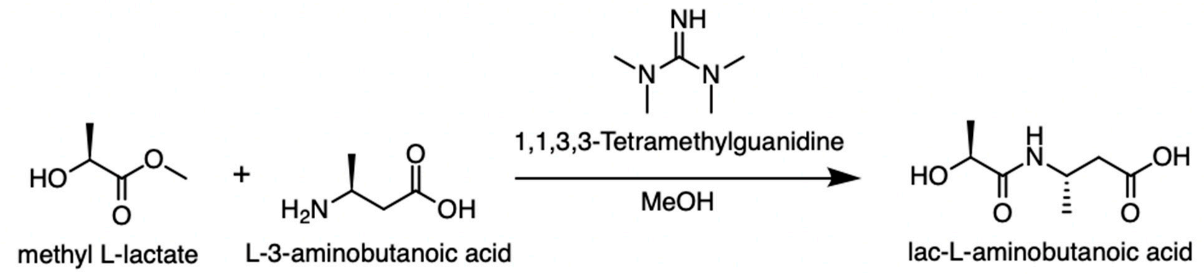

Methyl L-lactate (300 µL, 3.15 mmol) and L-3-Aminobutanoic acid (325 mg, 3.15 mmol) were dissolved in a 1,1,3,3-Tetramethylguanidine (790 µL) and Methanol (630 µL) and reacted at 180°C for 12 hours. Product was purified using preparative HPLC using a C18 column.

^{1}H NMR (500 MHz, D2O): δ = 4.28 (m, 1H), 4.21 (q, 1H), 2.58 (br d, 2H), 1.34 (d, 3H), 1.22 (d, 3H).

Synthesis of hpaβ-Aba

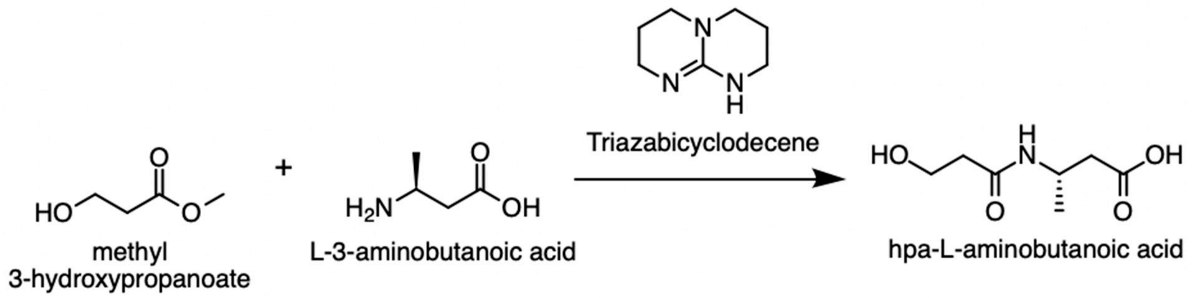

Methyl 3-hydroxypropanoate (400 µL, 4.22 mmol) and L-3-Aminobutanoic acid (552 mg, 5.35 mmol) were mixed with Triazabicyclodecene (1193 mg) and reacted at 135°C for 8 hours. Product was purified using preparative HPLC using a C18 column.

^{1}H NMR (500 MHz, D2O): δ = 4.265 (m, 1H), 3.81 (t, 2H), 2.56 (br d, 2H), 2.44 (t, 2H), 1.21 (d, 3H). High resolution.

Table S1. Summary of MALDI mass spectrometry analysis of single-step dry-down reactions and dry/wet cycling reactions of alpha and beta hydroxy acids and amino acids.

**Sample Name**	**Preparation Method**	**Maximal Oligomer Length**	**Dominant Species**
glycolic acid (**glc**) and alanine (**Ala**)	dry	25	**glc**
glycolic acid (**glc**) and alanine (**Ala**)	dry/wet	12	even mix
glycolic acid (**glc**) and glycine (**Gly**)	dry	23	**glc**
glycolic acid (**glc**) and glycine (**Gly**)	dry/wet	15	**glc**
glycolic acid (**glc**) and β-alanine (**β-Ala**)	dry	19	**β-Ala**
glycolic acid (**glc**) and β-alanine (**β-Ala**)	dry/wet	12	**β-Ala**
glycolic acid (**glc**) and β-aminobutyric acid (**β-Aba**)	dry	8	even mix
glycolic acid (**glc**) and β-aminobutyric acid (**β-Aba**)	dry/wet	8	**β-Aba**
3-hydroxybutanoic acid (**hba**) and alanine (**Ala**)	dry	14	**hba**
3-hydroxybutanoic acid (**hba**) and alanine (**Ala**)	dry/wet	12	**hba**
3-hydroxybutanoic acid (**hba**) and β-alanine (**β-Ala**)	dry	10	**β-Ala**
3-hydroxybutanoic acid (**hba**) and β-alanine (**β-Ala**)	dry/wet	9	**β-Ala**
3-hydroxybutanoic acid (**hba**) and β-aminobutyric acid (**β-Aba**)	dry	8	**hba**
3-hydroxybutanoic acid (**hba**) and β-aminobutyric acid (**β-Aba**)	dry/wet	5	**β-Aba**
3-hydroxybutanoic acid (**hba**) and glycine (**Gly**)	dry	16	**hba**
3-hydroxybutanoic acid (**hba**) and glycine (**Gly**)	dry/wet	9	**hba**

3-hydroxypropionic acid (**hpa**) and alanine (**Ala**)	dry	19	**hpa**
3-hydroxypropionic acid (**hpa**) and alanine (**Ala**)	dry/wet	14	**hpa**
3-hydroxypropionic acid (**hpa**) and β-alanine (**β-Ala**)	dry	14	**β-Ala**
3-hydroxypropionic acid (**hpa**) and β-alanine (**β-Ala**)	dry/wet	14	**β-Ala**
3-hydroxypropionic acid (**hpa**) and β-aminobutyric acid (**β-Aba**)	dry	15	**hpa**
3-hydroxypropionic acid (**hpa**) and β-aminobutyric acid (**β-Aba**)	dry/wet	11	**hpa**
3-hydroxypropionic acid (**hpa**) and glycine (**Gly**)	dry	19	**hpa**
3-hydroxypropionic acid (**hpa**) and glycine (**Gly**)	dry/wet	17	**hpa**
lactic acid (**lac**) and β-aminobutyric acid (**β-Aba**)	dry	5	**β-Aba**
lactic acid (**lac**) and β-aminobutyric acid (**β-Aba**)	dry/wet	0	no appreciable polymerization
lactic acid (**lac**) and alanine (**Ala**)	dry	25	**lac**
lactic acid (**lac**) and alanine (**Ala**)	dry/wet	14	**lac**
lactic acid (**lac**) and β-alanine (**β-Ala**)	dry	13	**β-Ala**
lactic acid (**lac**) and β-alanine (**β-Ala**)	dry/wet	8	**β-Ala**
lactic acid (**lac**) and glycine (**Gly**)	dry	25	**lac**
lactic acid (**lac**) and glycine (**Gly**)	dry/wet	14	**Gly**

Supplementary Figures

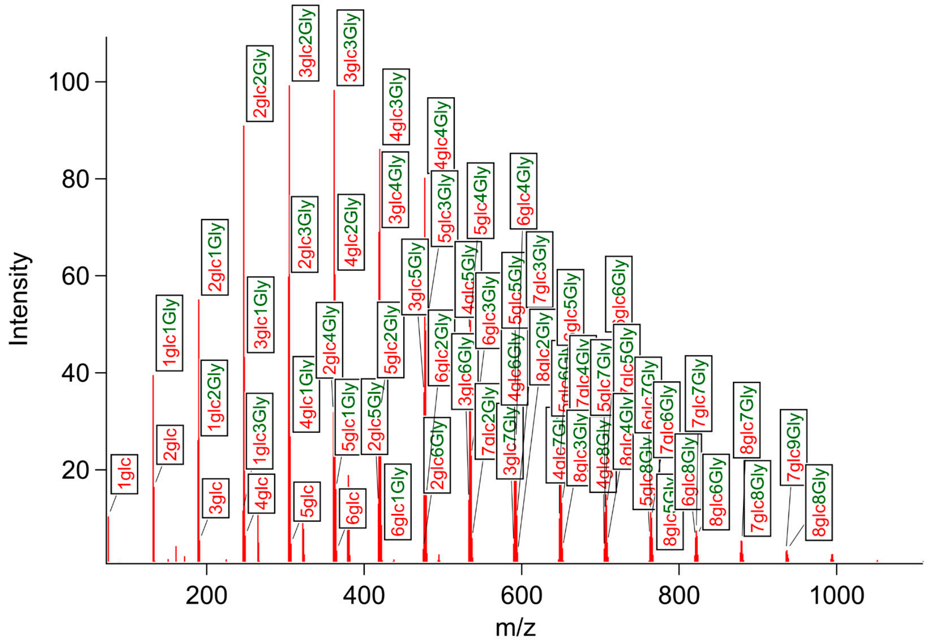

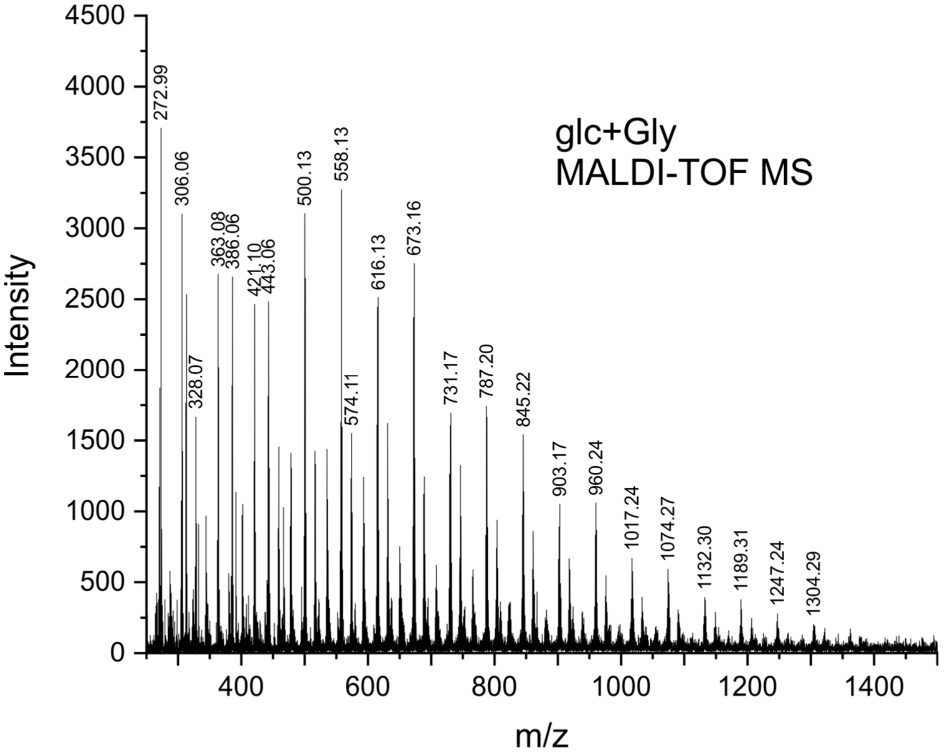

Figure S1. MS of a single-step dry-down reaction of glycolic acid (glc) and glycine (Gly) supports the formation of depsipeptides. glc and **Gly** were dried at 85 ˚C for seven days and the resulting depsipeptides were analyzed by negative-mode ESI-MS and MALDI-TOF MS, indicating a variety of depsipeptides. **glc** is labeled in red, **Gly** is labeled in green. All labeled species correspond to [M-H]$^-$ ions.

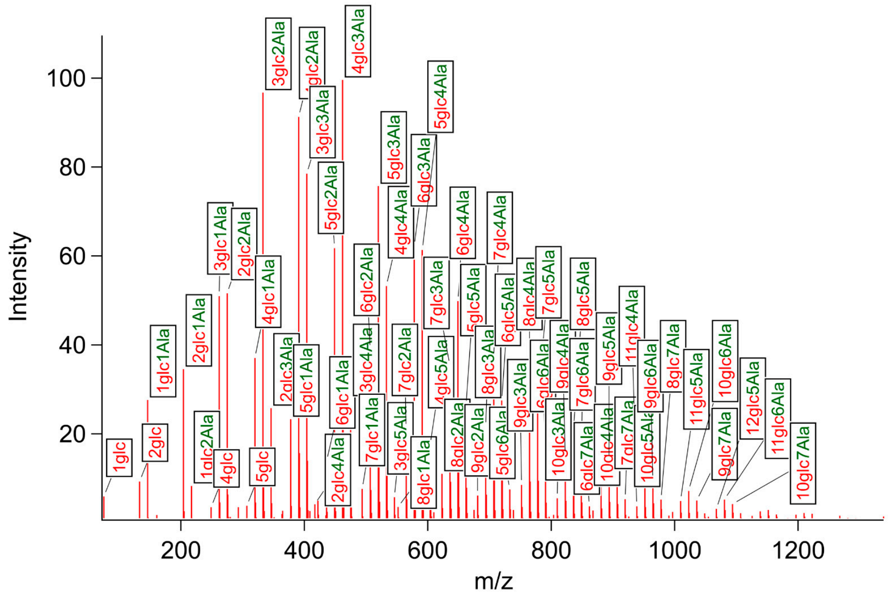

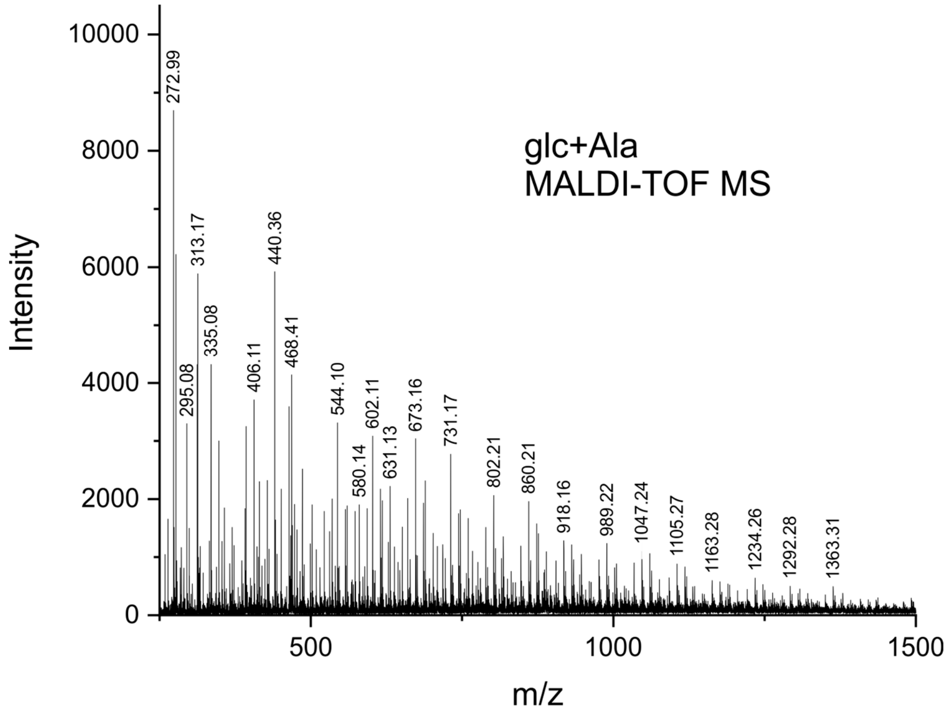

Figure S2. MS of a single-step dry-down reaction of glycolic acid (glc) and alanine (Ala) supports the formation of depsipeptides. glc and **Ala** were dried at 85 ˚C for seven days and the resulting depsipeptides were analyzed by negative-mode ESI-MS and MALDI-TOF MS, indicating a variety of depsipeptides. **glc** is labeled in red, **Ala** is labeled in green. All labeled species correspond to $[M-H]^-$ ions.

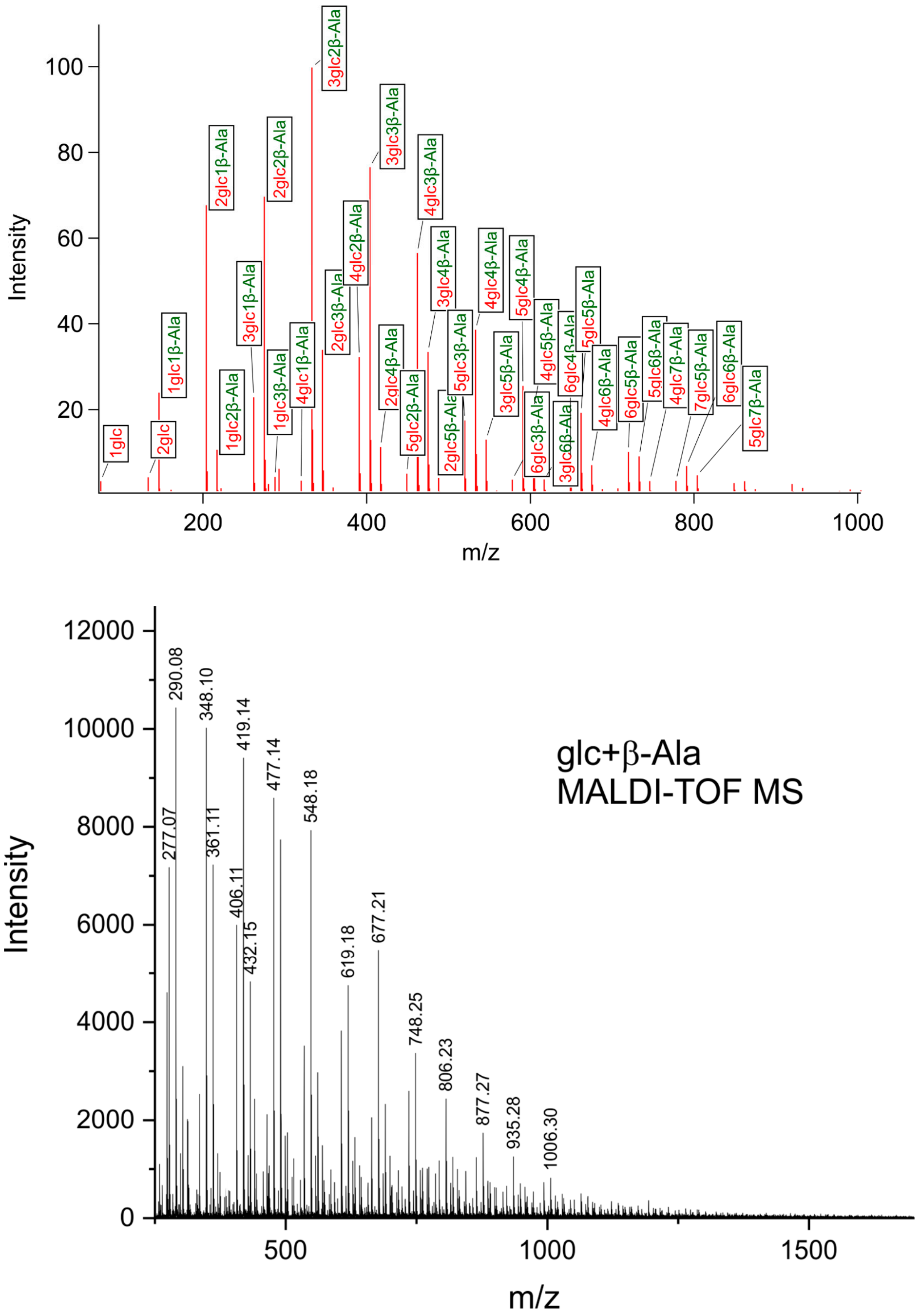

Figure S3. MS of a single-step dry-down reaction of glycolic acid (glc) and beta-alanine (β-Ala) supports the formation of depsipeptides. glc and **β-Ala** were dried at 85 ˚C for seven days and the resulting depsipeptides were analyzed by negative-mode ESI-MS and MALDI-TOF MS, indicating a variety of depsipeptides. **glc** is labeled in red; **β-Ala** is labeled in green. All labeled species correspond to [M-H]$^-$ ions.

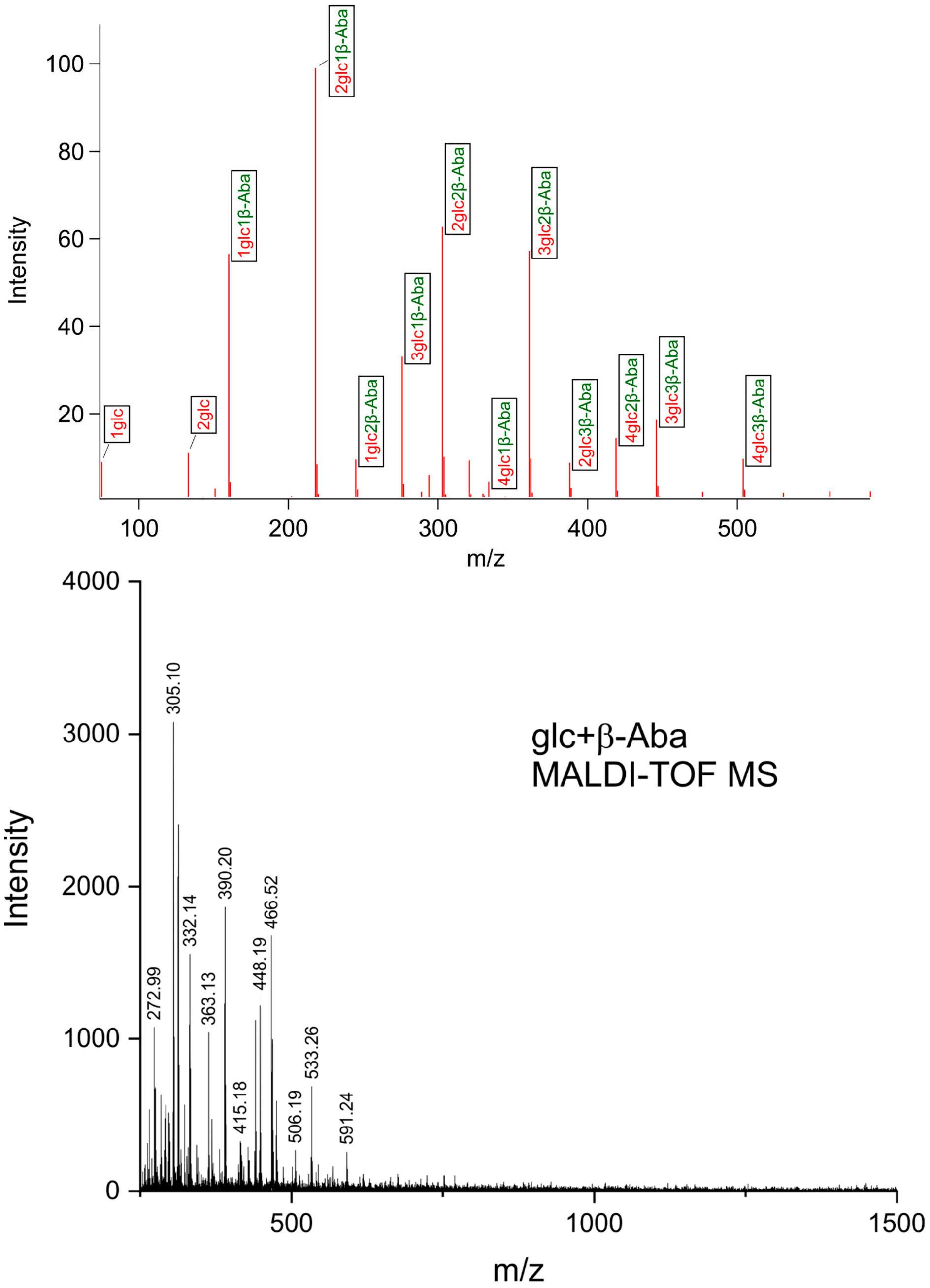

Figure S4. MS of a single-step dry-down reaction of glycolic acid (glc) and beta-aminobutyric acid (β-Aba) supports the formation of depsipeptides. glc and **β-Aba** were dried at 85 ˚C for seven days and the resulting depsipeptides were analyzed by negative-mode ESI-MS and MALDI-TOF MS, indicating a variety of depsipeptides. **glc** is labeled in red; **β-Aba** is labeled in green. All labeled species correspond to $[M\text{-}H]^-$ ions.

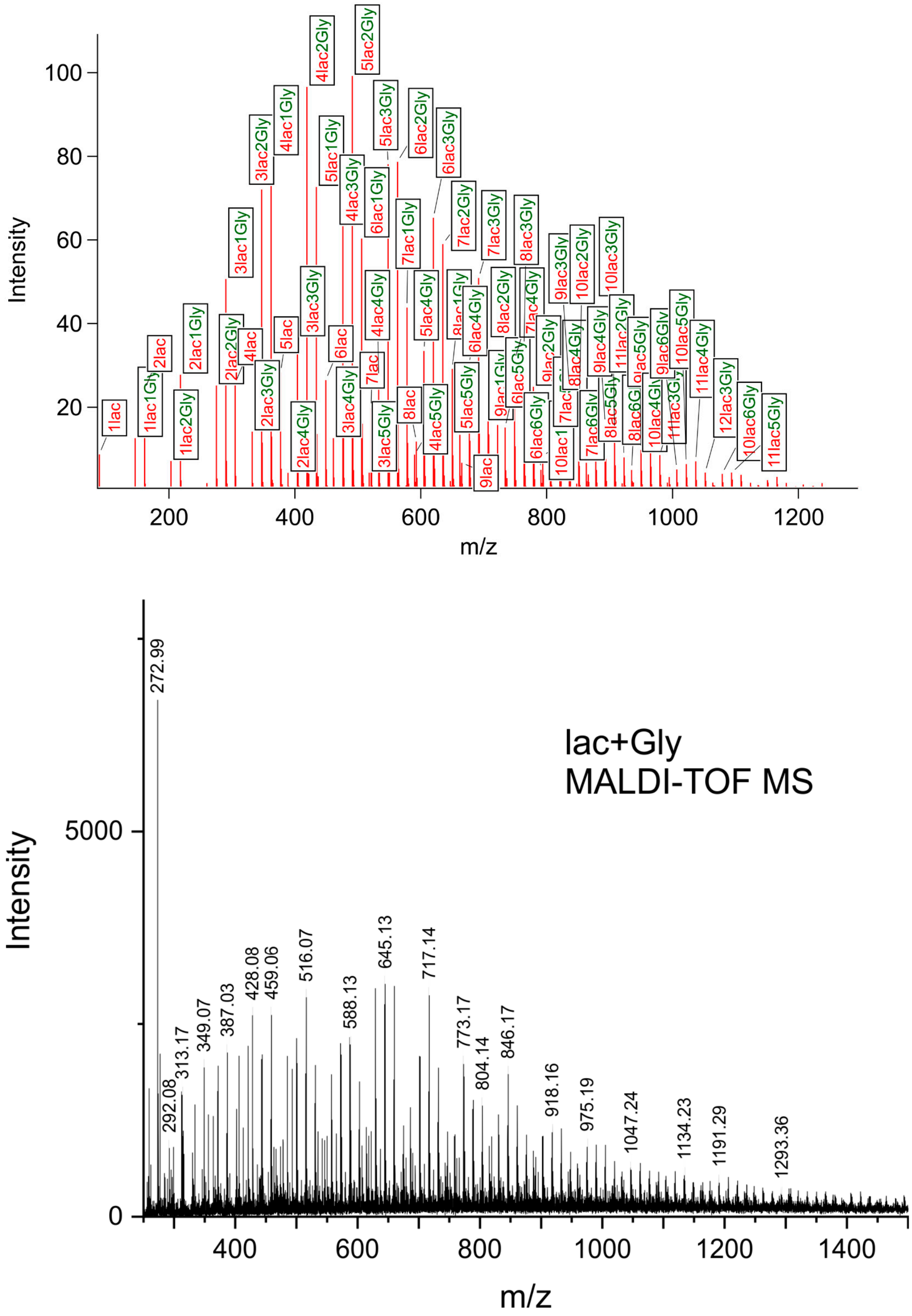

Figure S5. MS of a single-step dry-down reaction of lactic acid (lac) and glycine (Gly) supports the formation of depsipeptides. lac and **Gly** were dried at 85 ˚C for seven days and the resulting depsipeptides were analyzed by negative-mode ESI-MS and MALDI-TOF MS, indicating a variety of depsipeptides. **lac** is labeled in red; **Gly** is labeled in green. All labeled species correspond to $[M-H]^-$ ions.

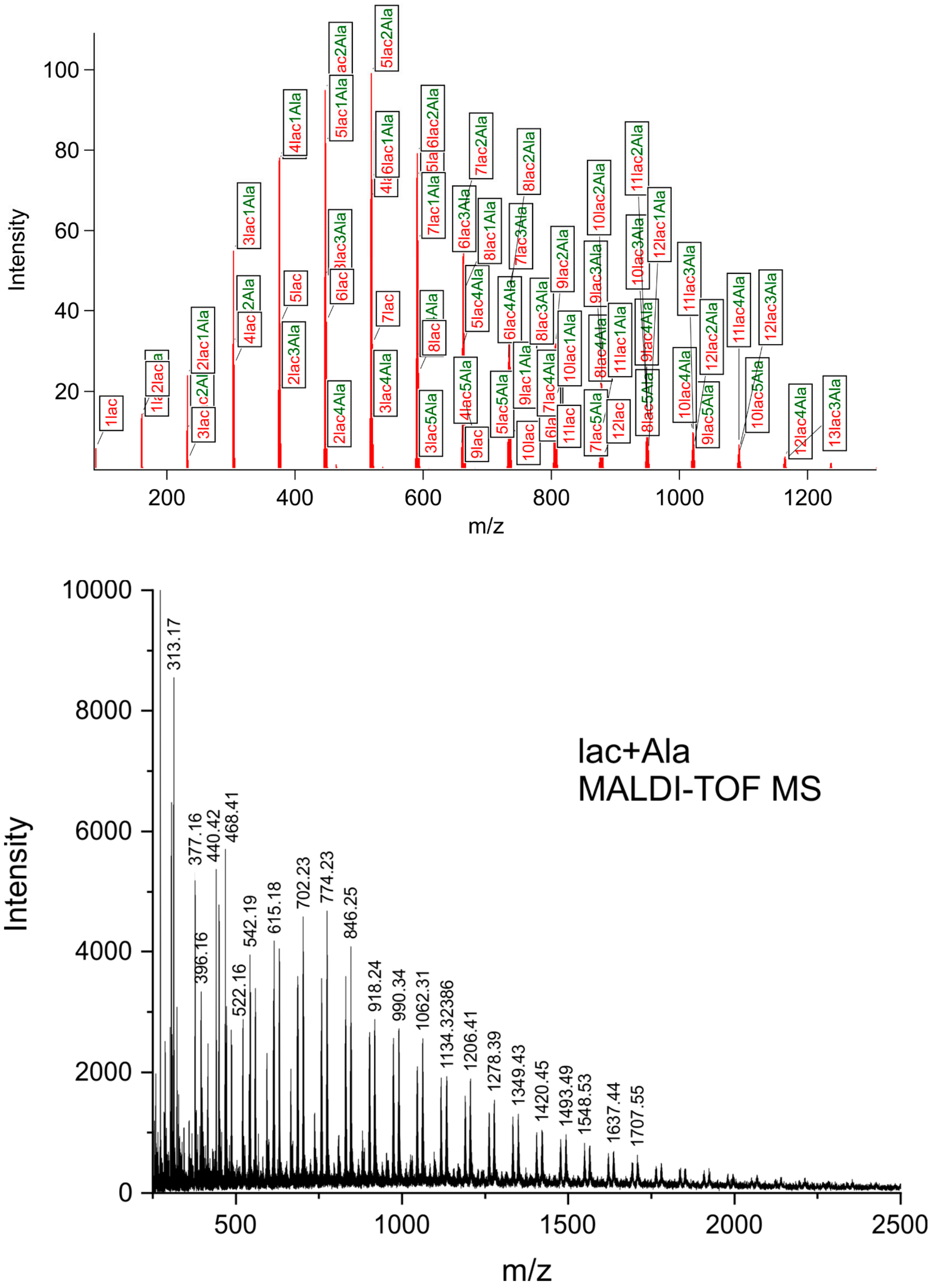

Figure S6. MS of a single-step dry-down reaction of lactic acid (lac) and alanine (Ala) supports the formation of depsipeptides. lac and **Ala** were dried at 85 ˚C for seven days and the resulting depsipeptides were analyzed by negative-mode ESI-MS and MALDI-TOF MS, indicating a variety of depsipeptides. **lac** is labeled in red; **Ala** is labeled in green. All labeled species correspond to [M-H]$^-$ ions.

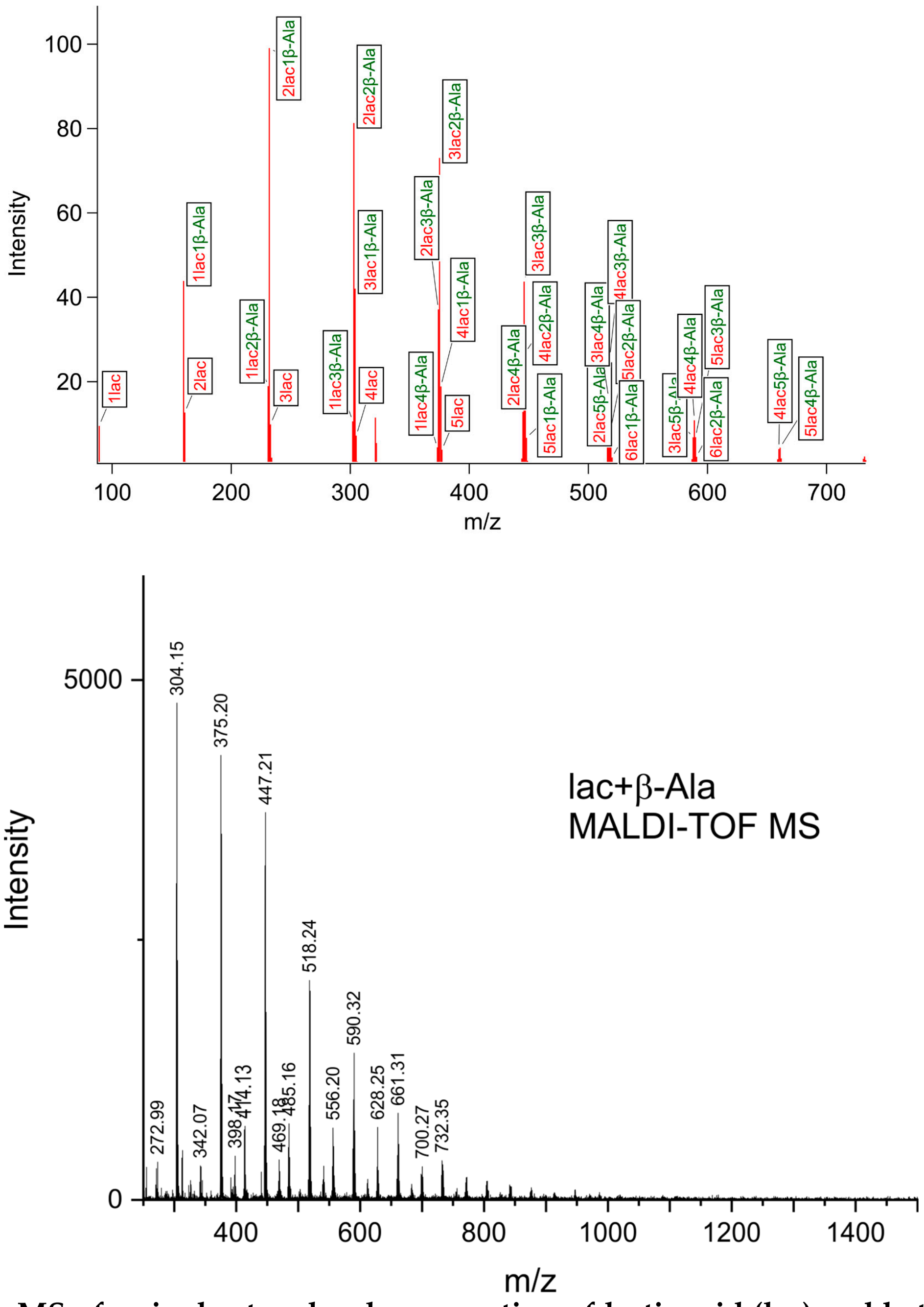

Figure S7. MS of a single-step dry-down reaction of lactic acid (lac) and beta-alanine (β-Ala) supports the formation of depsipeptides. lac and **β-Ala** were dried at 85 ˚C for seven days and the resulting depsipeptides were analyzed by negative-mode ESI-MS and

MALDI-TOF MS, indicating a variety of depsipeptides. **lac** is labeled in red; **β-Ala** is labeled in green. All labeled species correspond to [M-H]$^-$ ions.

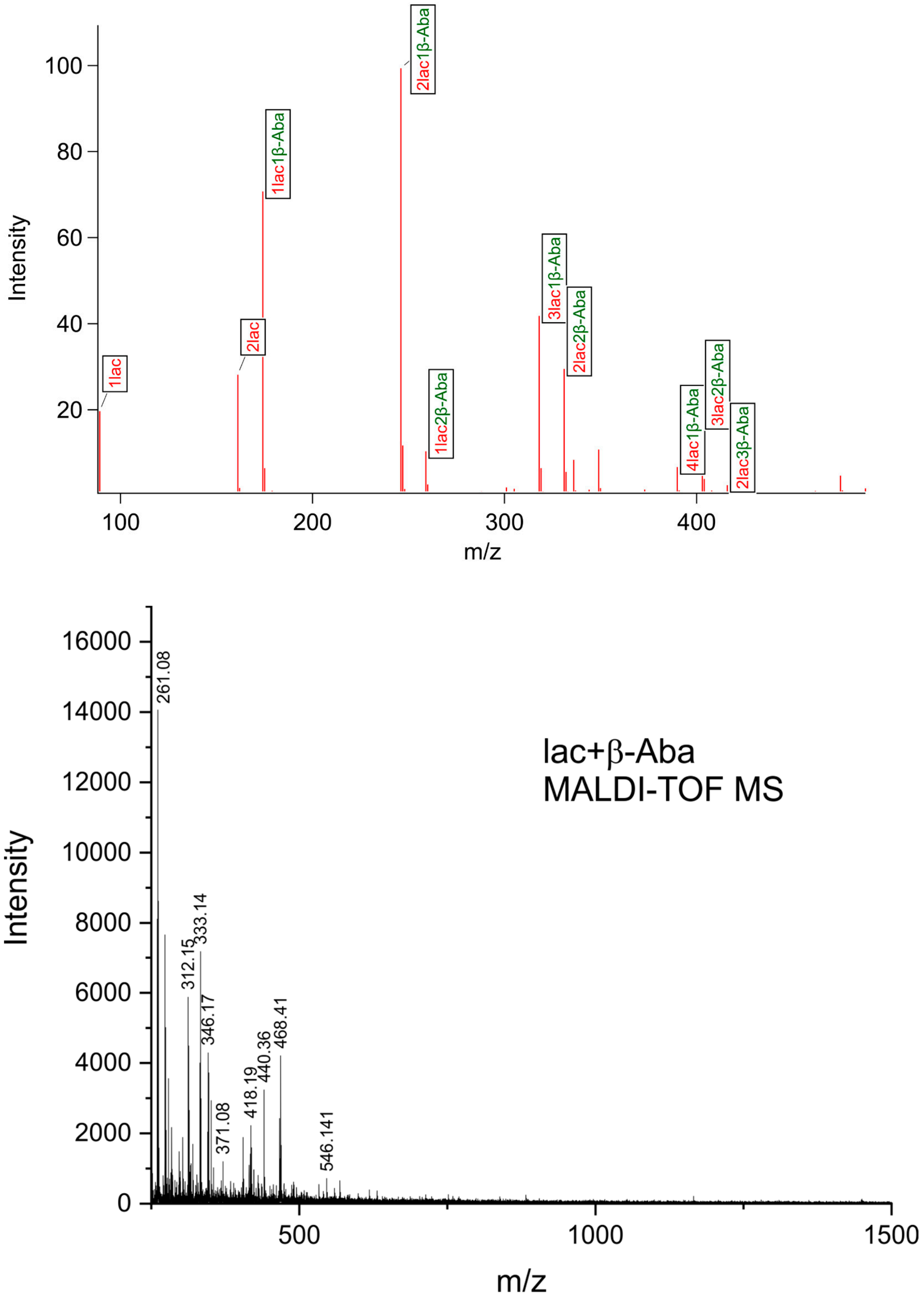

Figure S8. MS of a single-step dry-down reaction of lactic acid (lac) and beta-aminobutyric acid (β-Aba) supports the formation of depsipeptides. lac and **β-Aba**

were dried at 85 ˚C for seven days and the resulting depsipeptides were analyzed by negative-mode ESI-MS and MALDI-TOF MS, indicating a variety of depsipeptides. **lac** is labeled in red; **β-Aba** is labeled in green. All labeled species correspond to [M-H]$^{-}$ ions.

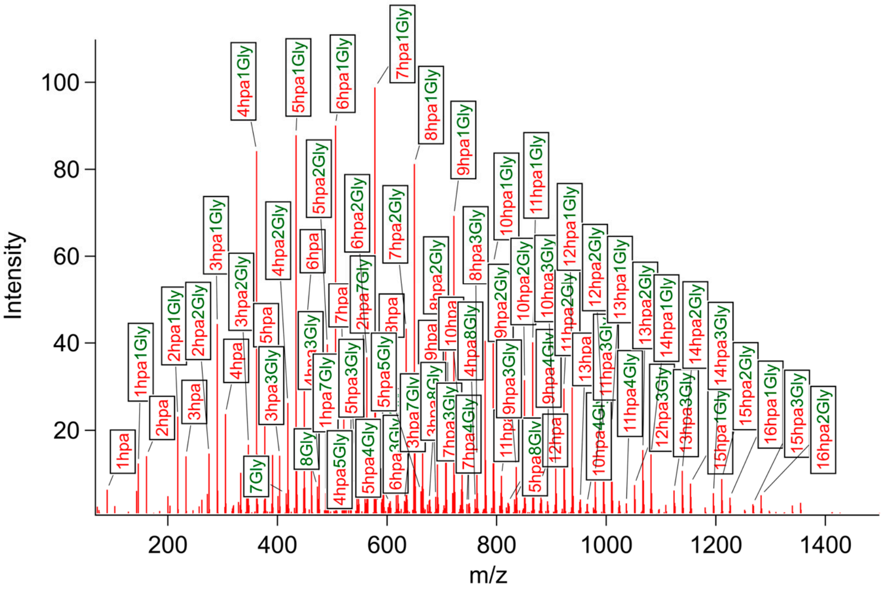

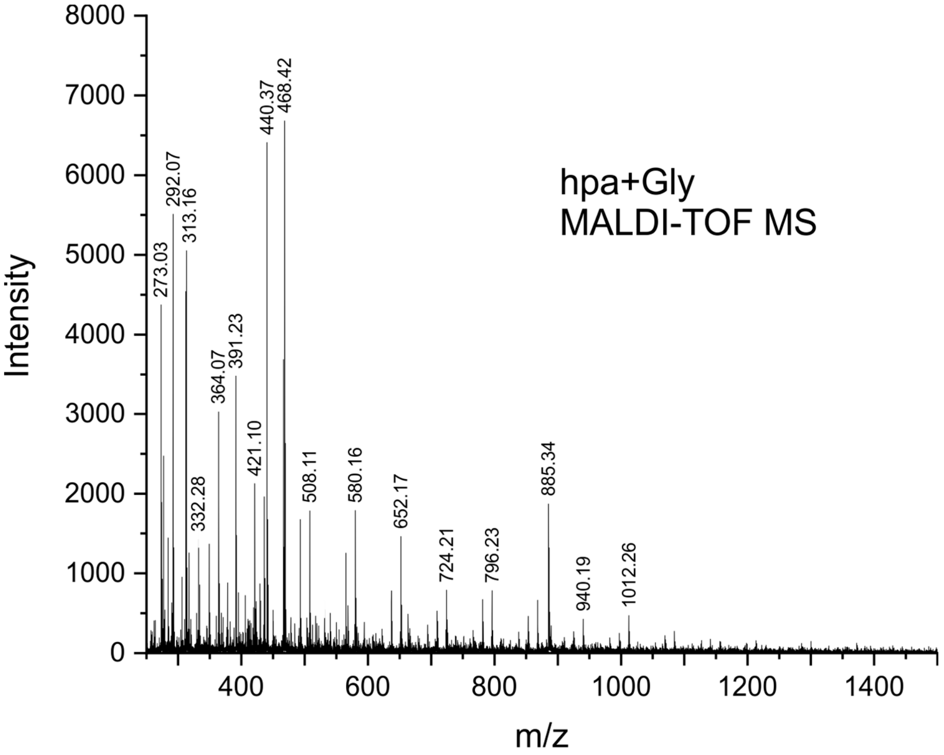

Figure S9. MS of a single-step dry-down reaction of 3-hydroxypropanoic acid (hpa) and glycine (Gly) supports the formation of depsipeptides. hpa and **Gly** were dried at 85 ˚C for seven days and the resulting depsipeptides were analyzed by negative-mode ESI-MS and MALDI-TOF MS, indicating a variety of depsipeptides. **hpa** is labeled in red; **Gly** is labeled in green. All labeled species correspond to [M-H]$^-$ ions.

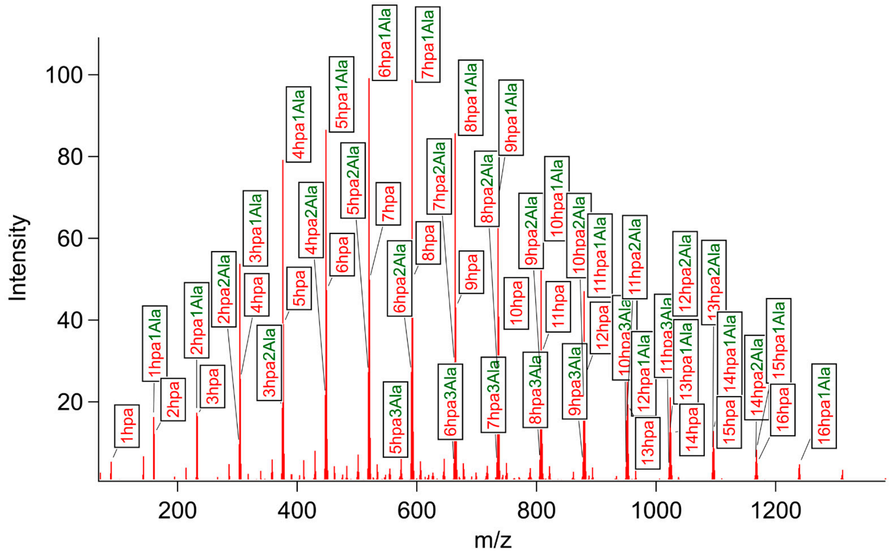

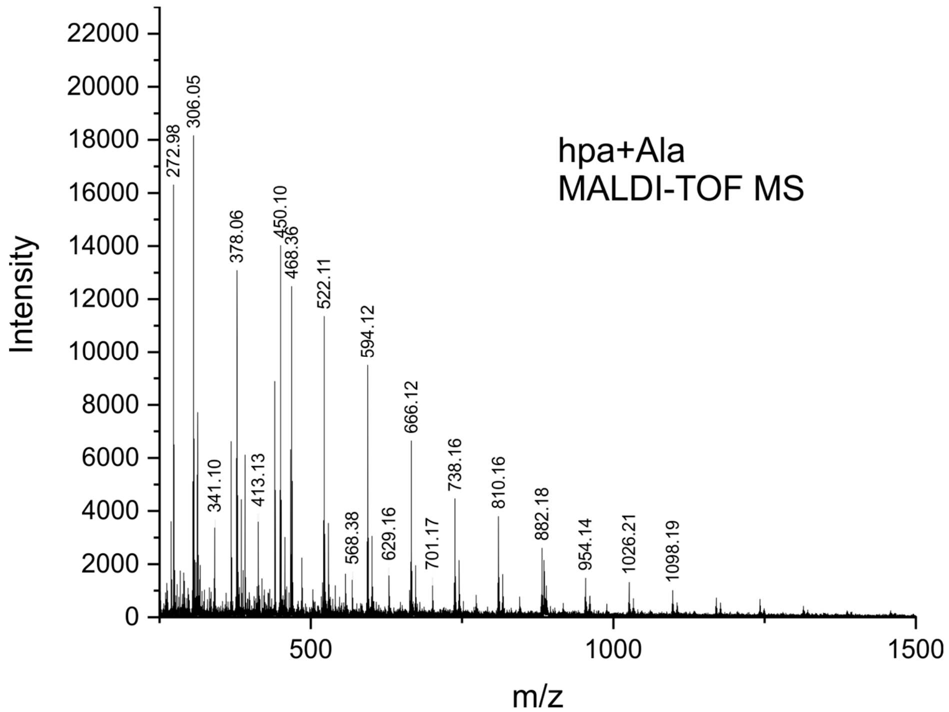

Figure S10. MS of a single-step dry-down reaction of 3-hydroxypropanoic acid (hpa) and alanine (Ala) supports the formation of depsipeptides. hpa and **Ala** were dried at 85 ˚C for seven days and the resulting depsipeptides were analyzed by negative-mode ESI-MS and MALDI-TOF MS, indicating a variety of depsipeptides. **hpa** is labeled in red; **Ala** is labeled in green. All labeled species correspond to [M-H]$^-$ ions.

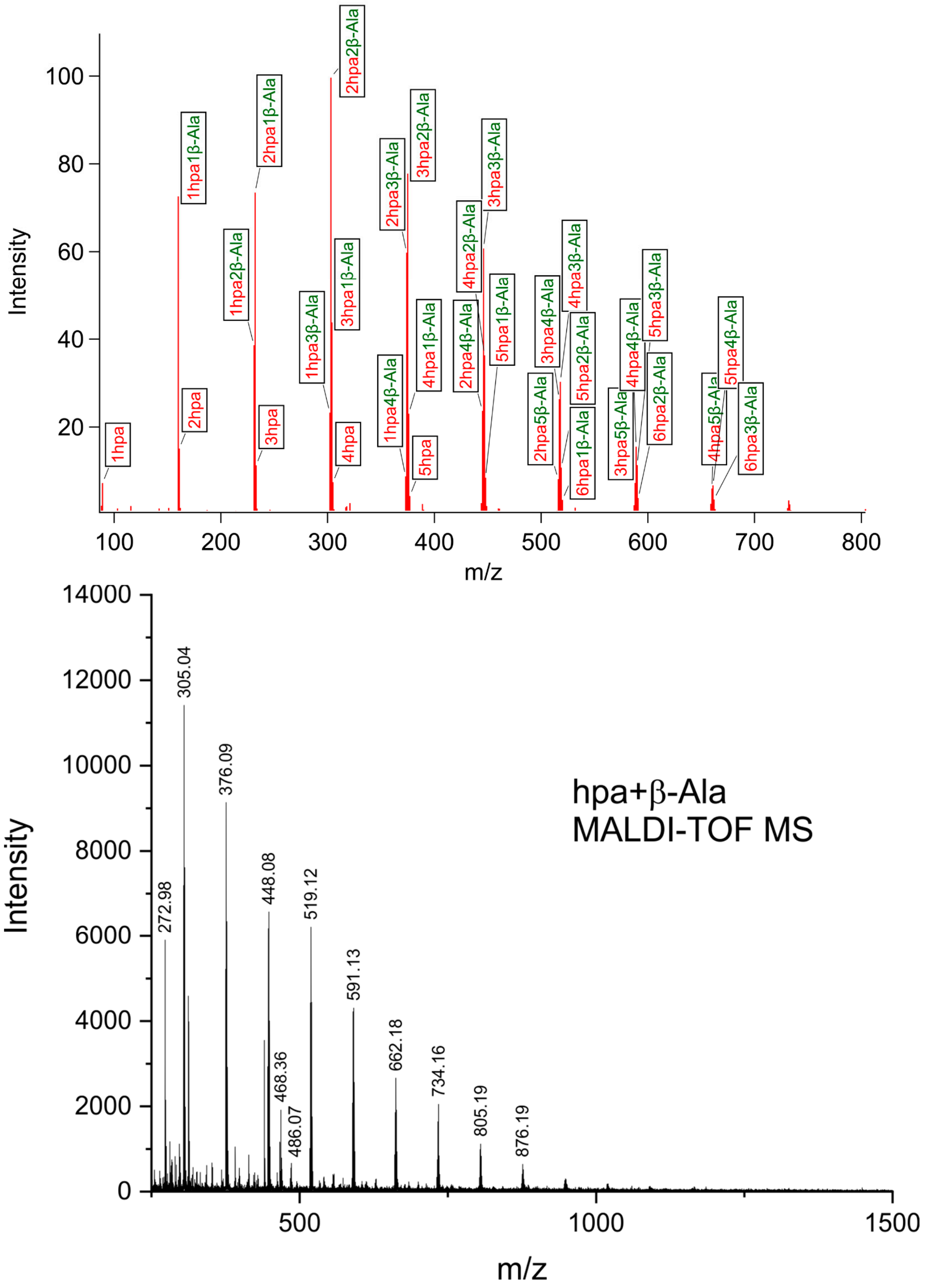

Figure S11. MS of a single-step dry-down reaction of 3-hydroxypropanoic acid (hpa) and beta-alanine (β-Ala) supports the formation of depsipeptides. hpa and **β-Ala** were dried at 85 °C for seven days and the resulting depsipeptides were analyzed by negative-mode ESI-MS and MALDI-TOF MS, indicating a variety of depsipeptides. **hpa** is labeled in red; **β-Ala** is labeled in green. All labeled species correspond to $[M-H]^-$ ions.

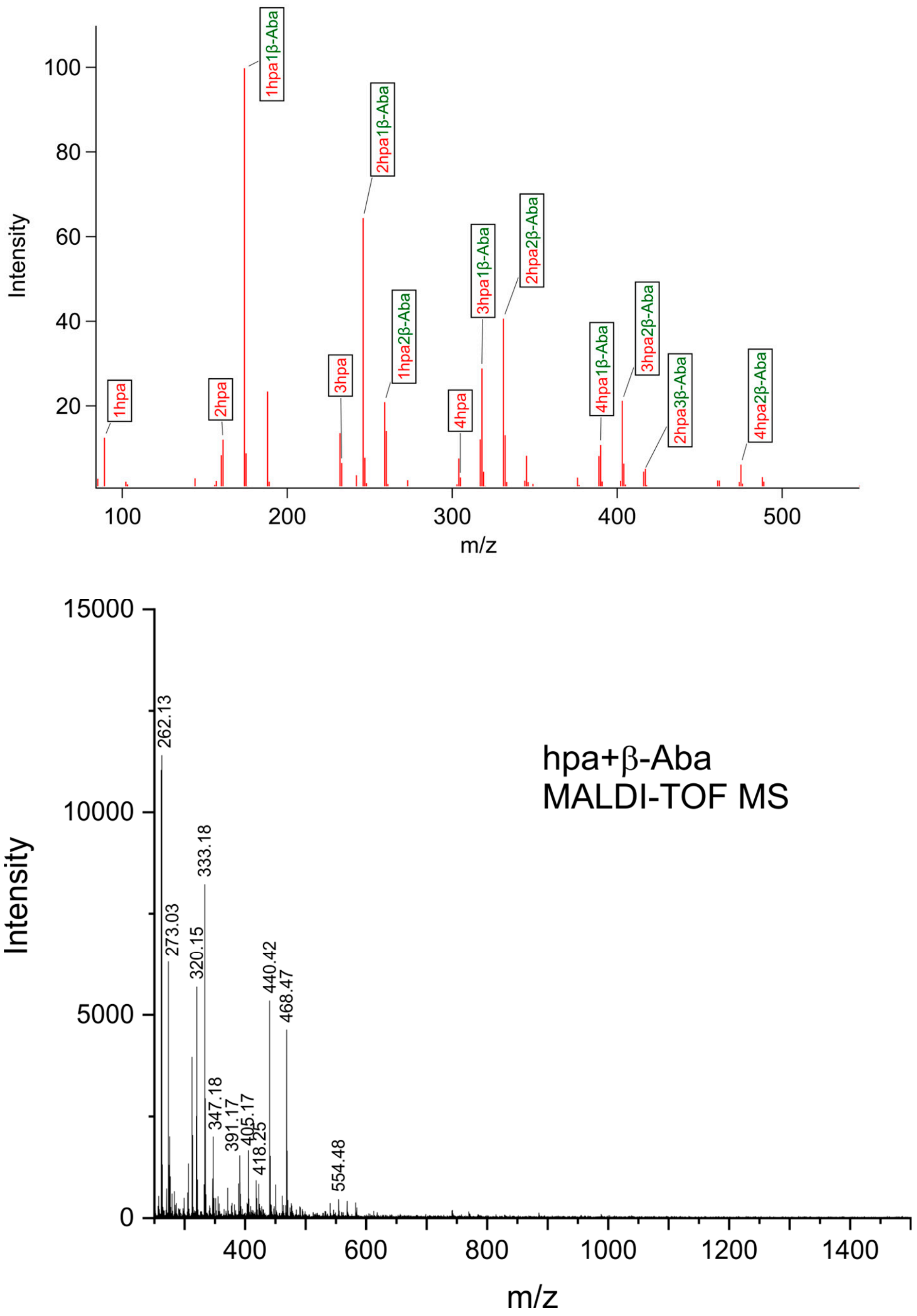

Figure S12. MS of a single-step dry-down reaction of 3-hydroxypropanoic acid (hpa) and beta-aminobutyric acid (β-Aba) supports the formation of depsipeptides. hpa and **β-Aba** were dried at 85 ˚C for seven days and the resulting depsipeptides were analyzed by negative-mode ESI-MS and MALDI-TOF MS, indicating a variety of depsipeptides. **hpa** is labeled in red; **β-Aba** is labeled in green. All labeled species correspond to $[M-H]^-$ ions.

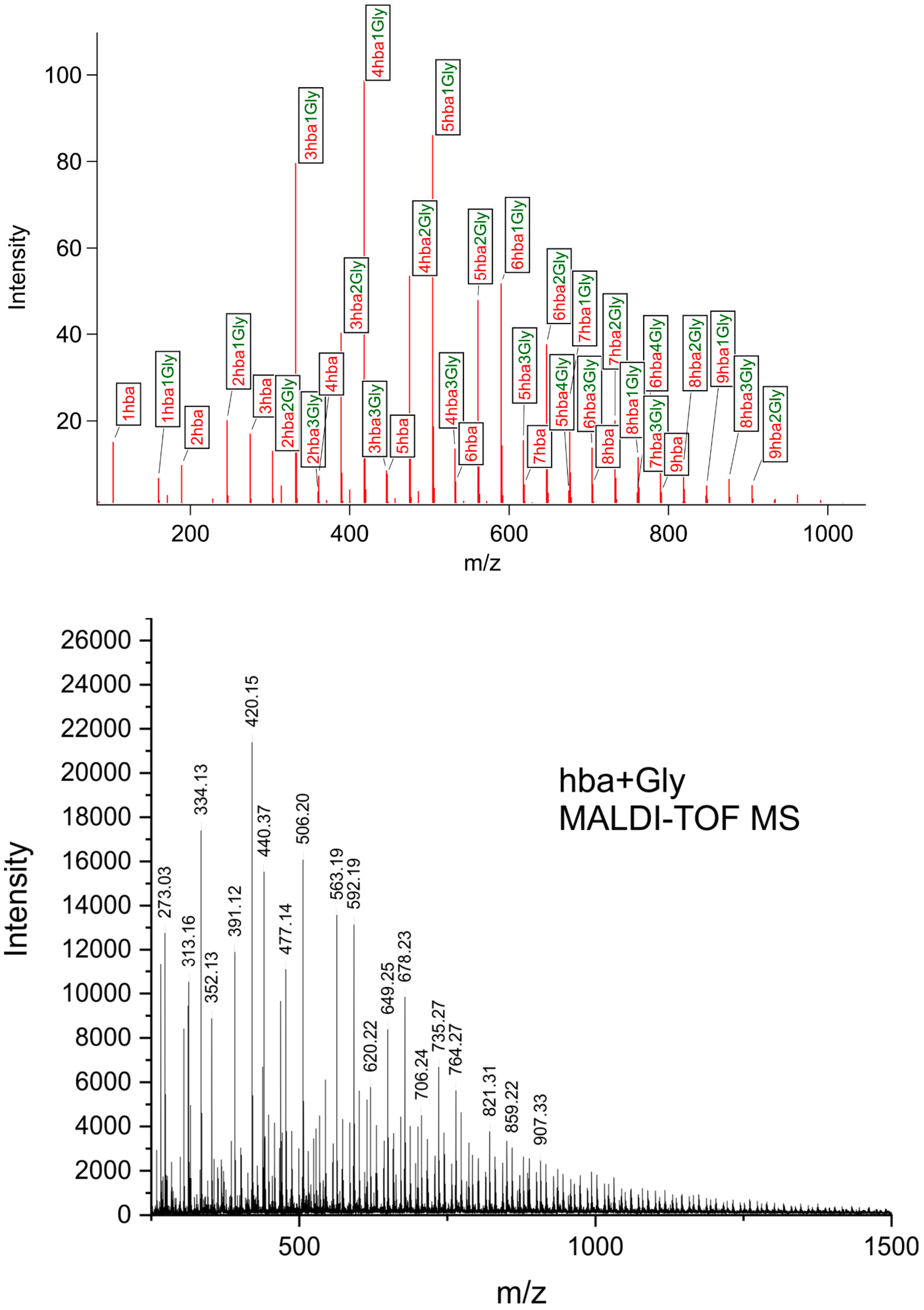

Figure S13. MS of a single-step dry-down reaction of 3-hydroxybutanoic acid (hba) and glycine (Gly) supports the formation of depsipeptides. hba and **Gly** were dried at 85 °C for seven days and the resulting depsipeptides were analyzed by negative-mode ESI-MS

and MALDI-TOF MS, indicating a variety of depsipeptides. **hba** is labeled in red; **Gly** is labeled in green. All labeled species correspond to [M-H]$^-$ ions.

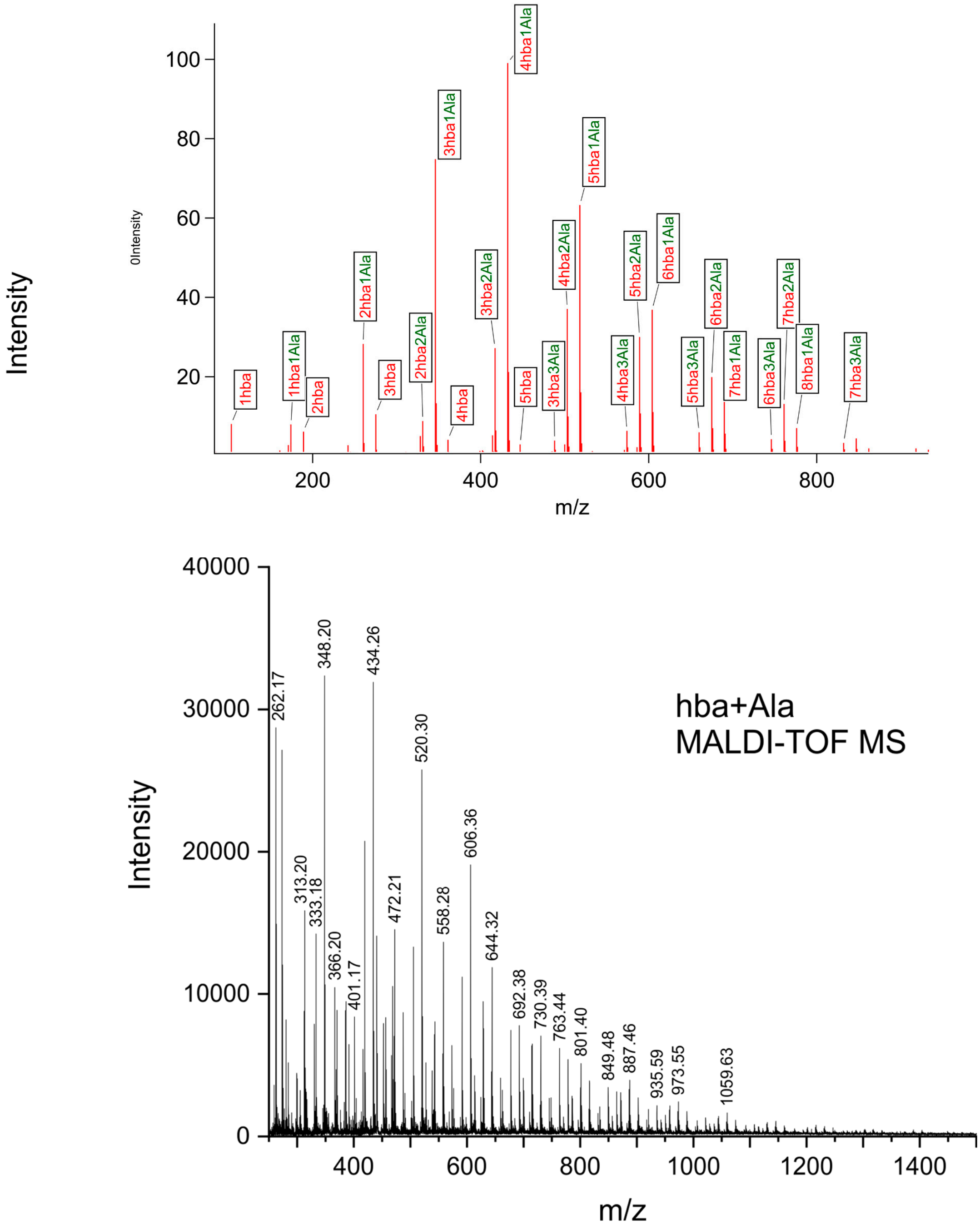

Figure S14. MS of a single-step dry-down reaction of 3-hydroxybutanoic acid (hba) and alanine (Ala) supports the formation of depsipeptides. **hba** and **Ala** were dried at 85 ˚C

for seven days and the resulting depsipeptides were analyzed by negative-mode ESI-MS and MALDI-TOF MS, indicating a variety of depsipeptides. **hba** is labeled in red; **Ala** is labeled in green. All labeled species correspond to [M-H]- ions.

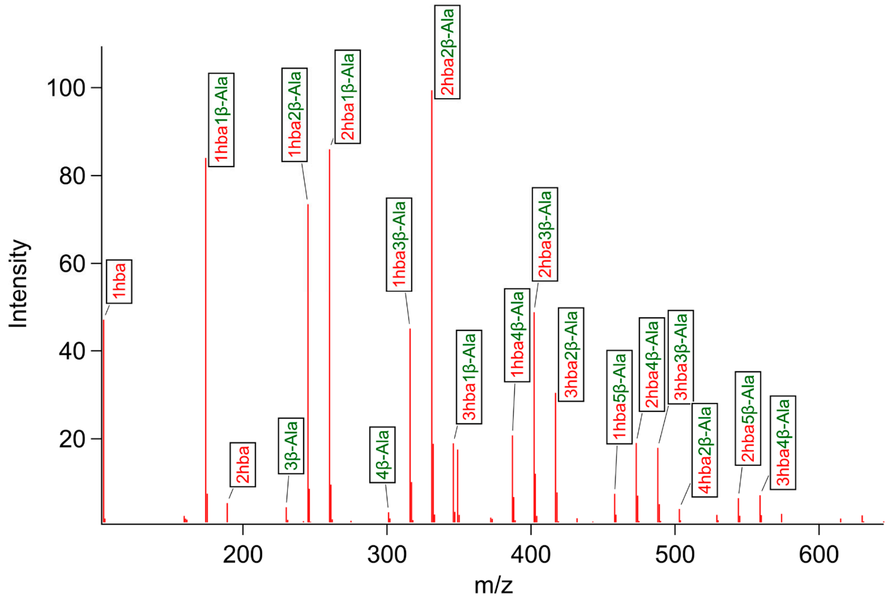

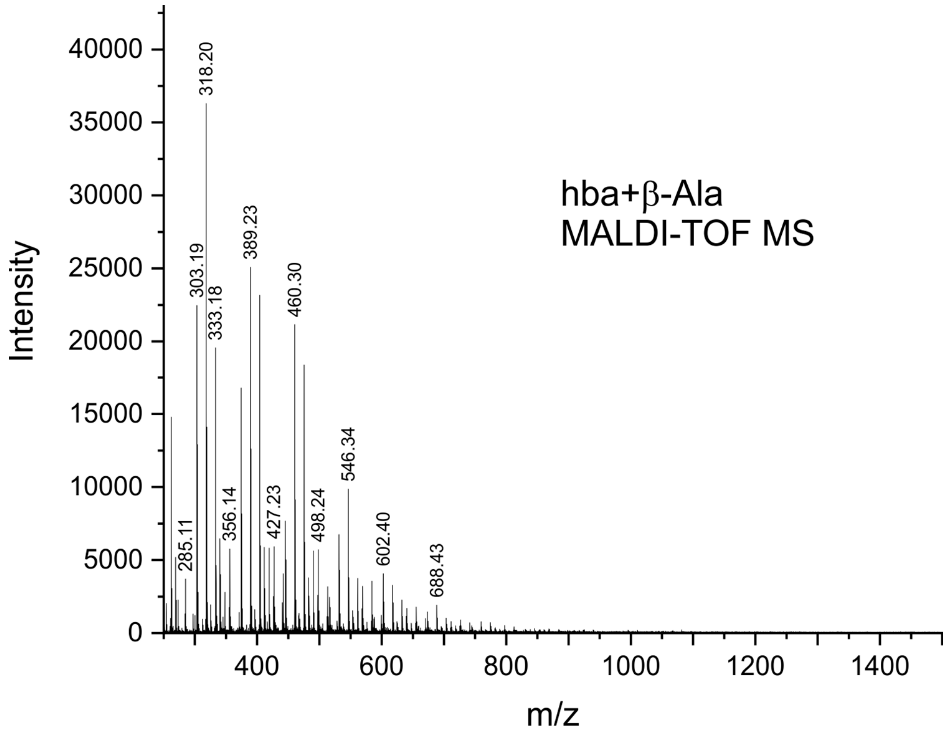

Figure S15. MS of a single-step dry-down reaction of 3-hydroxybutanoic acid (hba) and beta-alanine (β-Ala) supports the formation of depsipeptides. hba and **β-Ala** were dried at 85 ˚C for seven days and the resulting depsipeptides were analyzed by negative-mode ESI-MS and MALDI-TOF MS, indicating a variety of depsipeptides. **hba** is labeled in red; **β-Ala** is labeled in green. All labeled species correspond to $[M-H]^-$ ions.

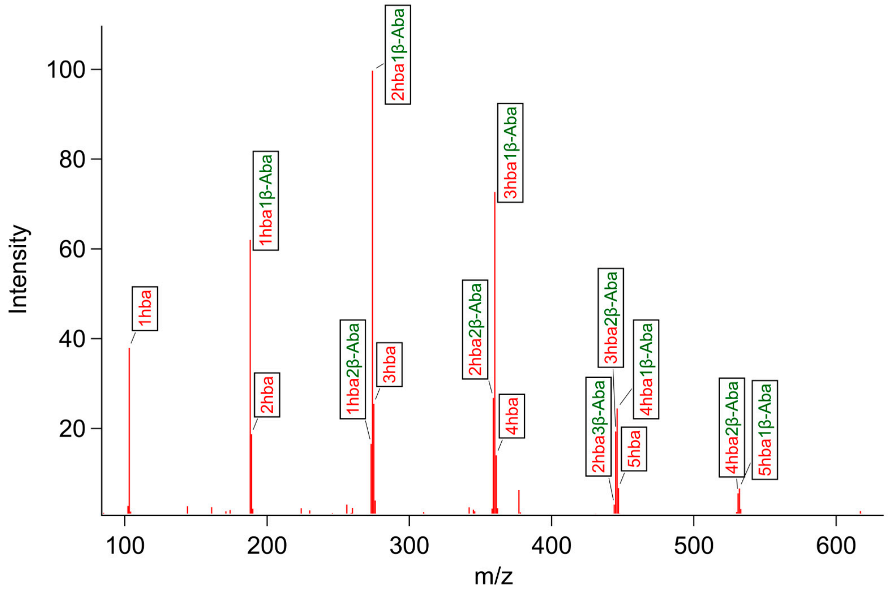

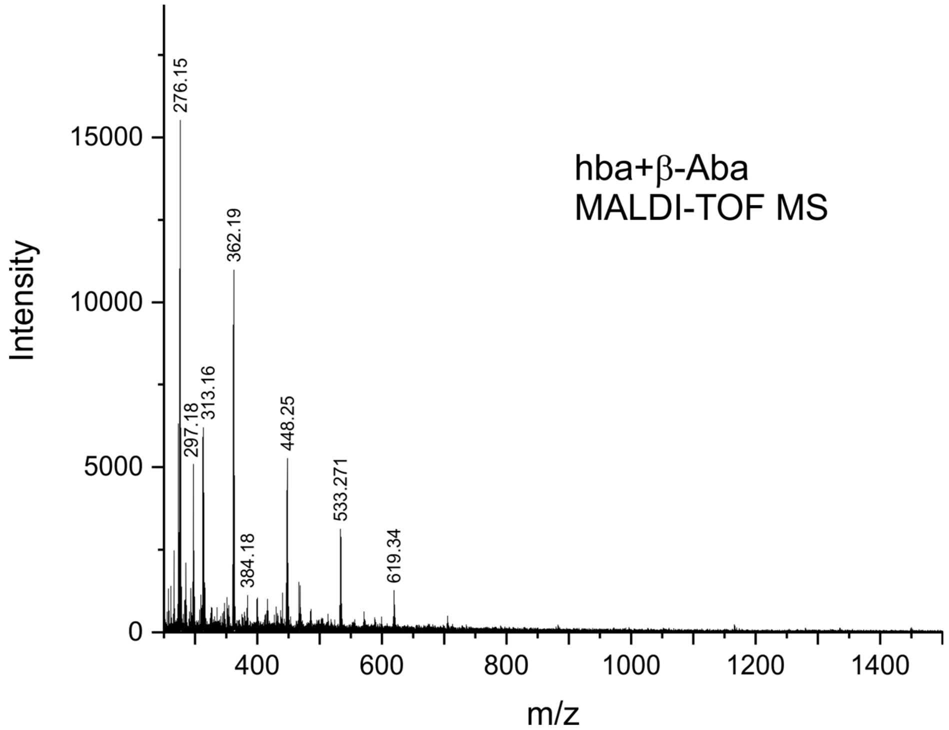

Figure S16. MS of a single-step dry-down reaction of 3-hydroxybutanoic acid (hba) and beta-aminobutyric acid (β-Aba) supports the formation of depsipeptides. hba and **β-Aba** were dried at 85 ˚C for seven days and the resulting depsipeptides were analyzed by negative-mode ESI-MS and MALDI-TOF MS, indicating a variety of depsipeptides. **hba** is labeled in red; **β-Aba** is labeled in green. All labeled species correspond to $[M-H]^-$ ions.

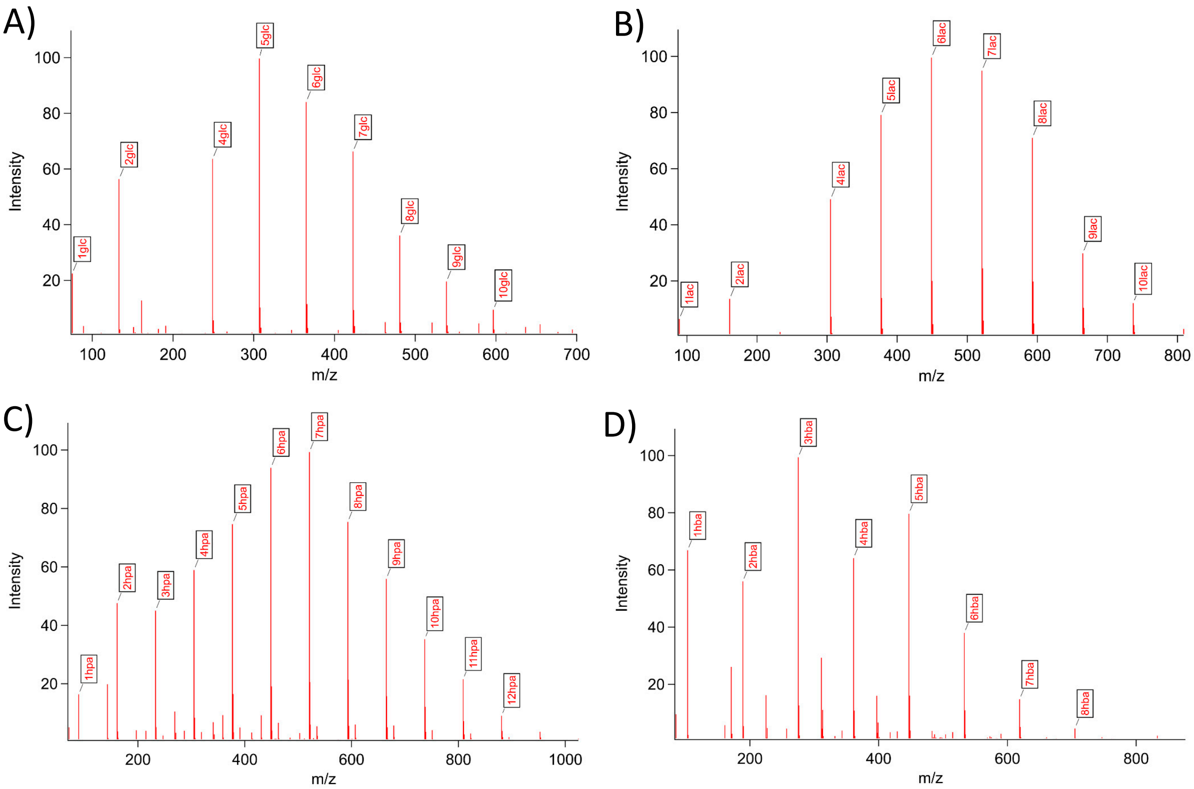

Figure S17. ESI-MS analysis of control single-step dry-down reactions of the hydroxy acids in the absence of amino acids. glycolic acid (**glc**, **A**), lactic acid (**lac**, **B**), 3-hydroxypropionic acid (**hpa**, **C**), and 3-hydroxybutanoic acid (**hba**, **D**) were dried at 85˚C for seven days and the resulting products were analyzed by negative-mode ESI-MS. MS analysis indicates the formation of a variety of polyesters. All labeled species correspond to $[M\text{-}H]^-$ ions.

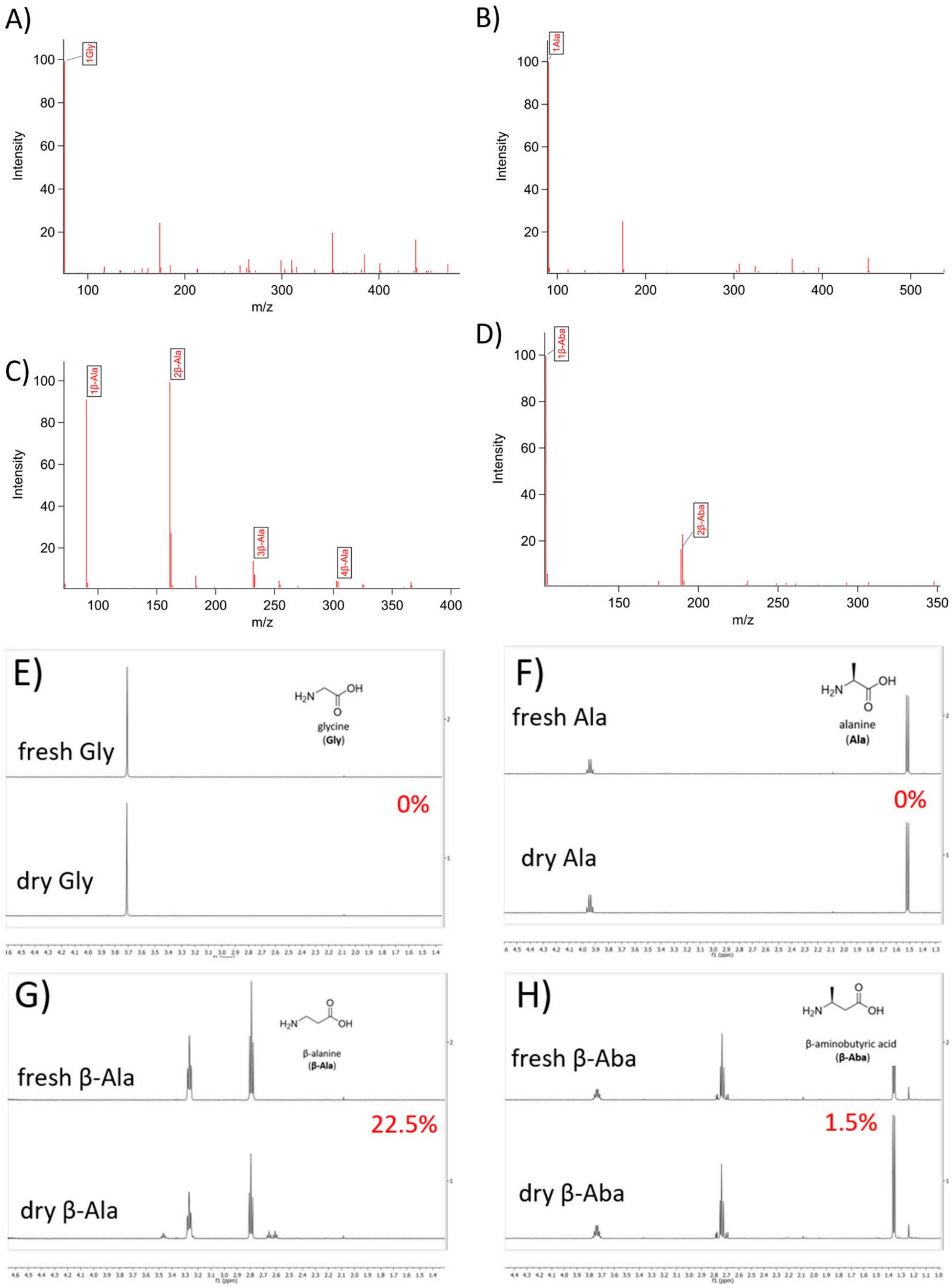

Figure S18. Analysis of control single-step dry-down reactions of the amino acids in the absence of hydroxy acids. Glycine (**Gly**, **A**), Alanine (**Ala**, **B**), β-alanine (**β-Ala**, **C**), and β-aminobutyric acid (**β-Aba**, **D**) were dried at 85˚C for seven days and the resulting products were analyzed by positive-mode ESI-MS (**A**-**D**). Labeled species correspond to $[M+H]^+$ ions. Short peptides were evident for beta amino acids single-step dry-down reactions, but no peptides were formed for the respective alpha amino acids controls that were subjected to the same reaction conditions. Similarly, individual amino acids were

dried at 85˚C for seven days under acidified conditions (pH~3) and the resulting products were analyzed by ^{1}H NMR (**E-H**). Percent conversion is indicated in each panel.

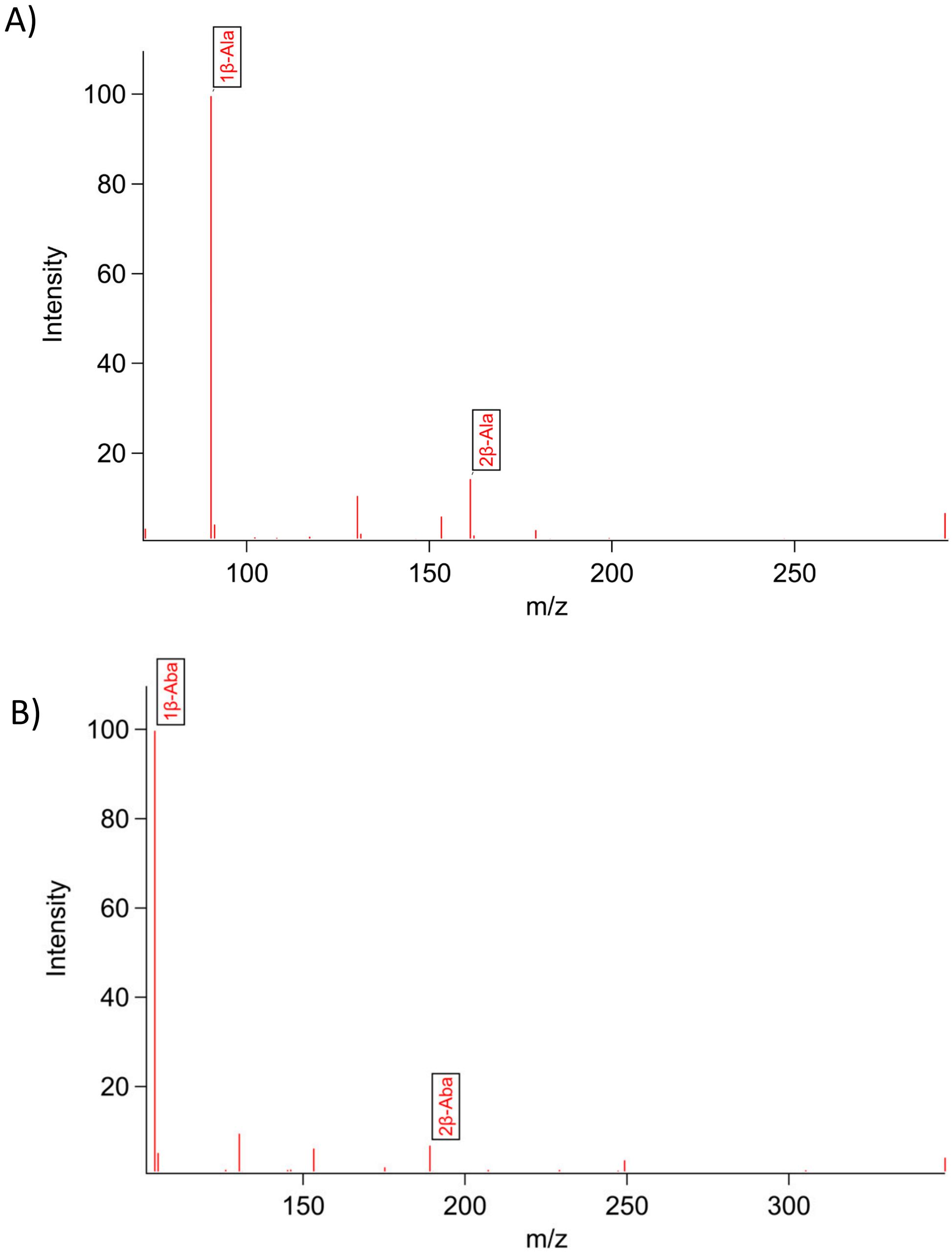

Figure S19. ESI-MS analysis of stock solutions of β-amino acids before single-step dry-down reactions shows the existence of some peptide dimers are present prior to reaction. β-alanine (**β-Ala**, **A**) and β-aminobutyric acid (**β-Aba**, **B**) stock solutions were analyzed by positive-mode ESI-MS.

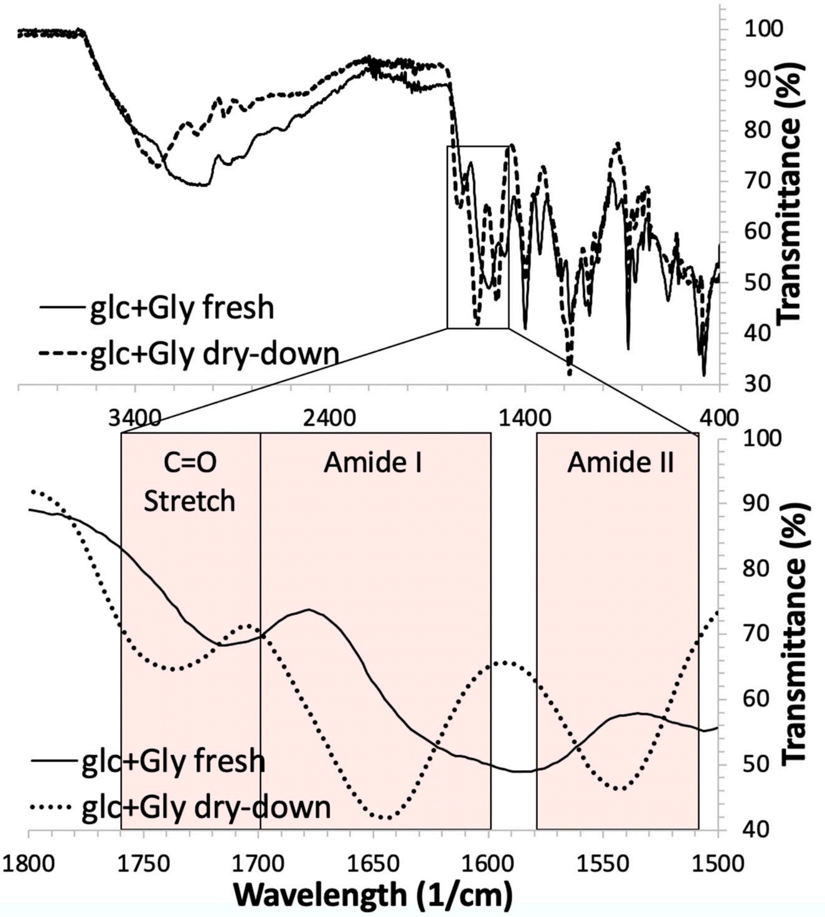

Figure S20. FTIR spectra changes upon single-step dry-down reactions of glycolic acid (glc) and glycine (Gly) support the formation of depsipeptides. glc and **Gly** were dried at 85 ˚C for seven days and the resulting depsipeptides were analyzed by FTIR and compared to unreacted stock solutions. The FTIR of the single-step dry-down reactions shows that the C=O stretch has shifted from ~1720 to 1740 cm^{-1}, consistent with conversion of a free carboxylic acid to an ester, and shifts are also evident in the amide I (1600 cm^{-1}-1700 cm^{-1}) and amide II (1510 cm^{-1}-1580 cm^{-1}) regions.

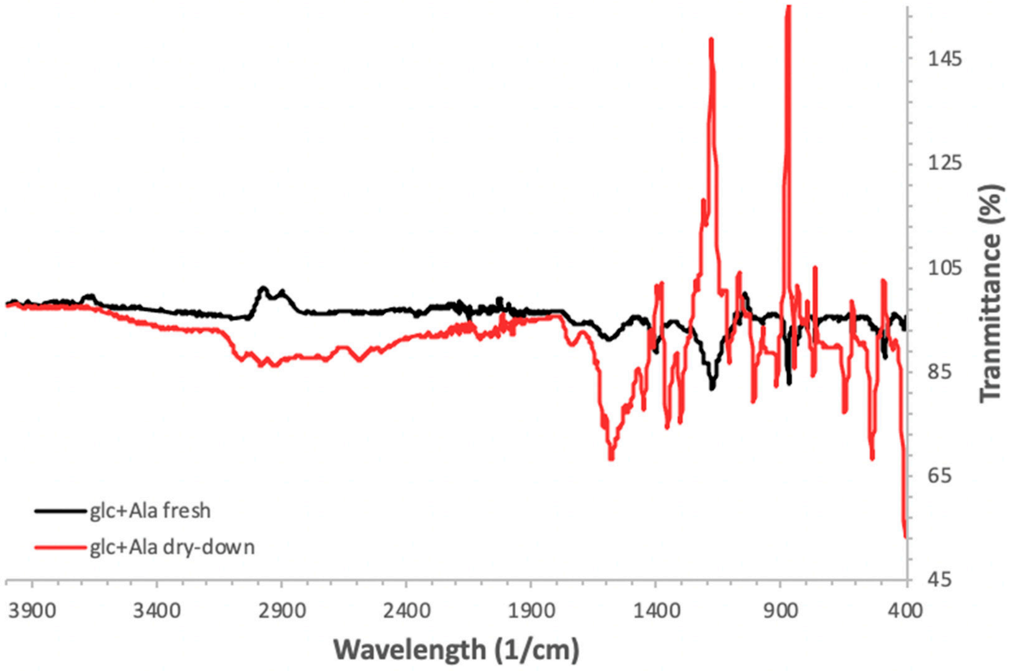

Figure S21. FTIR spectra changes upon single-step dry-down reactions of glycolic acid (glc) and alanine (Ala) support the formation of depsipeptides. glc and **Ala** were dried at 85 °C for seven days and the resulting depsipeptides were analyzed by FTIR and compared to unreacted stock solutions.

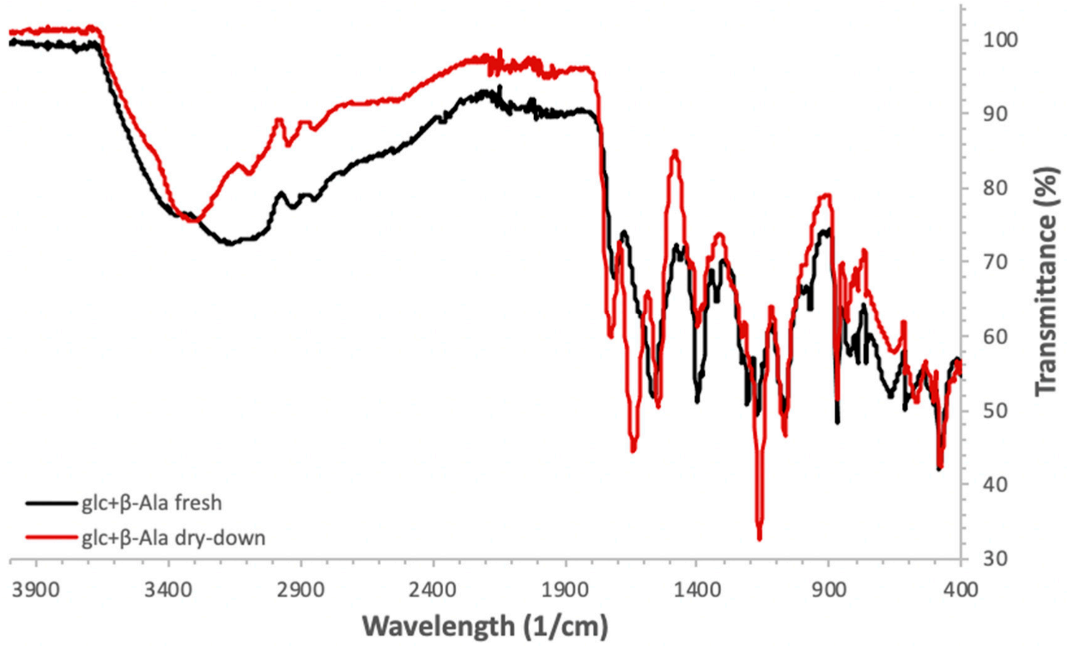

Figure S22. FTIR spectra changes upon single-step dry-down reactions of glycolic acid (glc) and beta-alanine (β-Ala) support the formation of depsipeptides. glc and **β-Ala** were dried at 85 ˚C for seven days and the resulting depsipeptides were analyzed by FTIR and compared to unreacted stock solutions.

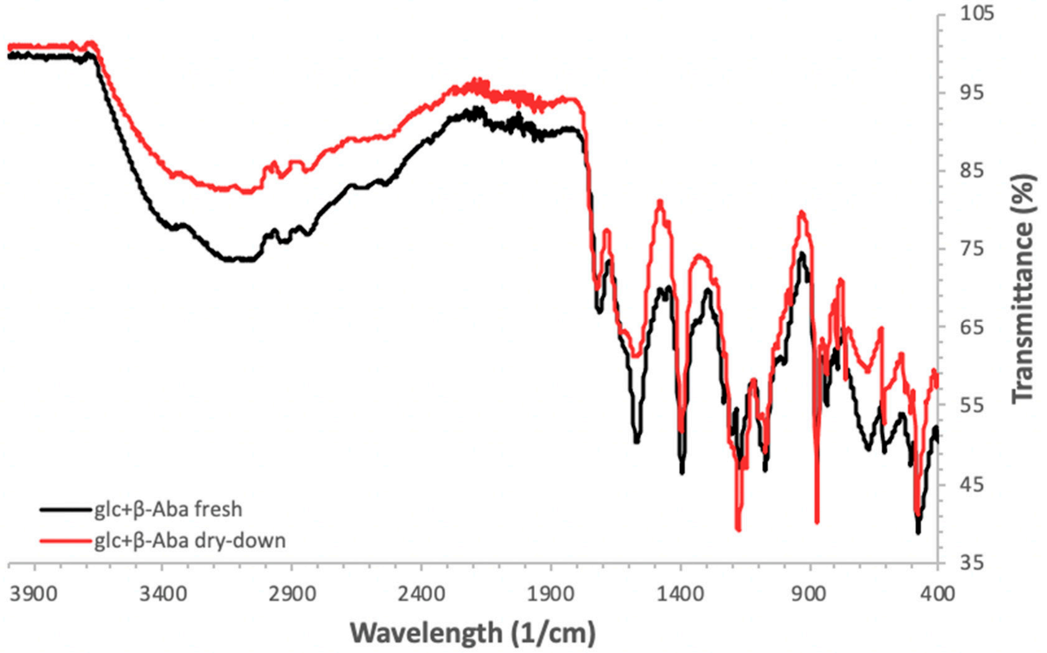

Figure S23. FTIR spectra changes upon single-step dry-down reactions of glycolic acid (glc) and beta-aminobutyric acid (β-Aba) support the formation of depsipeptides. glc and **β-Aba** were dried at 85 ˚C for seven days and the resulting depsipeptides were analyzed by FTIR and compared to unreacted stock solutions.

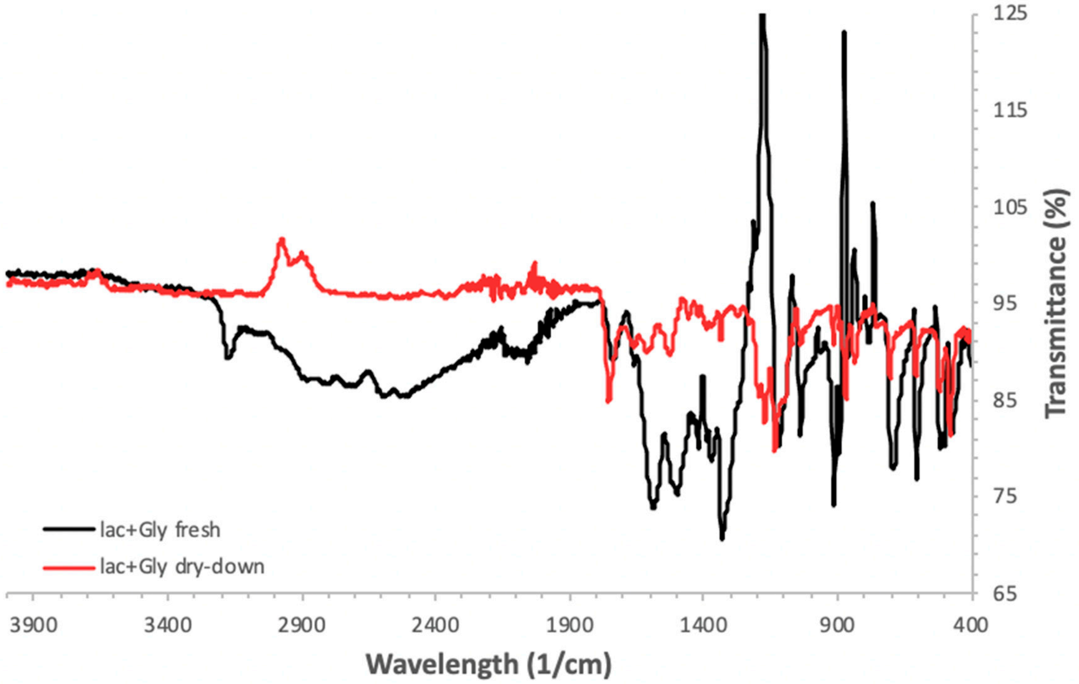

Figure S24. FTIR spectra changes upon single-step dry-down reactions of lactic acid (lac) and glycine (Gly) support the formation of depsipeptides. lac and **Gly** were dried at 85 ˚C for seven days and the resulting depsipeptides were analyzed by FTIR and compared to unreacted stock solutions.

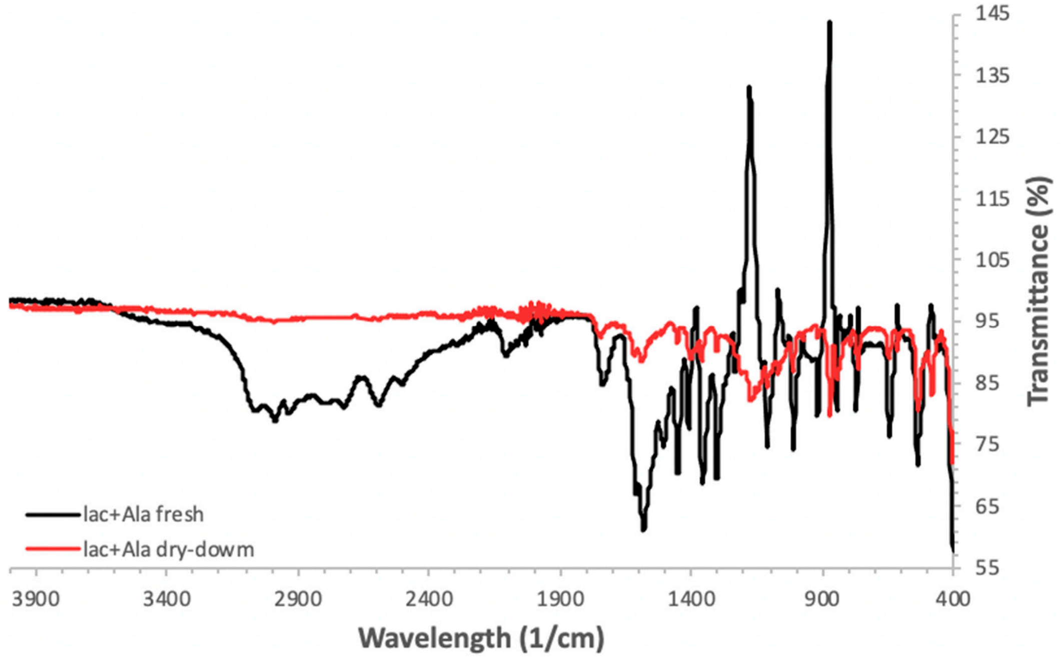

Figure S25. FTIR spectra changes upon single-step dry-down reactions of lactic acid (lac) and alanine (Ala) support the formation of depsipeptides. lac and **Ala** were dried at 85 ˚C for seven days and the resulting depsipeptides were analyzed by FTIR and compared to unreacted stock solutions.

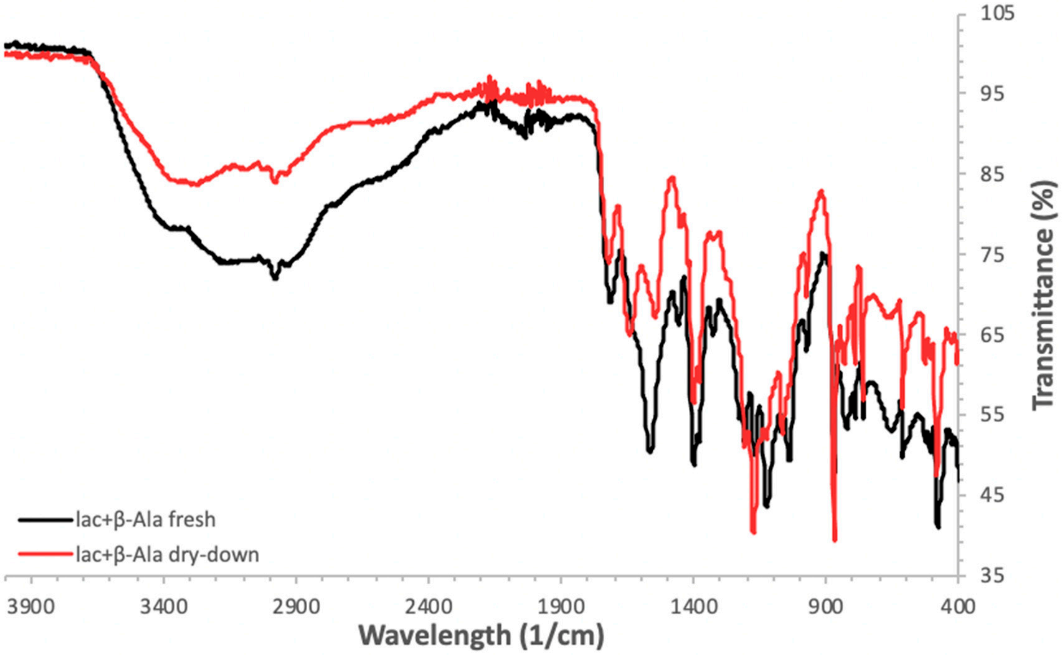

Figure S26. FTIR spectra changes upon single-step dry-down reactions of lactic acid (lac) and beta-alanine (β-Ala) support the formation of depsipeptides. lac and **β-Ala** were dried at 85 ˚C for seven days and the resulting depsipeptides were analyzed by FTIR and compared to unreacted stock solutions.

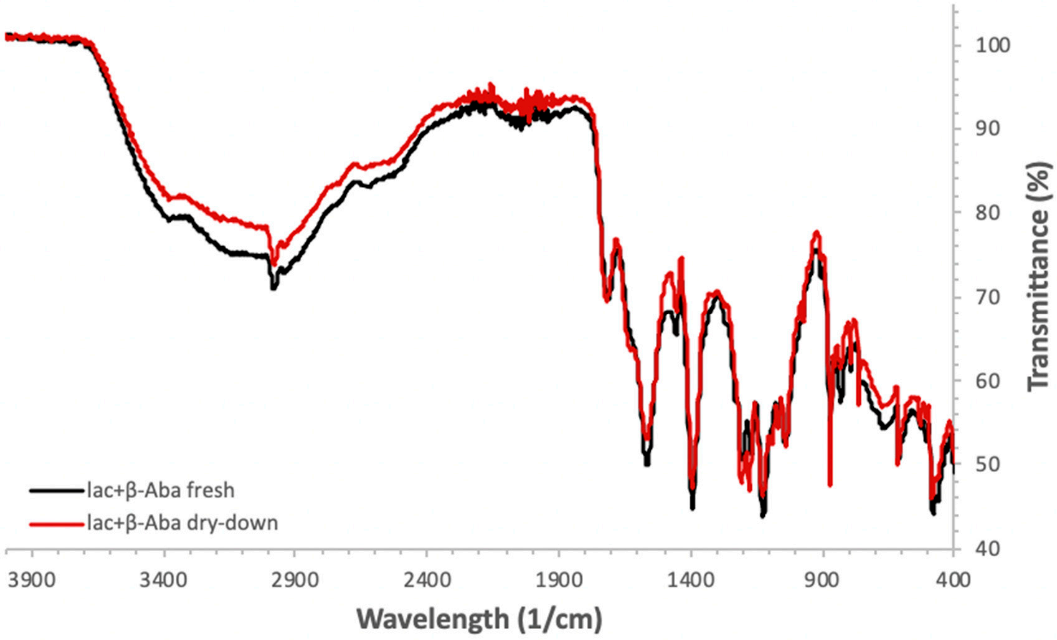

Figure S27. FTIR spectra changes upon single-step dry-down reactions of lactic acid (lac) and beta-aminobutryic acid (β-Aba) support the formation of depsipeptides. lac and **β-Aba** were dried at 85 ˚C for seven days and the resulting depsipeptides were analyzed by FTIR and compared to unreacted stock solutions.

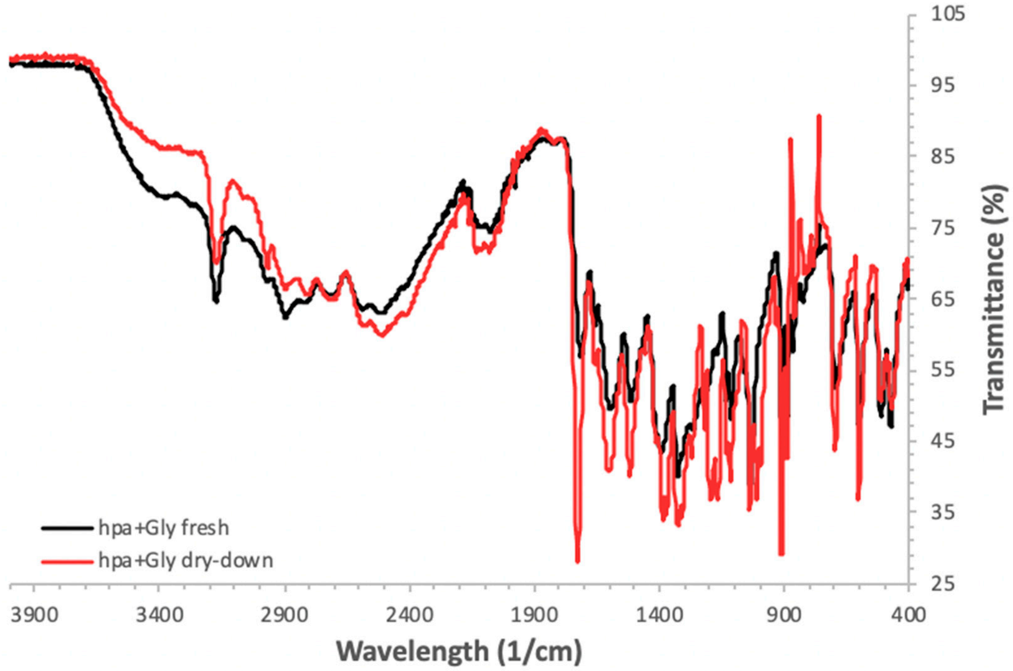

Figure S28. FTIR spectra changes upon single-step dry-down reactions of 3-hydroxypropanoic acid (hpa) and glycine (Gly) support the formation of depsipeptides. hpa and **Gly** were dried at 85 ˚C for seven days and the resulting depsipeptides were analyzed by FTIR and compared to unreacted stock solutions.

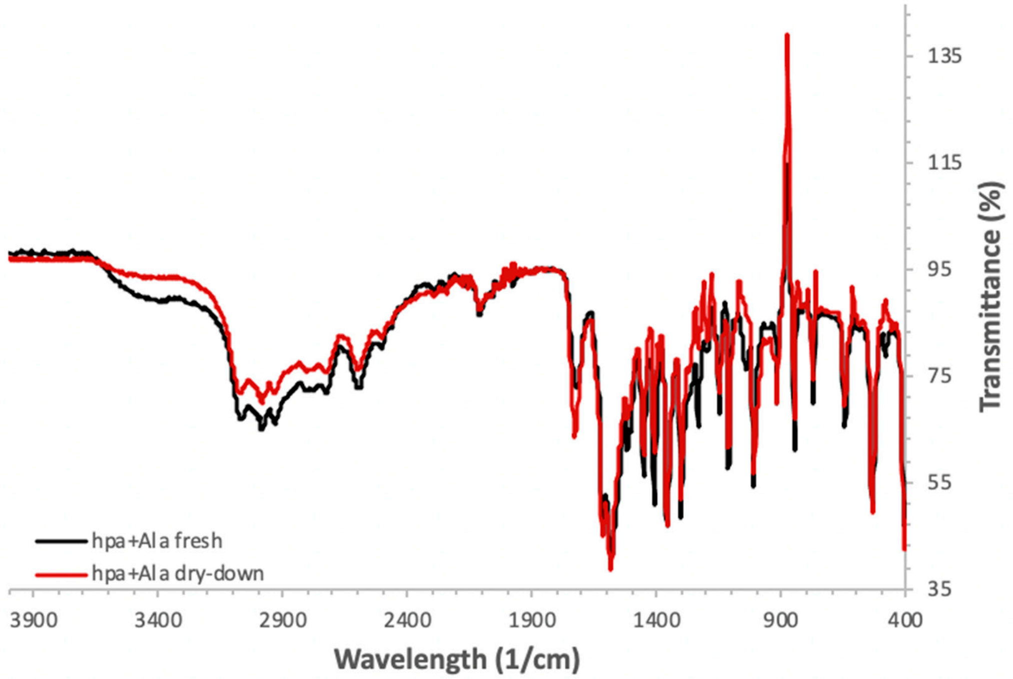

Figure S29. FTIR spectra changes upon single-step dry-down reactions of 3-hydroxypropanoic acid (hpa) and alanine (Ala) support the formation of depsipeptides. hpa and **Ala** were dried at 85 ˚C for seven days and the resulting depsipeptides were analyzed by FTIR and compared to unreacted stock solutions.

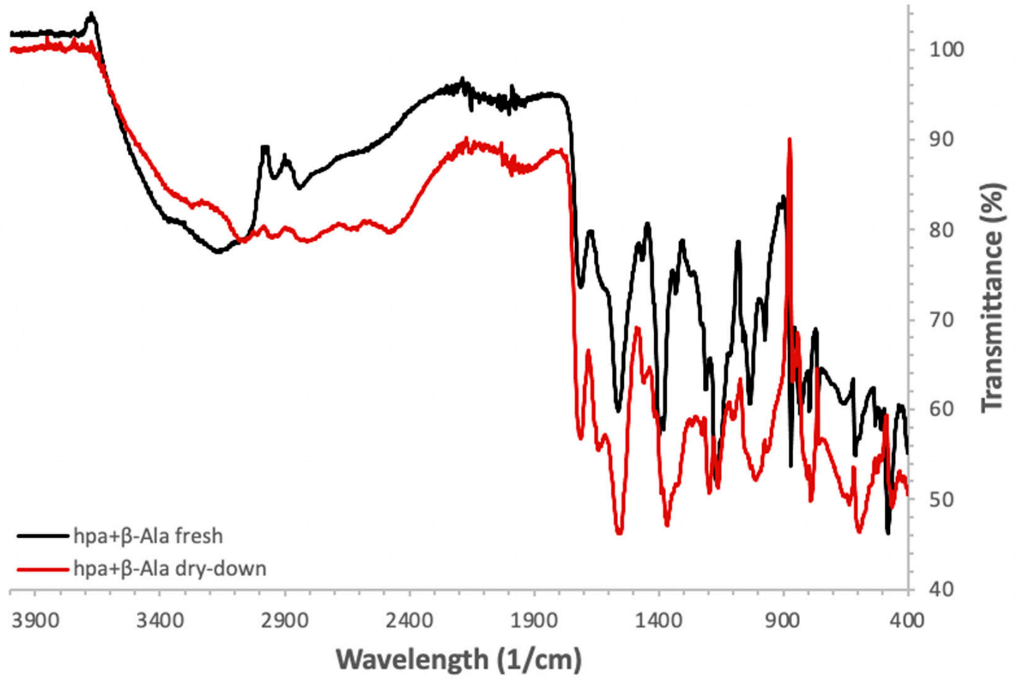

Figure 30. FTIR spectra changes upon single-step dry-down reactions of 3-hydroxypropanoic acid (hpa) and beta-alanine (β-Ala) support the formation of depsipeptides. hpa and **β-Ala** were dried at 85 ˚C for seven days and the resulting depsipeptides were analyzed by FTIR and compared to unreacted stock solutions.

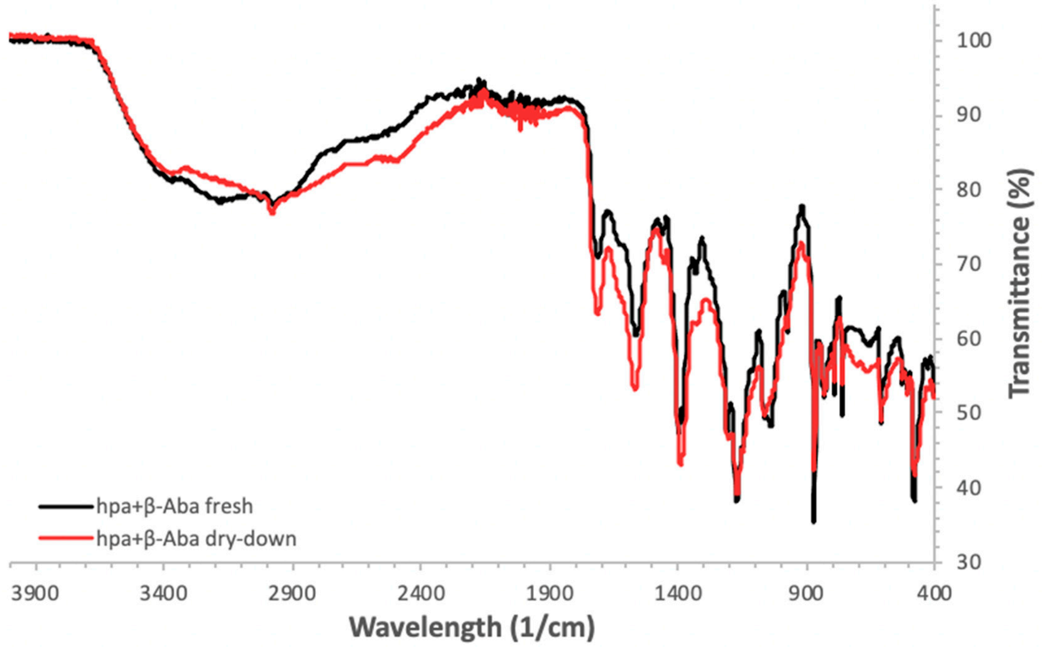

Figure S31. FTIR spectra changes upon single-step dry-down reactions of 3-hydroxypropanoic acid (hpa) and beta-aminobutyric acid (β-Aba) support the formation of depsipeptides. hpa and **β-Aba** were dried at 85 ˚C for seven days and the resulting depsipeptides were analyzed by FTIR and compared to unreacted stock solutions.

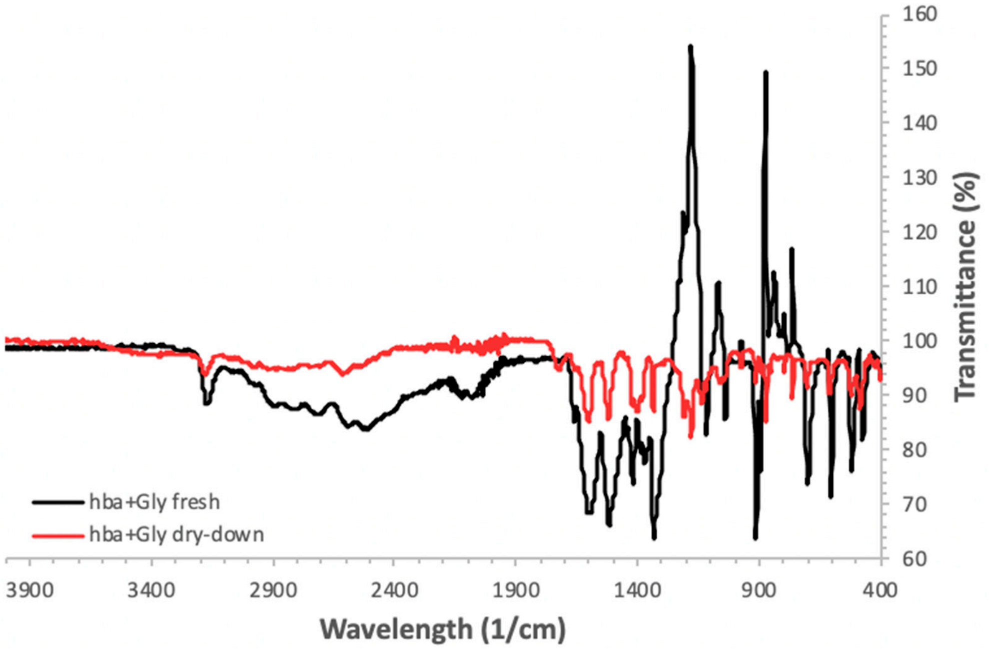

Figure S32. FTIR spectra changes upon single-step dry-down reactions of 3-hydroxybutanoic acid (hba) and glycine (Gly) support the formation of depsipeptides. **hba** and **Gly** were dried at 85 ˚C for seven days and the resulting depsipeptides were analyzed by FTIR and compared to unreacted stock solutions.

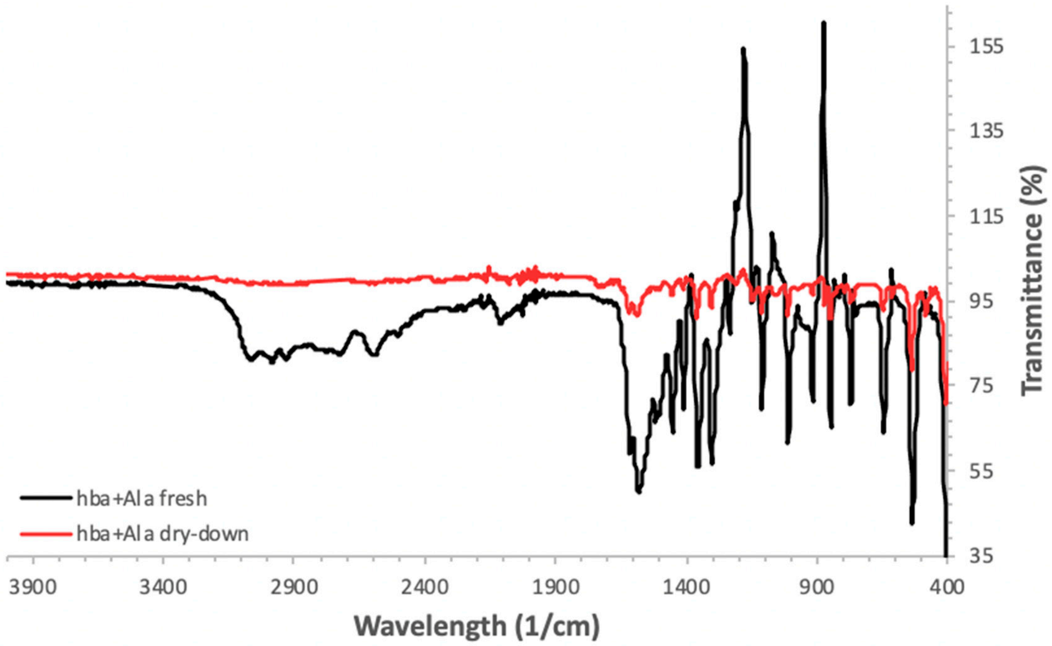

Figure S33. FTIR spectra changes upon single-step dry-down reactions of 3-hydroxybutanoic acid (hba) and alanine (Ala) support the formation of depsipeptides. **hba** and **Ala** were dried at 85 ˚C for seven days and the resulting depsipeptides were analyzed by FTIR and compared to unreacted stock solutions.

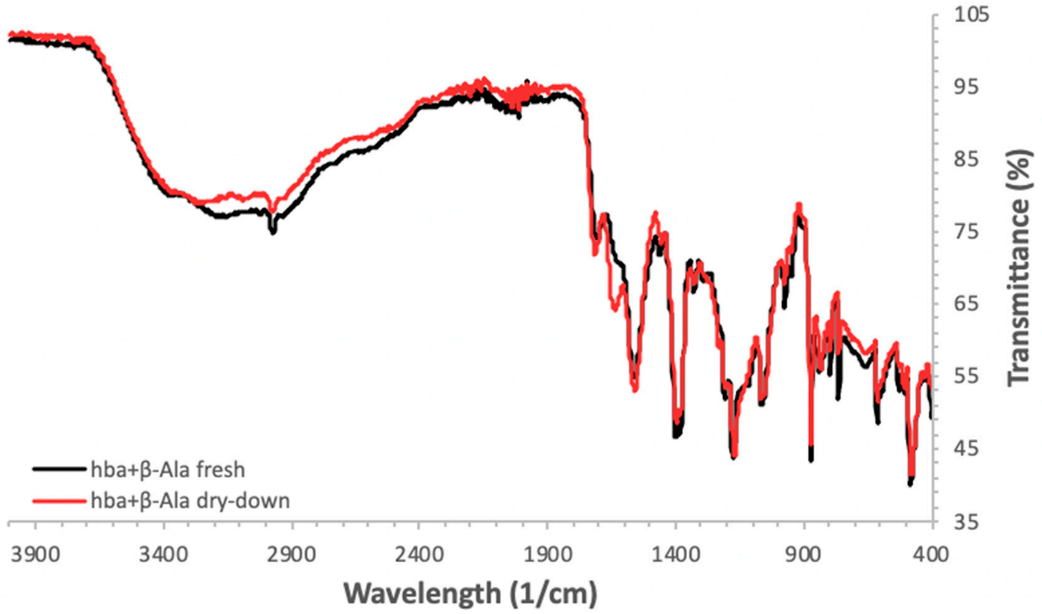

Figure S34. FTIR spectra changes upon single-step dry-down reactions of 3-hydroxybutanoic acid (hba) and beta-alanine (β-Ala) support the formation of depsipeptides. hba and **β-Ala** were dried at 85 ˚C for seven days and the resulting depsipeptides were analyzed by FTIR and compared to unreacted stock solutions.

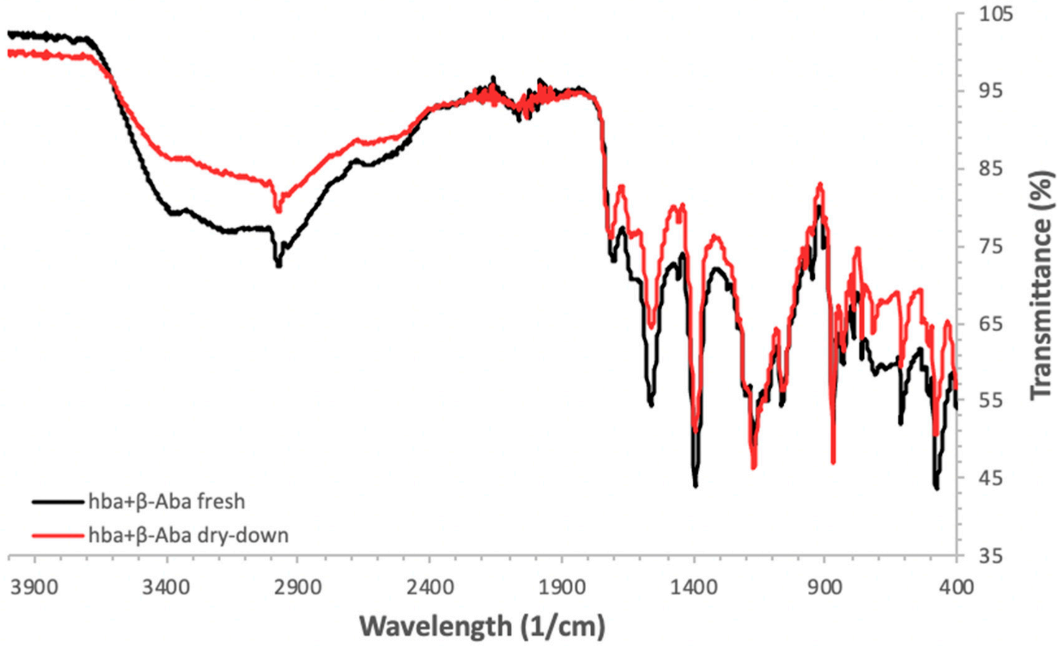

Figure S35. FTIR spectra changes upon single-step dry-down reactions of 3-hydroxybutanoic acid (hba) and beta-aminobutyric acid (β-Aba) support the formation of depsipeptides. hba and **β-Aba** were dried at 85 ˚C for seven days and the resulting depsipeptides were analyzed by FTIR and compared to unreacted stock solutions.

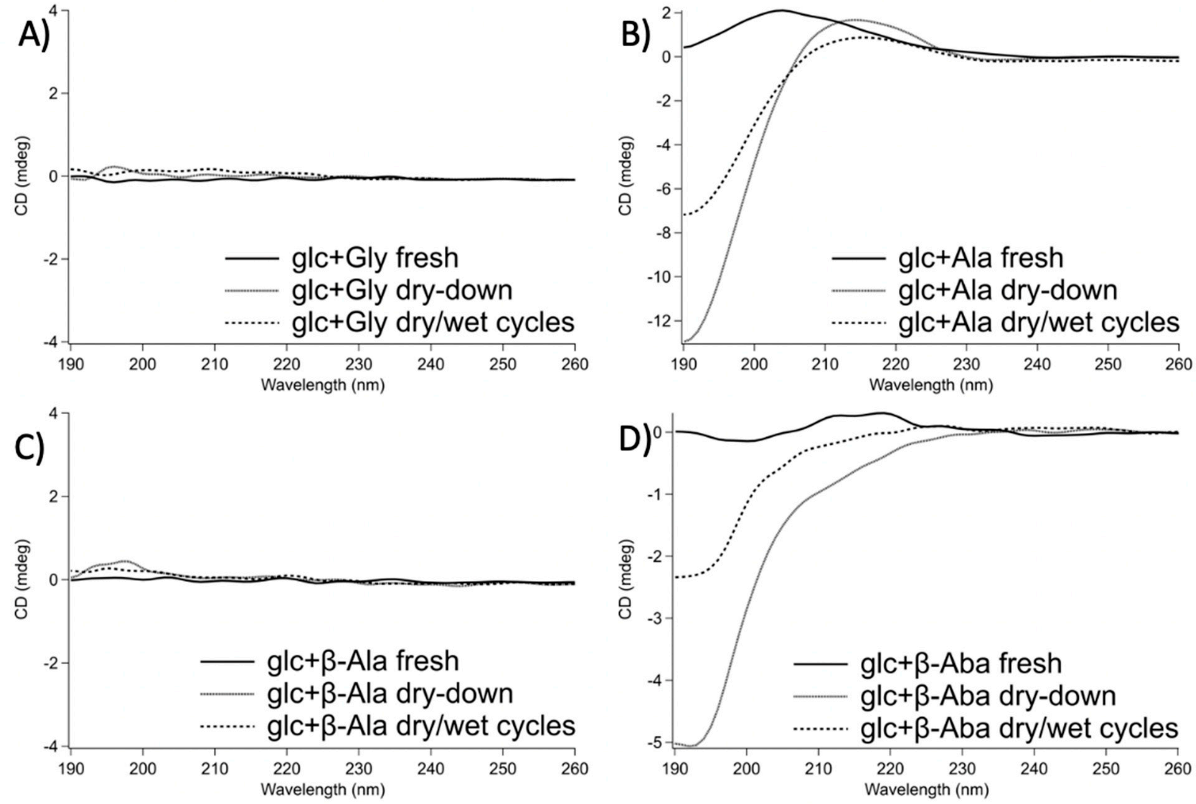

Figure S36. Circular Dichroism spectra of glycolic acid (glc) single-step dry-down reactions and dry/wet cycling reactions with the amino acids. glc and either **Gly** (**A**), **Ala** (**B**), **β-Ala** (**C**), and **β-Aba** (**D**) were either subjected to single-step dry-down or to daily dry/wet cycling (16 hr dry/8 hr wet) for seven days at 85 ˚C and then analyzed by circular dichroism.

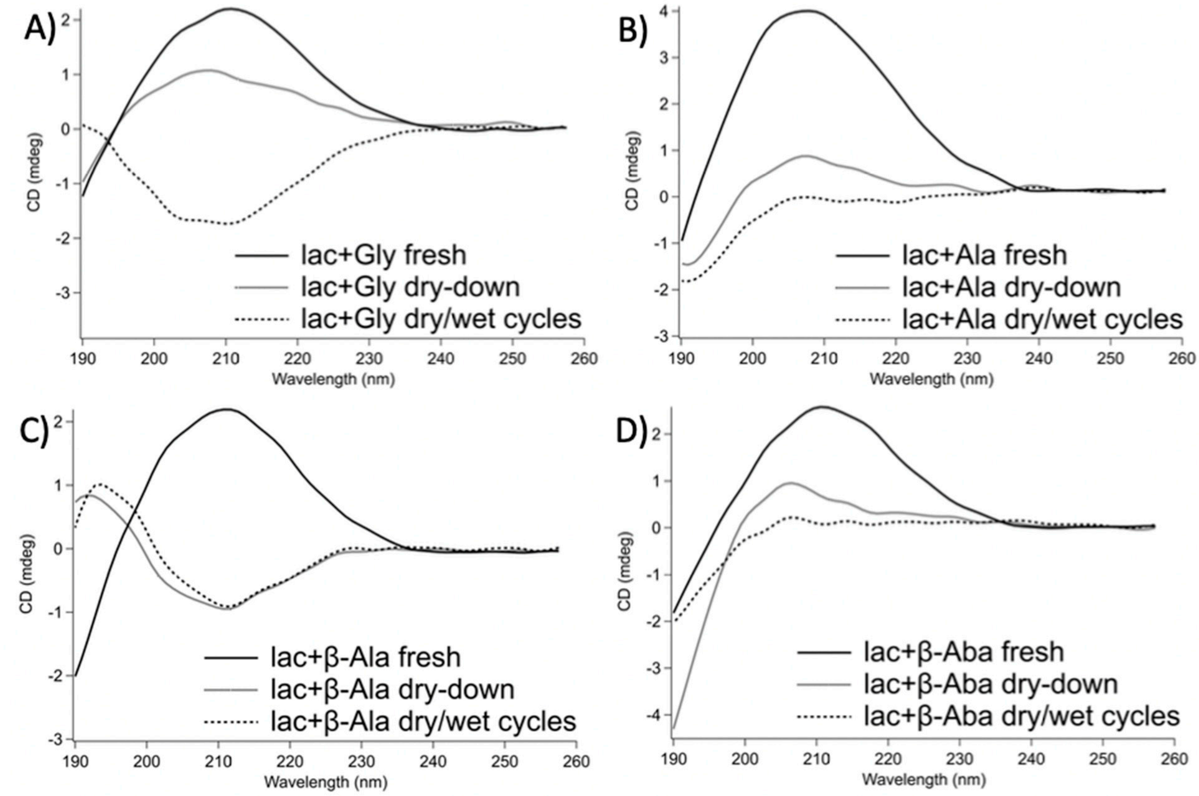

Figure S37. Circular Dichroism spectra of lactic acid (lac) single-step dry-down reactions and dry/wet cycling reactions with the amino acids. lac and either **Gly** (**A**), **Ala** (**B**), **β-Ala** (**C**), and **β-Aba** (**D**) were either subjected to single-step dry-down or to daily dry/wet cycling (16 hr dry/8 hr wet) for seven days at 85 ˚C and then analyzed by circular dichroism.

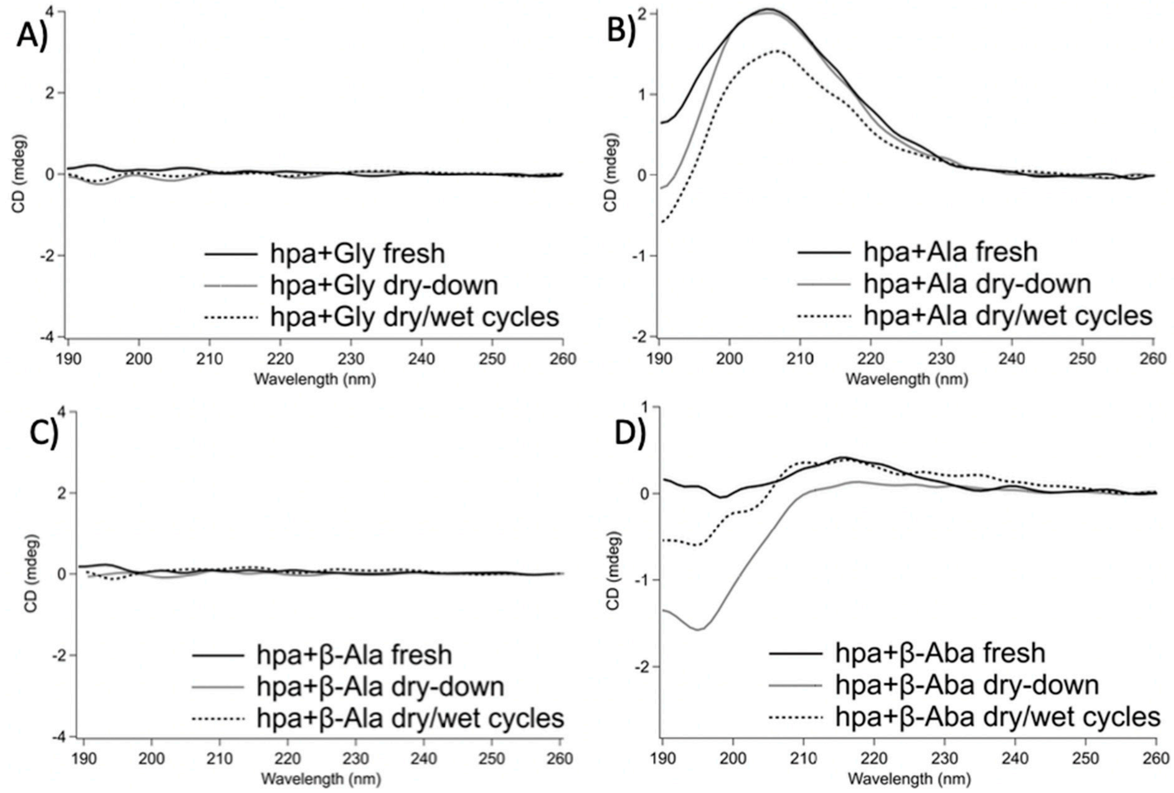

Figure S38. Circular Dichroism spectra of 3-hydroxypropanoic acid (hpa) single-step dry-down reactions and dry/wet cycling reactions with the amino acids. hpa and either **Gly** (**A**), **Ala** (**B**), **β-Ala** (**C**), and **β-Aba** (**D**) were either subjected to single-step dry-down or to daily dry/wet cycling (16 hr dry/8 hr wet) for seven days at 85 °C and then analyzed by circular dichroism.

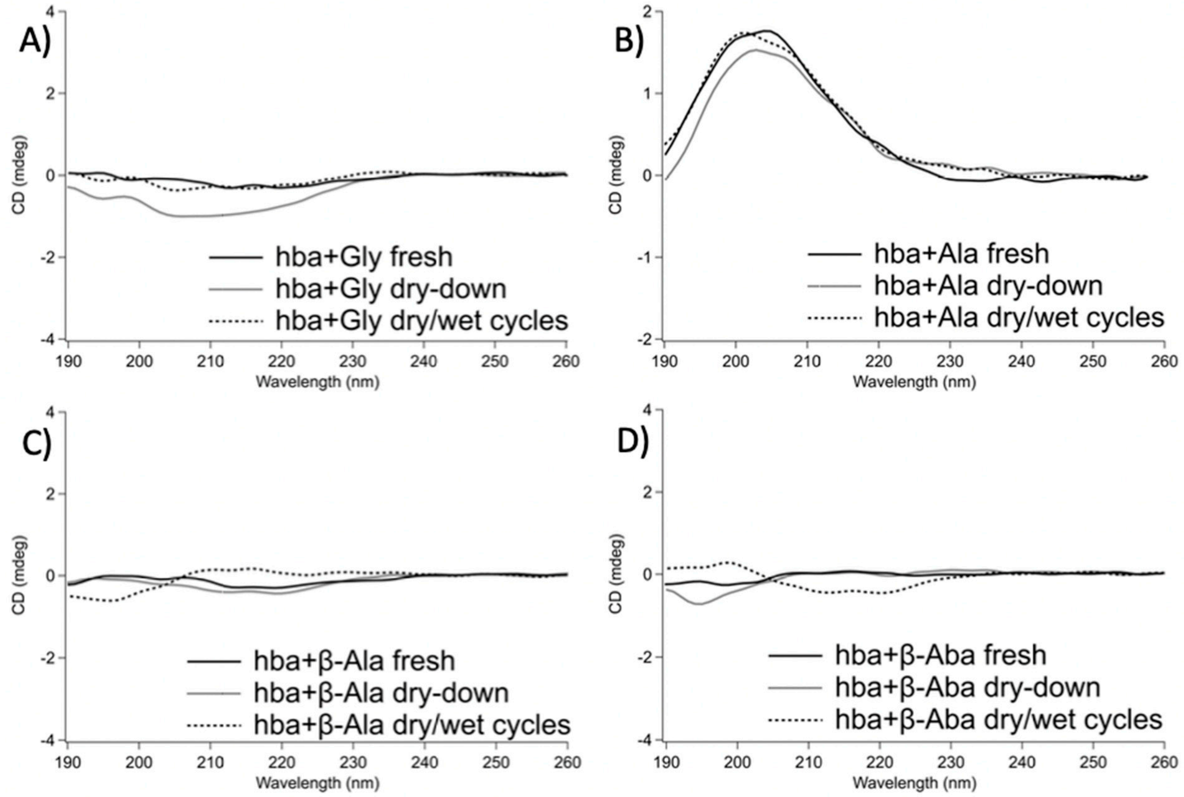

Figure S39. Circular Dichroism spectra of 3-hydroxybutanoic acid (hba) single-step dry-down reactions and dry/wet cycling reactions with the amino acids. hba and either **Gly** (**A**), **Ala** (**B**), **β-Ala** (**C**), and **β-Aba** (**D**) were either subjected to single-step dry-down or to daily dry/wet cycling (16 hr dry/8 hr wet) for seven days at 85 ˚C and then analyzed by circular dichroism.

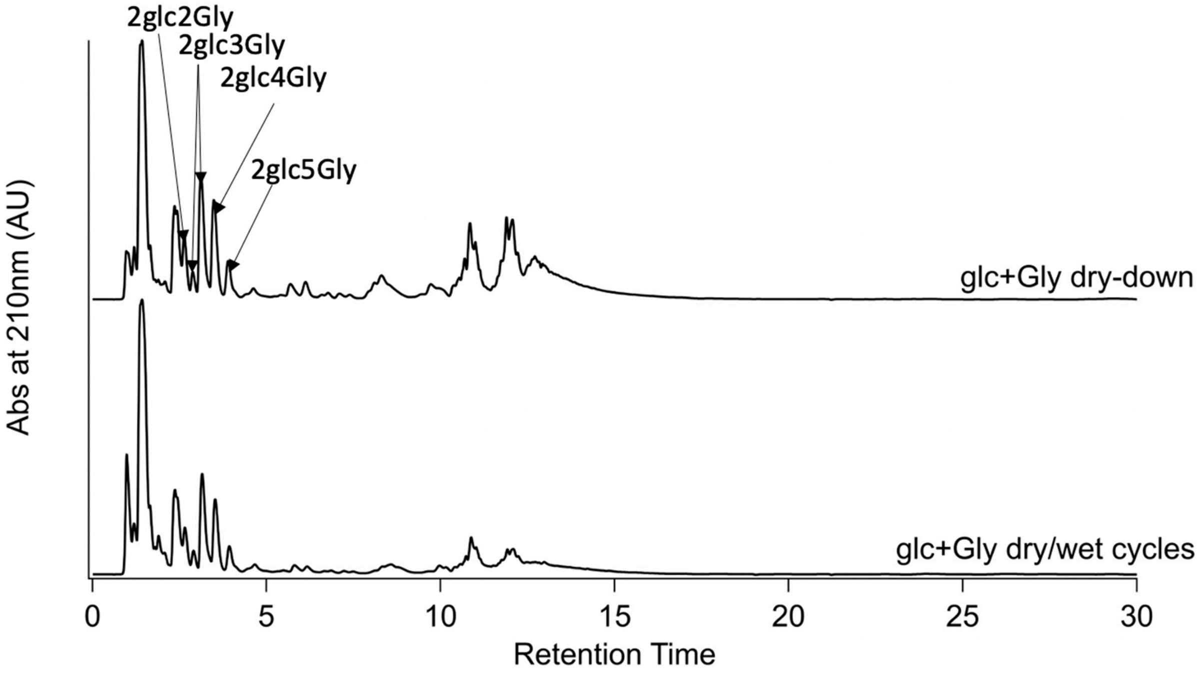

Figure S40. C18-HPLC analysis verifies the formation of depsipeptides following single-step dry-down reactions and dry/wet cycling reactions of glycolic acid (glc) and glycine (Gly). A mixture of **glc** and **Gly** was subjected to single-step dry-down at 85 ˚C or to daily dry/wet cycling (16 hr dry/8 hr wet) for seven days. The resulting depsipeptides were analyzed by hydrophobicity-based separation using C18-HPLC and the peaks were identified by LC-MS. Some of the identified peaks are labelled. Note that in many cases there are several species that co-elute, and the assignments are not necessarily denoting pure peptides/depsipeptides that are associated with the labelled peaks. The presence of various peaks verifies the heterogenous nature of the product mixture.

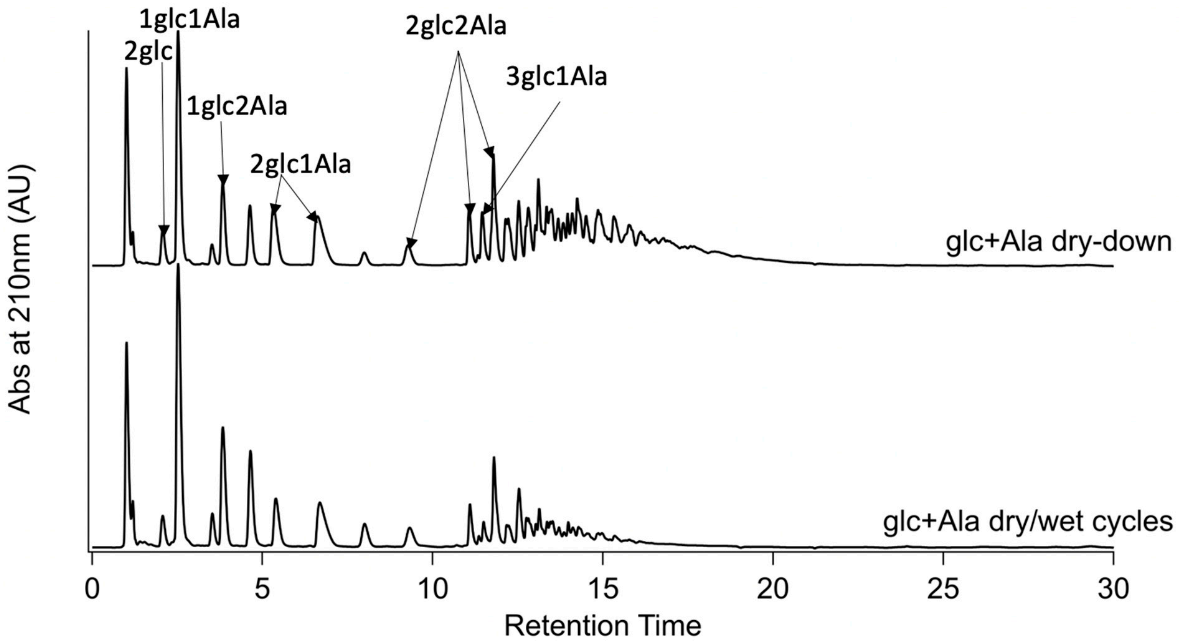

Figure S41. C18-HPLC analysis verifies the formation of depsipeptides following single-step dry-down reactions and dry/wet cycling reactions of glycolic acid (glc) and alanine (Ala). A mixture of **glc** and **Ala** was subjected to single-step dry-down at 85 °C or to daily dry/wet cycling (16 hr dry/8 hr wet) for seven days. The resulting depsipeptides were analyzed by hydrophobicity-based separation using C18-HPLC and the peaks were identified by LC-MS. The presence of various peaks verifies the

heterogenous nature of the product mixture and provides a relative abundance of the various polymers.

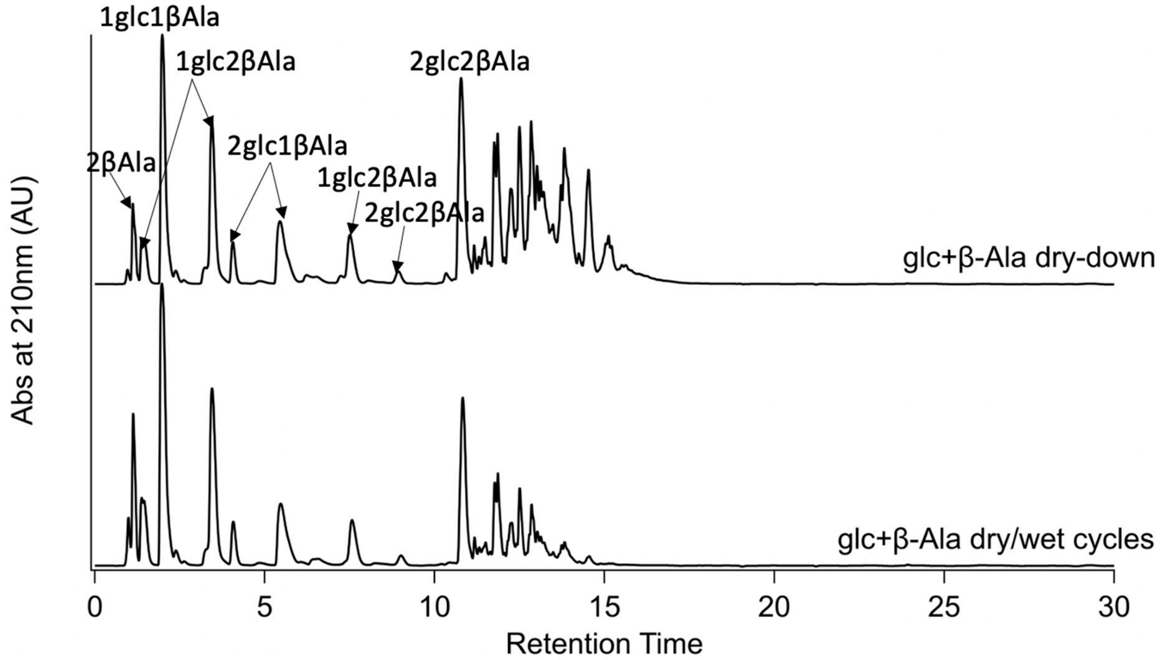

Figure S42. C18-HPLC analysis verifies the formation of depsipeptides following single-step dry-down reactions and dry/wet cycling reactions of glycolic acid (glc) and

beta-alanine (β-Ala). A mixture of **glc** and **β-Ala** was subjected to single-step dry-down at 85 ˚C or to daily dry/wet cycling (16 hr dry/8 hr wet) for seven days. The resulting depsipeptides were analyzed by hydrophobicity-based separation using C18-HPLC and the peaks were identified by LC-MS. The presence of various peaks verifies the heterogenous nature of the product mixture and provides a relative abundance of the various polymers.

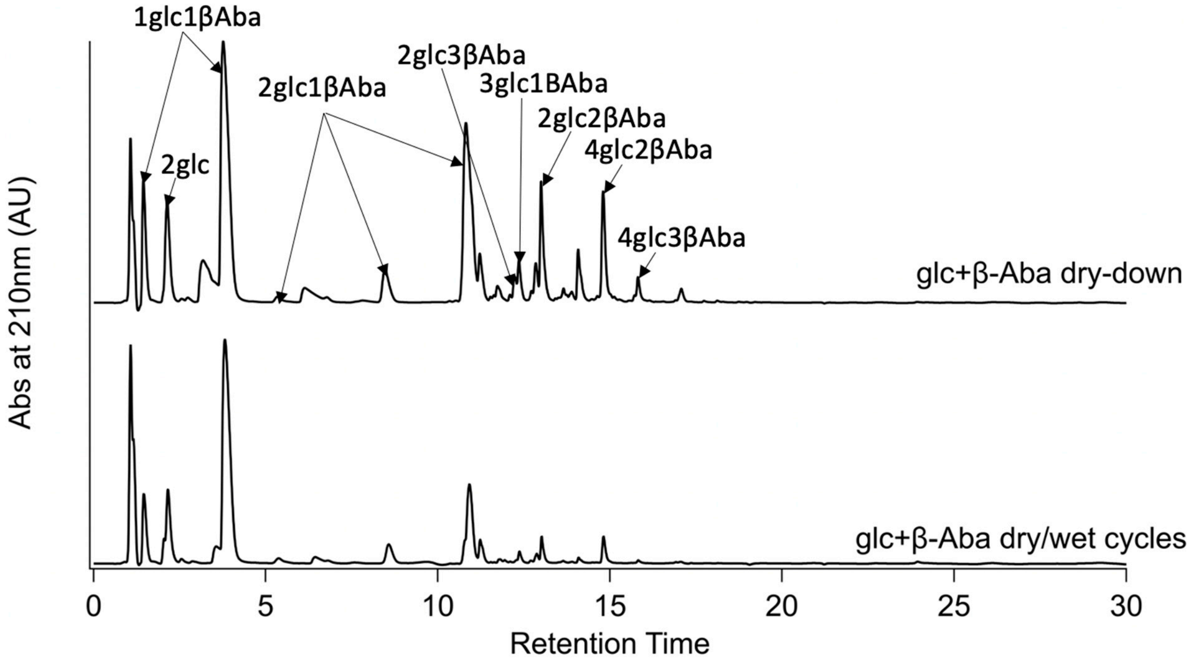

Figure S43. C18-HPLC analysis verifies the formation of depsipeptides following single-step dry-down reactions and dry/wet cycling reactions of glycolic acid (glc) and beta-aminobutyric acid (β-Aba). A mixture of **glc** and **β-Aba** was subjected to single-step dry-down at 85 ˚C or to daily dry/wet cycling (16 hr dry/8 hr wet) for seven days. The resulting depsipeptides were analyzed by hydrophobicity-based separation using C18-HPLC and the peaks were identified by LC-MS. The presence of various peaks verifies the heterogenous nature of the product mixture and provides a relative abundance of the various polymers.

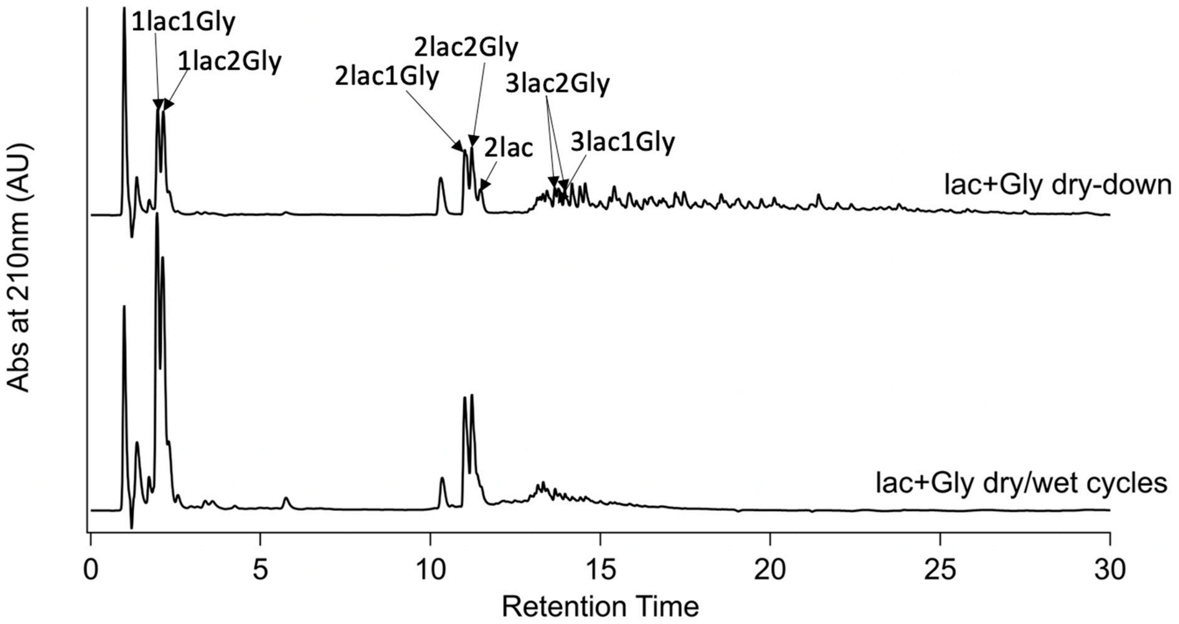

Figure S44. C18-HPLC analysis verifies the formation of depsipeptides following single-step dry-down reactions and dry/wet cycling reactions of lactic acid (lac) and glycine (Gly). A mixture of **glc** and **Gly** was subjected to single-step dry-down at 85 ˚C or to daily dry/wet cycling (16 hr dry/8 hr wet) for seven days. The resulting depsipeptides were analyzed by hydrophobicity-based separation using C18-HPLC and the peaks were identified by LC-MS. The presence of various peaks verifies the heterogenous nature of the product mixture and provides a relative abundance of the various polymers.

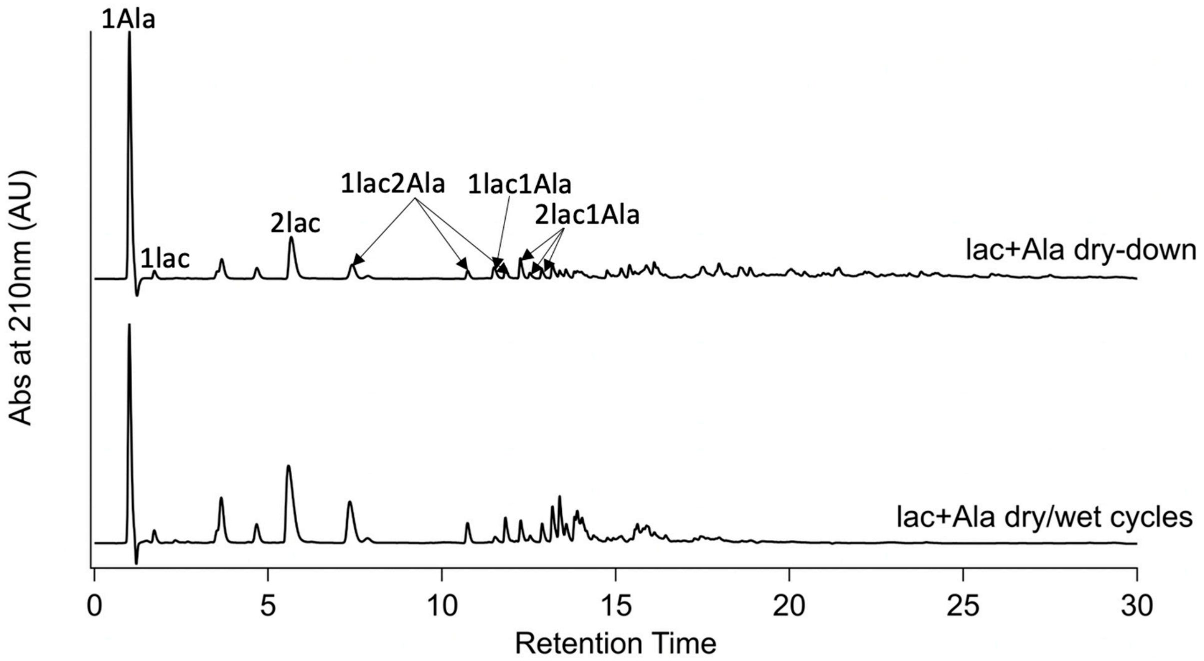

Figure S45. C18-HPLC analysis verifies the formation of depsipeptides following single-step dry-down reactions and dry/wet cycling reactions of lactic acid (lac) and alanine (Ala). A mixture of **glc** and **Ala** was subjected to single-step dry-down at 85 ˚C or to daily dry/wet cycling (16 hr dry/8 hr wet) for seven days. The resulting depsipeptides were analyzed by hydrophobicity-based separation using C18-HPLC and the peaks were identified by LC-MS. The presence of various peaks verifies the heterogenous nature of the product mixture and provides a relative abundance of the various polymers.

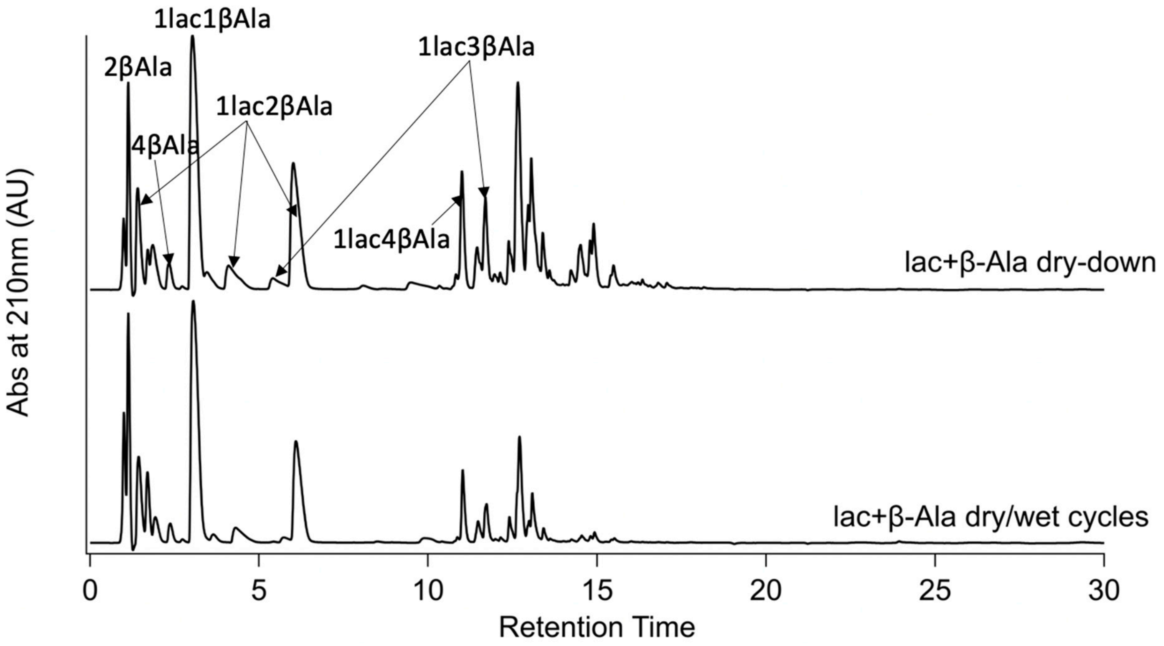

Figure S46. C18-HPLC analysis verifies the formation of depsipeptides following single-step dry-down reactions and dry/wet cycling reactions of lactic acid (lac) and beta-alanine (β-Ala). A mixture of **glc** and **β-Ala** was subjected to single-step dry-down at 85 °C or to daily dry/wet cycling (16 hr dry/8 hr wet) for seven days. The resulting depsipeptides were analyzed by hydrophobicity-based separation using C18-HPLC and the peaks were identified by LC-MS. The presence of various peaks verifies the heterogenous nature of the product mixture and provides a relative abundance of the various polymers.

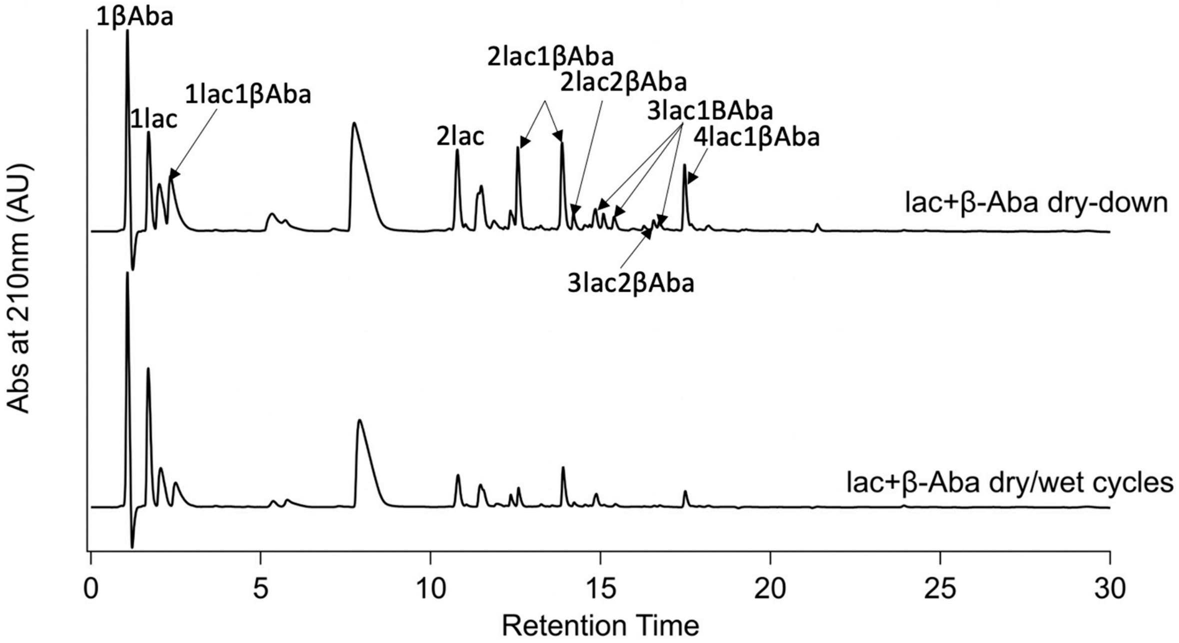

Figure S47. C18-HPLC analysis verifies the formation of depsipeptides following single-step dry-down reactions and dry/wet cycling reactions of lactic acid (lac) and beta-aminobutyric acid (β-Aba). A mixture of **glc** and **β-Aba** was subjected to single-step dry-down at 85 ˚C or to daily dry/wet cycling (16 hr dry/8 hr wet) for seven days. The resulting depsipeptides were analyzed by hydrophobicity-based separation using C18-HPLC and the peaks were identified by LC-MS. The presence of various peaks verifies the heterogenous nature of the product mixture and provides a relative abundance of the various polymers.

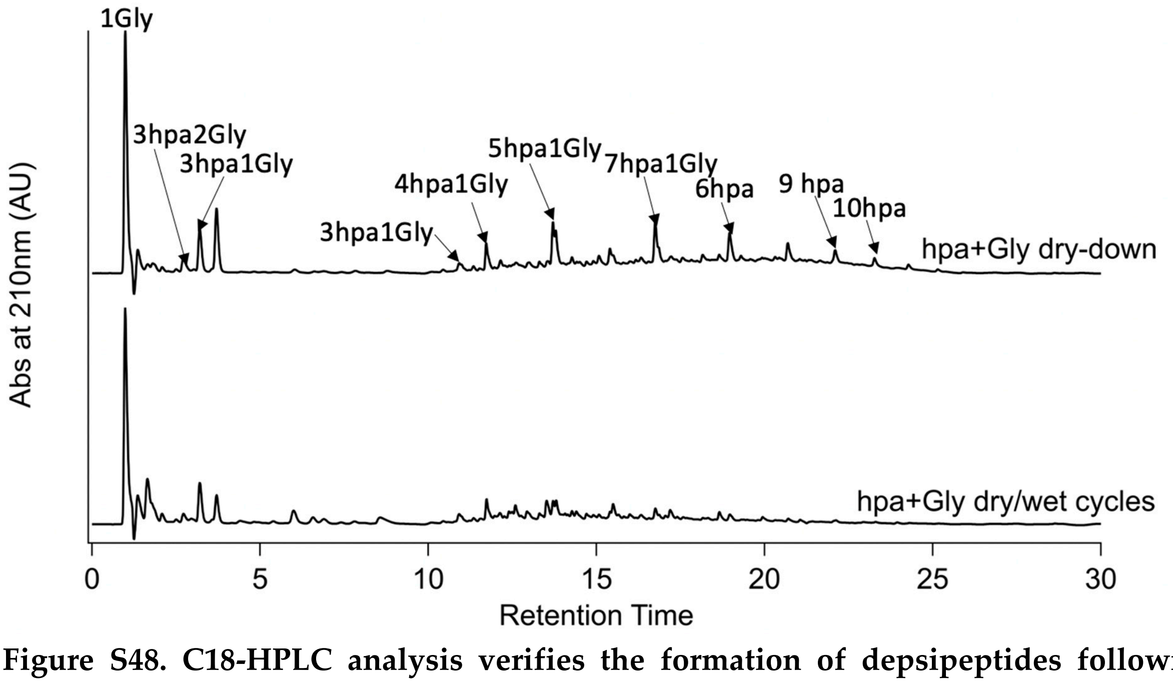

Figure S48. C18-HPLC analysis verifies the formation of depsipeptides following single-step dry-down reactions and dry/wet cycling reactions of 3-hydroxypropanoic acid (hpa) and glycine (Gly). A mixture of **hpa** and **Gly** was subjected to single-step dry-down at 85 ˚C or to daily dry/wet cycling (16 hr dry/8 hr wet) for seven days. The resulting depsipeptides were analyzed by hydrophobicity-based separation using C18-HPLC and the peaks were identified by LC-MS. The presence of various peaks verifies the

heterogenous nature of the product mixture and provides a relative abundance of the various polymers.

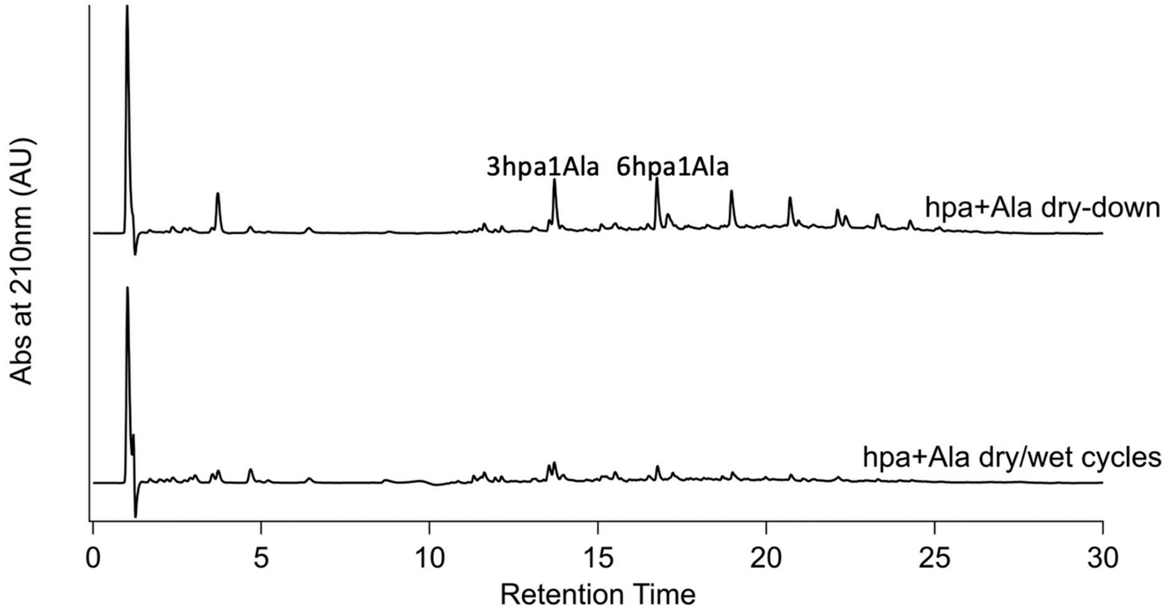

Figure S49. C18-HPLC analysis verifies the formation of depsipeptides following single-step dry-down reactions and dry/wet cycling reactions of 3-hydroxypropanoic

acid (hpa) and alanine (Ala). A mixture of **hpa** and **Ala** was subjected to single-step dry-down at 85 ˚C or to daily dry/wet cycling (16 hr dry/8 hr wet) for seven days. The resulting depsipeptides were analyzed by hydrophobicity-based separation using C18-HPLC and the peaks were identified by LC-MS. The presence of various peaks verifies the heterogenous nature of the product mixture and provides a relative abundance of the various polymers.

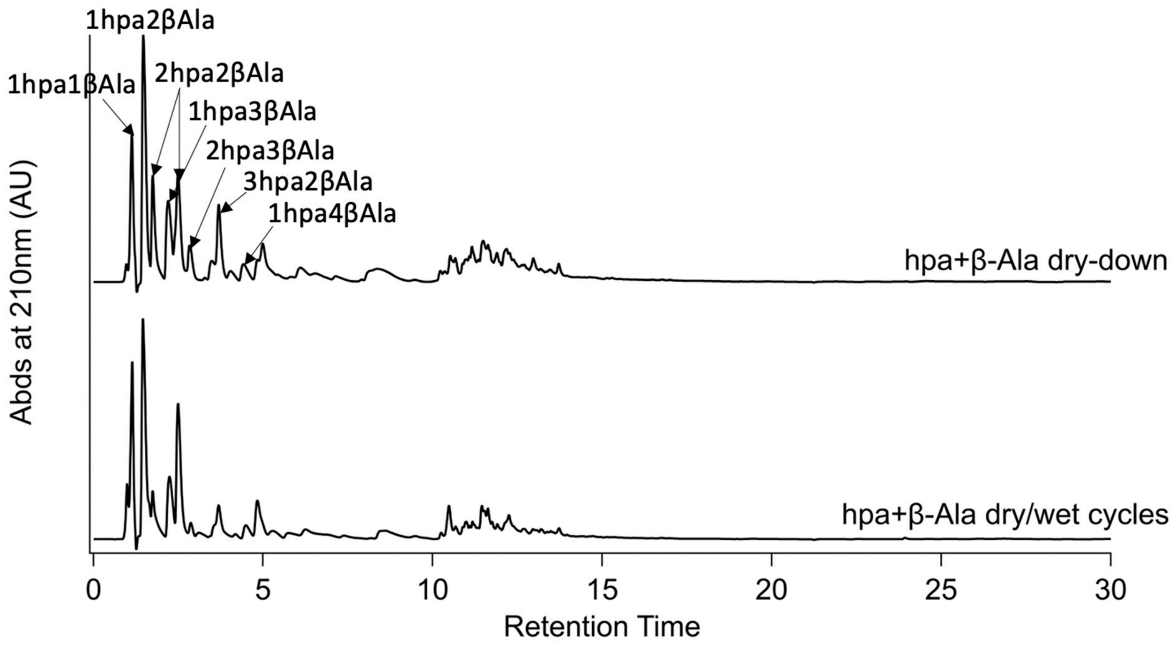

Figure S50. C18-HPLC analysis verifies the formation of depsipeptides following single-step dry-down reactions and dry/wet cycling reactions of 3-hydroxypropanoic acid (hpa) and beta-alanine (β-Ala). A mixture of **hpa** and **β-Ala** was subjected to single-step dry-down at 85 ˚C or to daily dry/wet cycling (16 hr dry/8 hr wet) for seven days. The resulting depsipeptides were analyzed by hydrophobicity-based separation using C18-HPLC and the peaks were identified by LC-MS. The presence of various peaks verifies the heterogenous nature of the product mixture and provides a relative abundance of the various polymers.

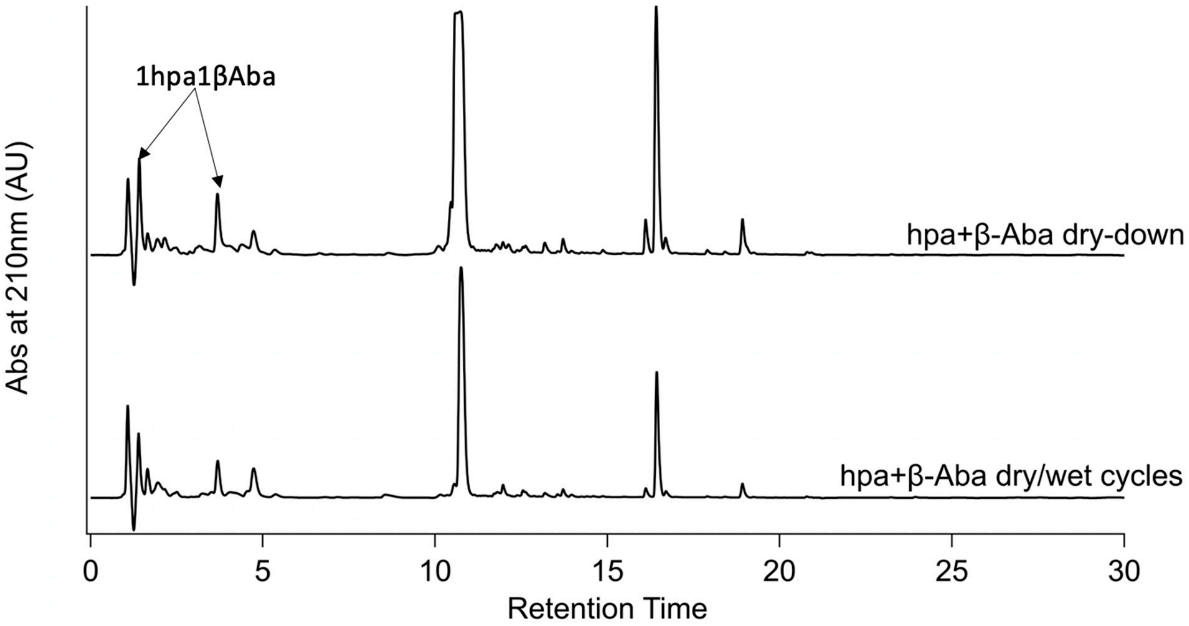

Figure S51. C18-HPLC analysis verifies the formation of depsipeptides following single-step dry-down reactions and dry/wet cycling reactions of 3-hydroxypropanoic acid (hpa) and beta-aminobutyric acid (β-Aba). A mixture of **hpa** and **β-Aba** was subjected to single-step dry-down at 85 °C or to daily dry/wet cycling (16 hr dry/8 hr wet) for seven days. The resulting depsipeptides were analyzed by hydrophobicity-based separation using C18-HPLC and the peaks were identified by LC-MS. The presence of various peaks verifies the heterogenous nature of the product mixture and provides a relative abundance of the various polymers.

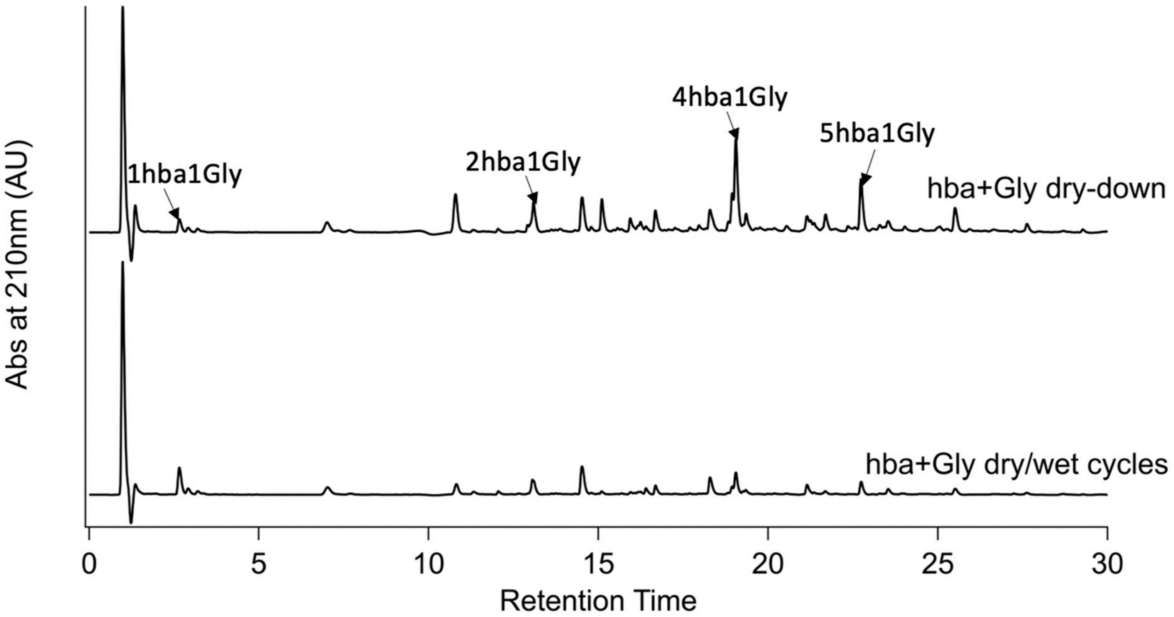

Figure S52. C18-HPLC analysis verifies the formation of depsipeptides following single-step dry-down reactions and dry/wet cycling reactions of 3-hydroxybutanoic acid (hba) and glycine (Gly). A mixture of **hba** and **Gly** was subjected to single-step dry-down at 85 ˚C or to daily dry/wet cycling (16 hr dry/8 hr wet) for seven days. The resulting depsipeptides were analyzed by hydrophobicity-based separation using C18-HPLC and the peaks were identified by LC-MS. The presence of various peaks verifies the heterogenous nature of the product mixture and provides a relative abundance of the various polymers.

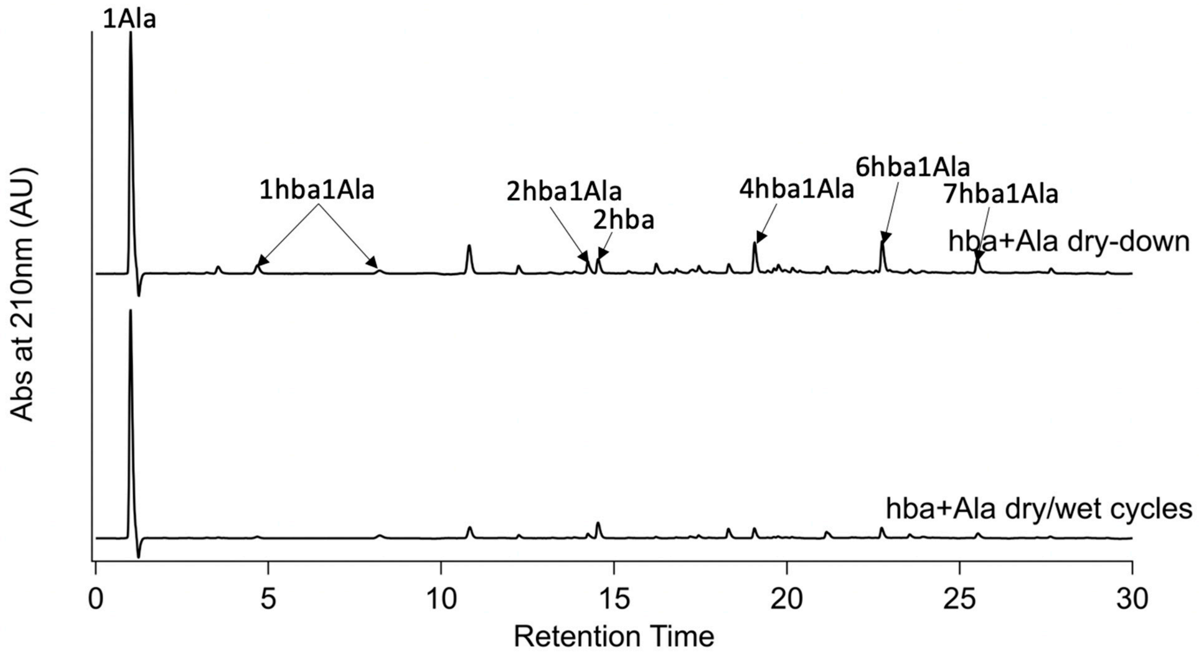

Figure S53. C18-HPLC analysis verifies the formation of depsipeptides following single-step dry-down reactions and dry/wet cycling reactions of 3-hydroxybutanoic acid (hba) and alanine (Ala). A mixture of **hba** and **Ala** was subjected to single-step dry-down at 85 ˚C or to daily dry/wet cycling (16 hr dry/8 hr wet) for seven days. The resulting depsipeptides were analyzed by hydrophobicity-based separation using C18-HPLC and the peaks were identified by LC-MS. The presence of various peaks verifies the heterogenous nature of the product mixture and provides a relative abundance of the various polymers.

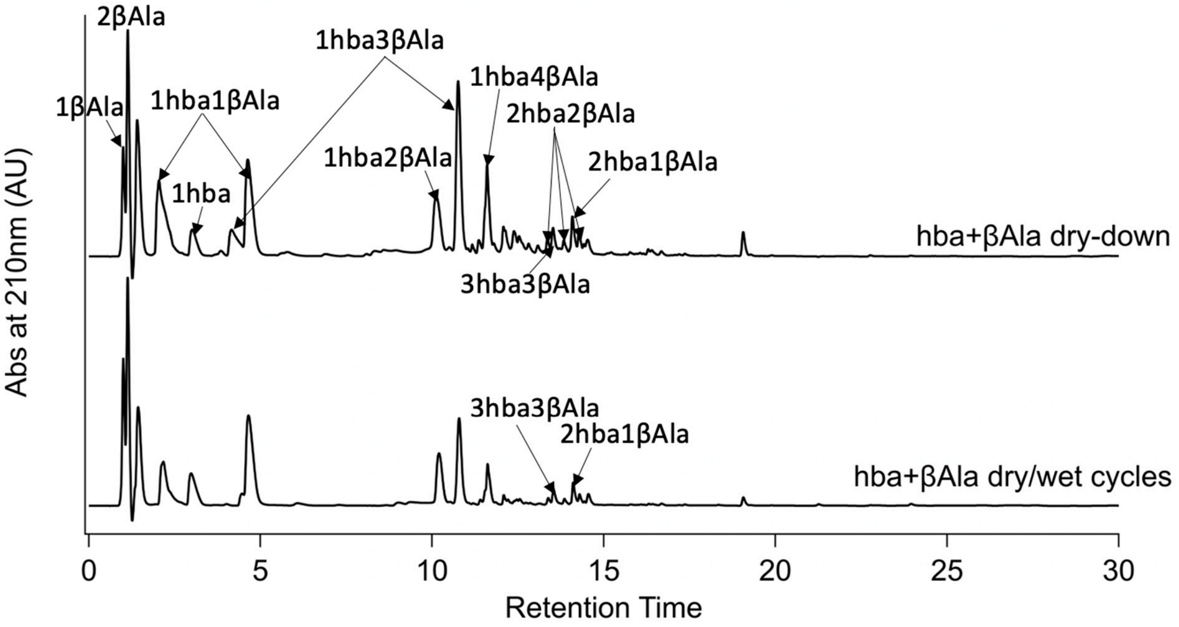

Figure S54. C18-HPLC analysis verifies the formation of depsipeptides following single-step dry-down reactions and dry/wet cycling reactions of 3-hydroxybutanoic acid (hba) and beta-alanine (β-Ala). A mixture of **hba** and **β-Ala** was subjected to single-step dry-down at 85 ˚C or to daily dry/wet cycling (16 hr dry/8 hr wet) for seven days. The resulting depsipeptides were analyzed by hydrophobicity-based separation using C18-HPLC and the peaks were identified by LC-MS. The presence of various peaks

verifies the heterogenous nature of the product mixture and provides a relative abundance of the various polymers.

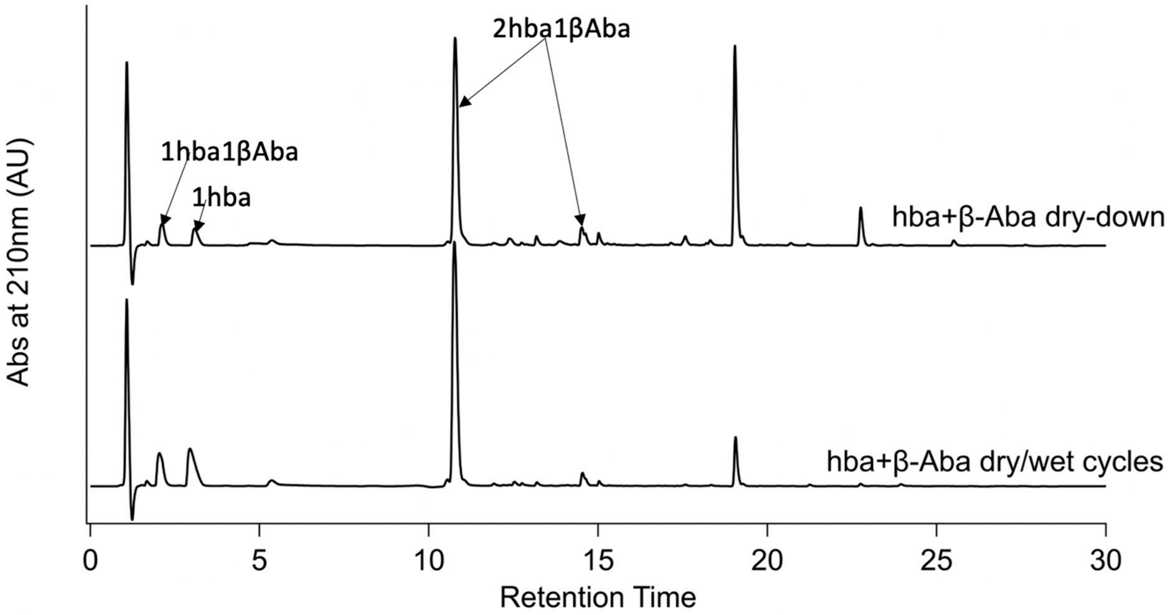

Figure S55. C18-HPLC analysis verifies the formation of depsipeptides following single-step dry-down reactions and dry/wet cycling reactions of 3-hydroxybutanoic

acid (hba) and beta-aminobutyric acid (β-Aba). A mixture of **hba** and **β-Aba** was subjected to single-step dry-down at 85 ˚C or to daily dry/wet cycling (16 hr dry/8 hr wet) for seven days. The resulting depsipeptides were analyzed by hydrophobicity-based separation using C18-HPLC and the peaks were identified by LC-MS. The presence of various peaks verifies the heterogenous nature of the product mixture and provides a relative abundance of the various polymers.

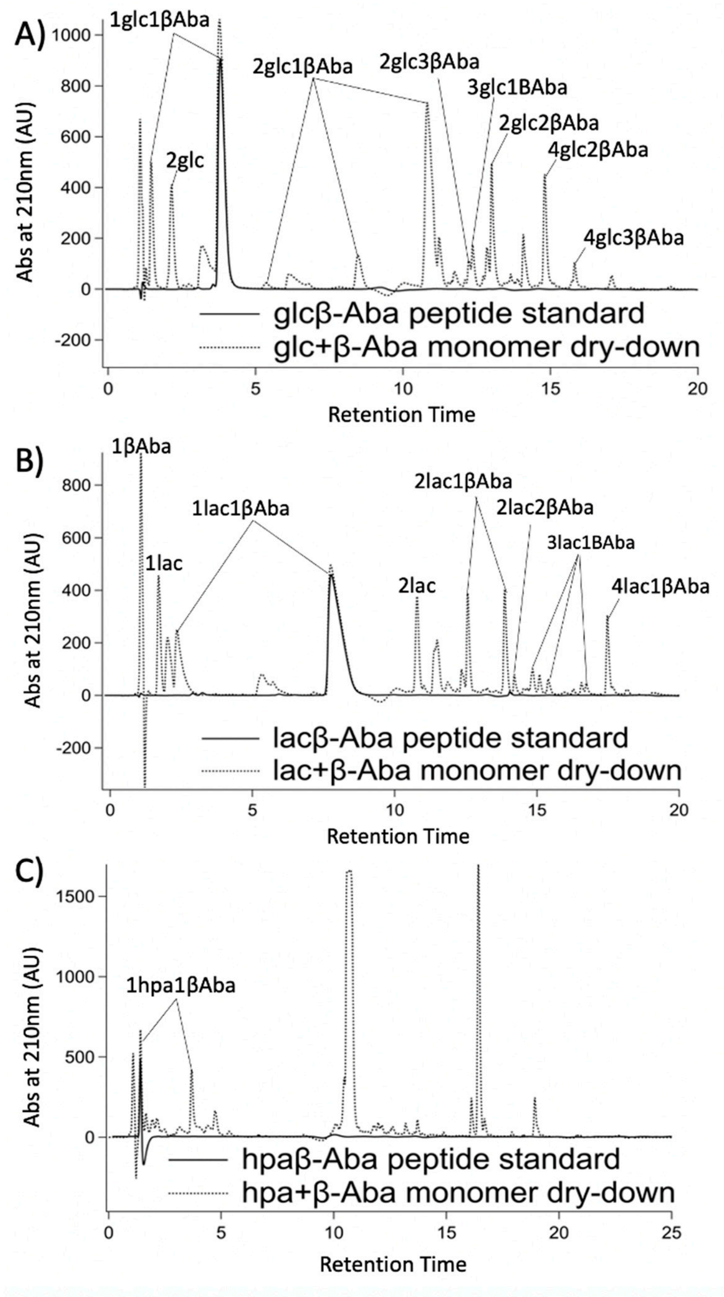

Figure S56. C18-HPLC analysis of single-step dry-down reactions of beta-aminobutyric acid (β-Aba) overlaid with authentic standards. glc, **lac**, or **hpa** were dried with **β-Aba** at 85 ˚C for seven days and the resulting HPLC data (dashed lines) were overlaid with pure peptide dimers (black lines).

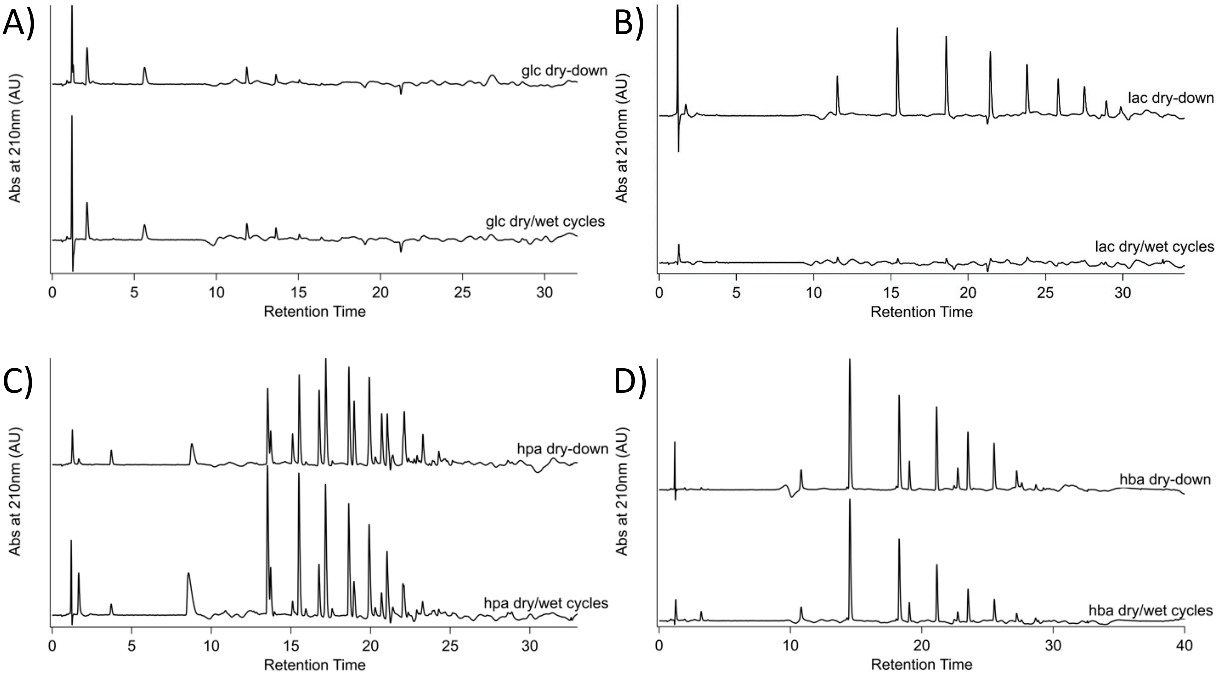

Figure S57. C18-HPLC analysis of control single-step dry-down and dry/wet cycling reactions of the hydroxy acids in the absence of amino acids. glycolic acid (**glc**, **A**), lactic acid (**lac**, **B**), 3-hydroxypropanoic acid (**hpa**, **C**), and 3-hydroxybutanoic acid (**hba**, **D**) were dried at 85˚C or subjected to daily dry/wet cycling (18 hr dry/6 hr wet) for seven days and analyzed by a C18-HPLC column.

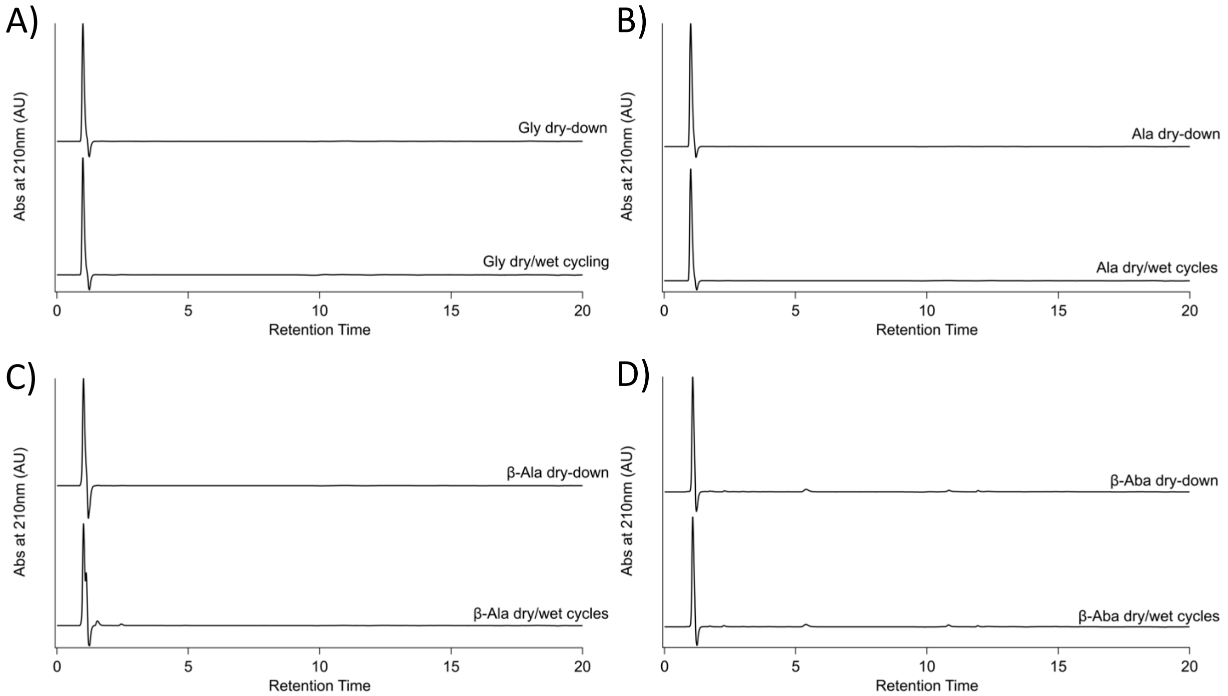

Figure S58. C18-HPLC analysis of control single-step dry-down reactions of the amino acids in the absence of hydroxy acids. Glycine (**Gly**, **A**), Alanine (**Ala**, **B**), β-alanine (**β-Ala**, **C**), and β-aminobutyric acid (**β-Aba**, **D**) were dried or subjected to daily dry/wet cycling (18 hr dry/6 hr wet) at 85˚C for seven days and analyzed by a C18-HPLC column.

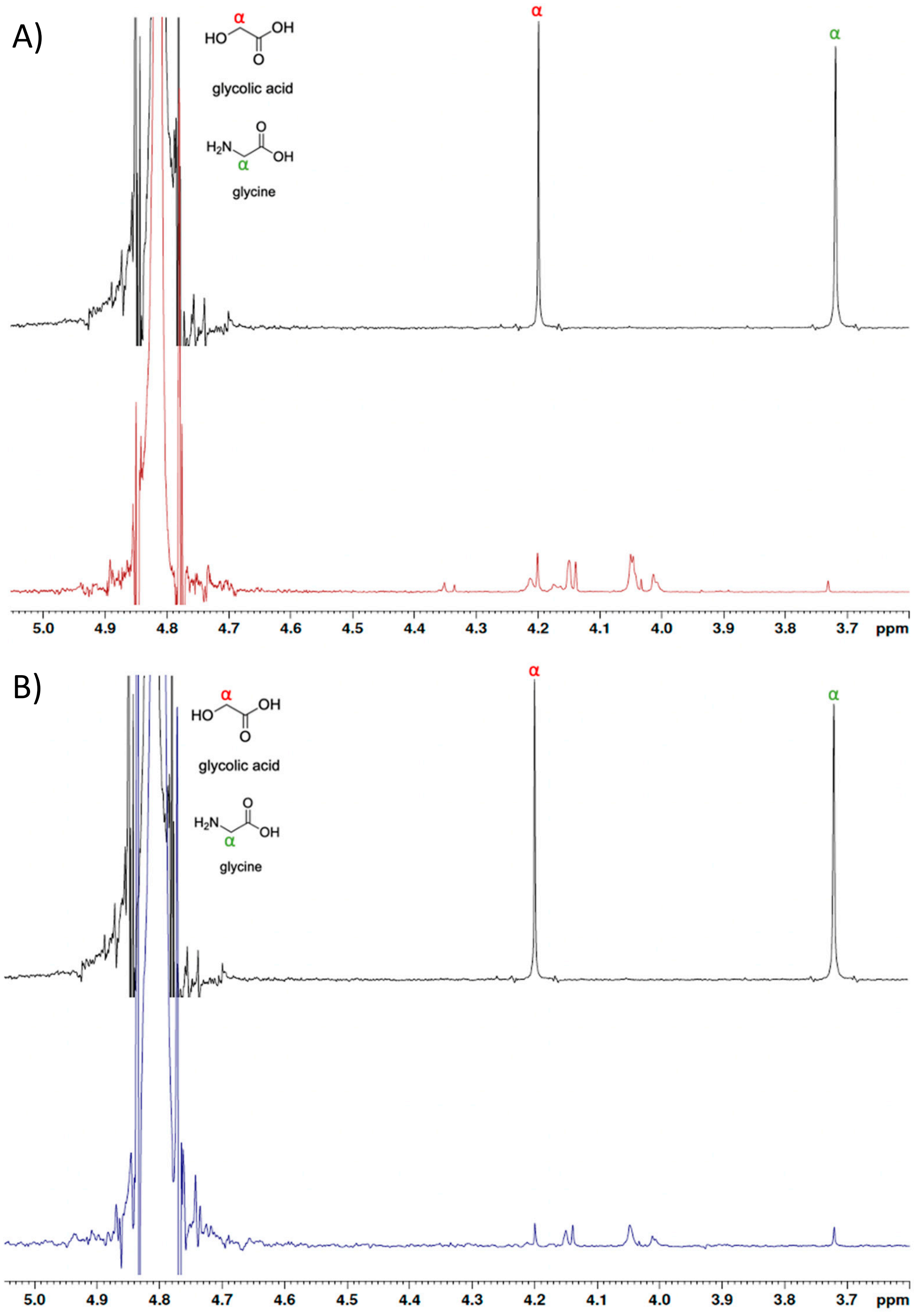

Figure S59. ¹H NMR spectra exhibit the extent of depsipeptide formation upon (A) single-step dry-down reactions and (B) dry/wet cycling of glycolic acid (glc) and glycine (Gly). ¹H NMR spectra of a mixture of **glc** and **Gly** in D_2O before (black) and after single-step dry-down reactions at 85 ˚C for seven days (red) show a 97.9% conversion of **Gly** into depsipeptide. In comparison, spectra after daily dry/wet cycling of 16 hr dry/8

hr wet for seven days (blue) show a conversion of 89. 6% of **Gly**. α-protons of **glc** are labeled in red; α-protons of **Gly** are labeled in green.

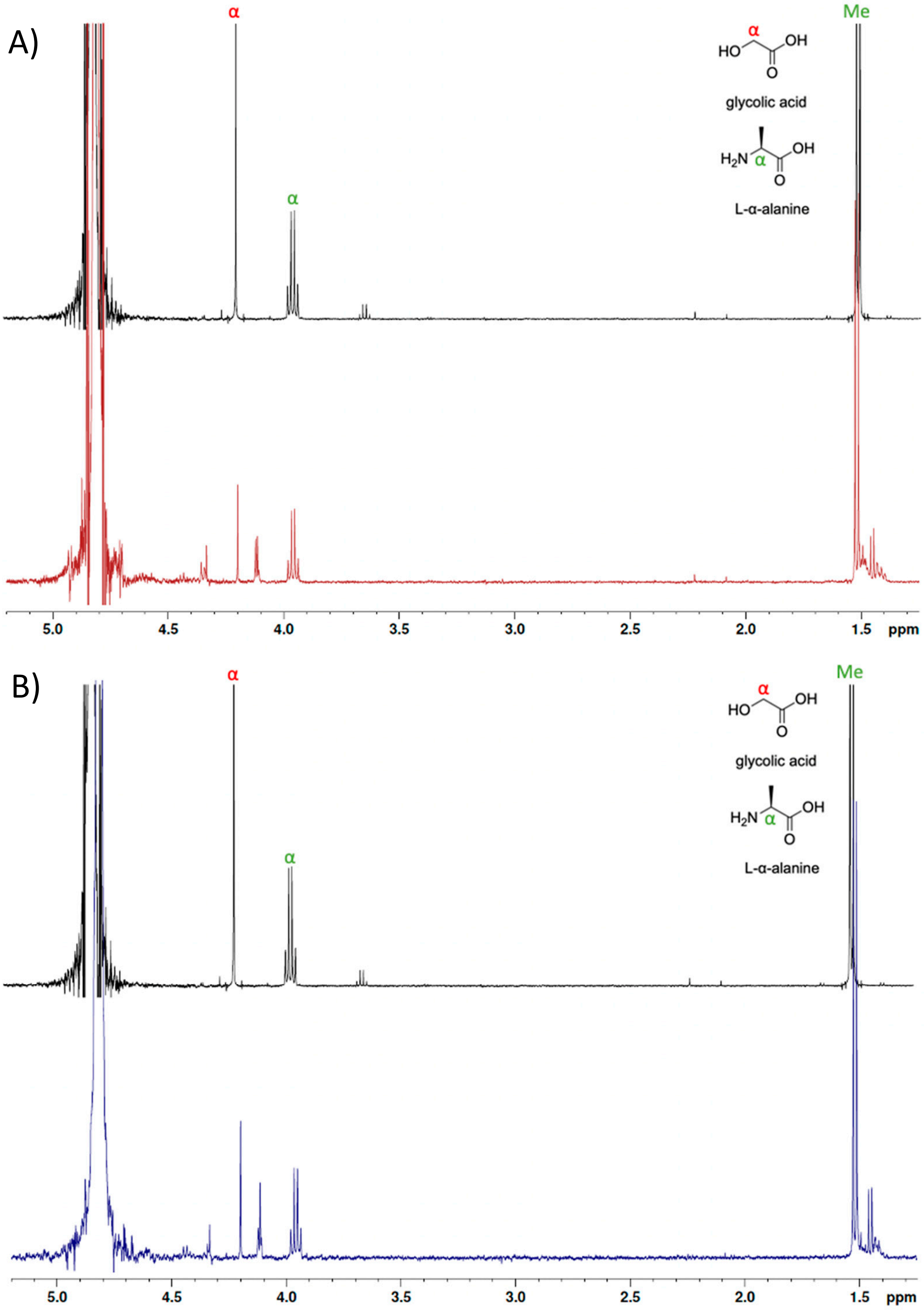

Figure S60. ^{1}H NMR spectra exhibit the extent of depsipeptide formation upon (a) single-step dry-down reactions and (b) dry/wet cycling of glycolic acid (glc) and alanine (Ala). ^{1}H NMR spectra of a mixture of **glc** and **Ala** in D_2O before (black) and after single-step dry-down reactions at 85 ˚C for seven days (red) show a 40.1% conversion of

Ala into depsipeptide. In comparison, spectra after daily dry/wet cycling of 16 hr dry/8 hr wet for seven days (blue) show a conversion of 40.3%. α-protons of **glc** are labeled in red; α-proton of **Ala** are labeled in green.

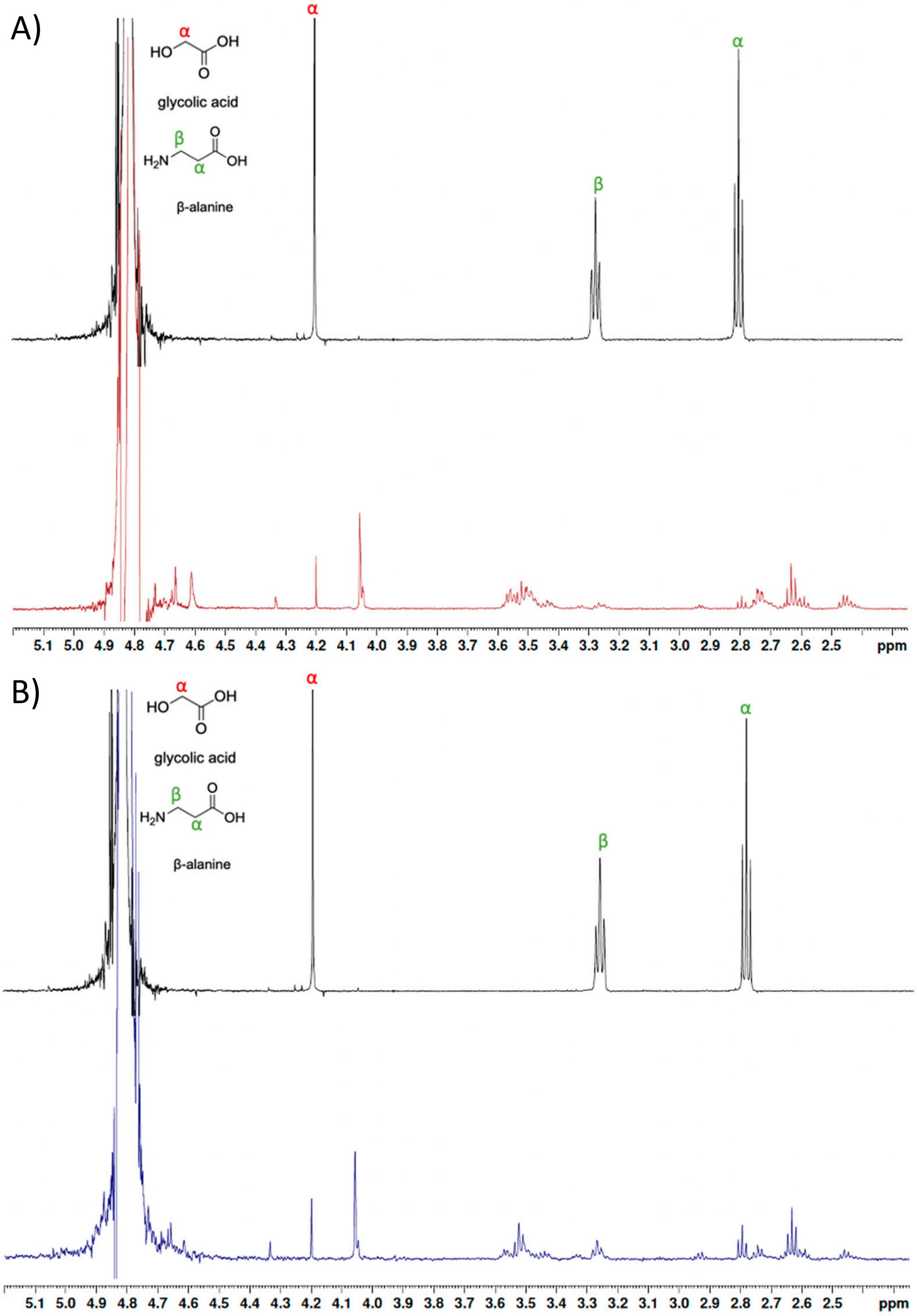

Figure S61. ^{1}H NMR spectra exhibit the extent of depsipeptide formation upon (a) single-step dry-down reactions and (b) dry/wet cycling of glycolic acid (glc) and beta-alanine (β-Ala). ^{1}H NMR spectra of a mixture of **glc** and **β-Ala** in D_2O before (black) and

after single-step dry-down reactions at 85 ˚C for seven days (red) show a 95.6% conversion of **β-Ala** into depsipeptide. In comparison, spectra after daily dry/wet cycling of 16 hr dry/8 hr wet for seven days (blue) show a conversion of 88.4%. α-protons of **glc** are labeled in red; protons of **β-Ala** are labeled in green.

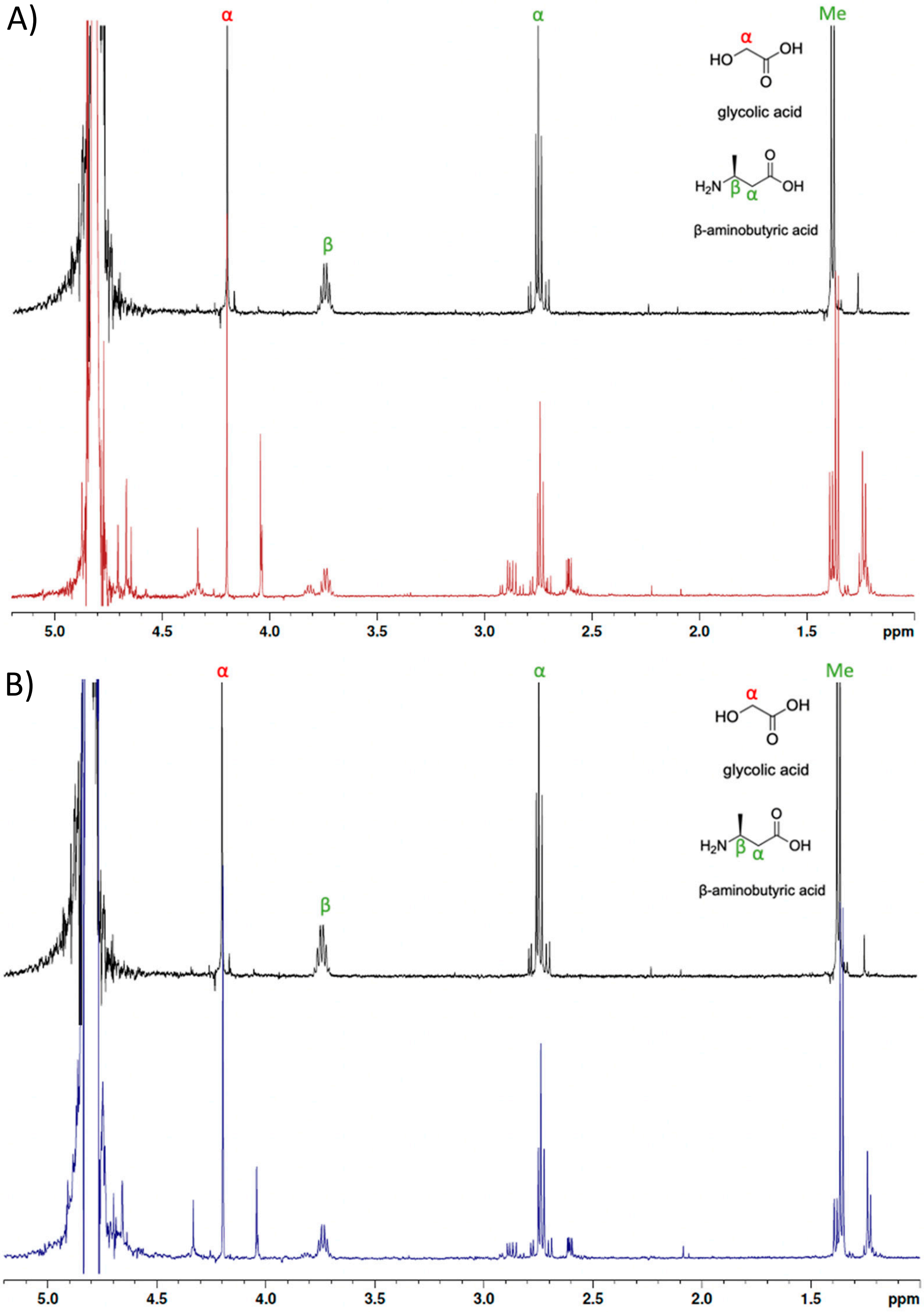

Figure S62. ^{1}H NMR spectra exhibit the extent of depsipeptide formation upon (a) single-step dry-down reactions and (b) dry/wet cycling of glycolic acid (glc) and beta-

aminobutyric acid (β-Aba). ^1H NMR spectra of a mixture of **glc** and **β-Aba** in D_2O before (black) and after single-step dry-down reactions at 85 ˚C for seven days (red) show a 59.7% conversion of **β-Aba** into depsipeptide. In comparison, spectra after daily dry/wet cycling of 16 hr dry/8 hr wet for seven days (blue) show a conversion of 43.8%. α-protons of **glc** are labeled in red; protons of **β-Aba** are labeled in green.

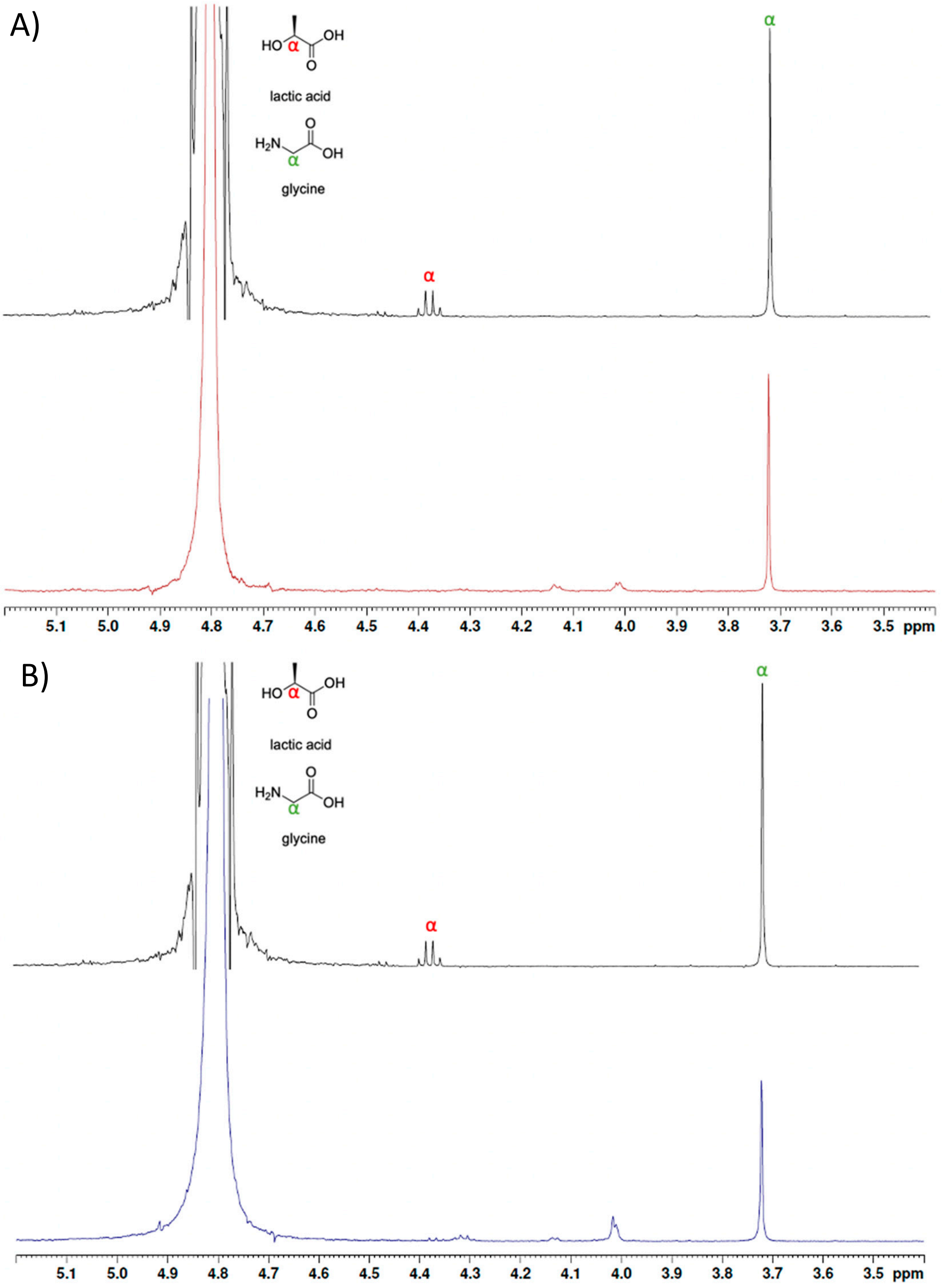

Figure S63. ^{1}H NMR spectra exhibit the extent of depsipeptide formation upon (a) single-step dry-down reactions and (b) dry/wet cycling of lactic acid (lac) and glycine (Gly). ^{1}H NMR spectra of a mixture of **lac** and **β-Ala** in D_2O before (black) and after single-step dry-down reactions at 85 ˚C for seven days (red) show a 34.7% conversion of **Gly** into depsipeptide. In comparison, spectra after daily dry/wet cycling of 16 hr dry/8 hr wet for seven days (blue) show a conversion of 43.8%. α-protons of **lac** are labeled in red; protons of **β-Ala** are labeled in green.

A)

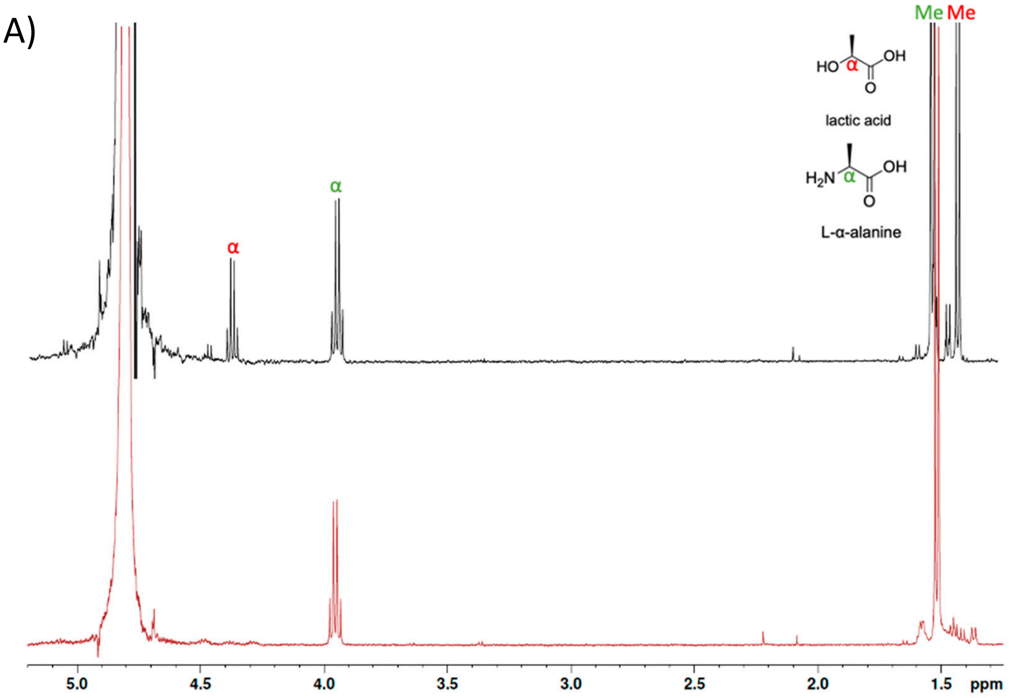

B)

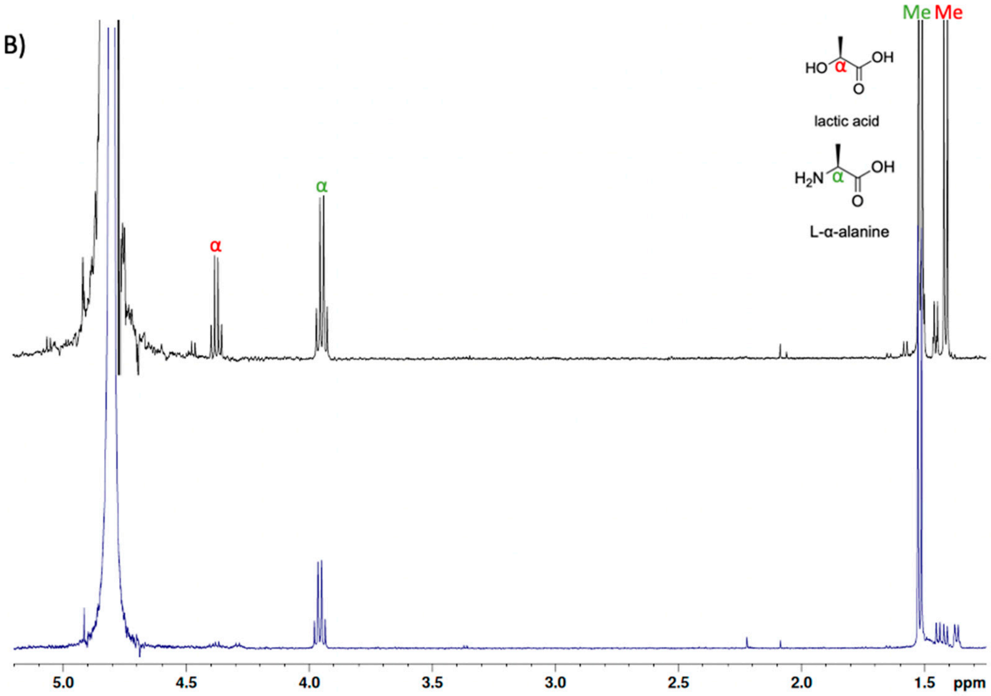

Figure S64. ^{1}**H NMR spectra exhibit the extent of depsipeptide formation upon (a) single-step dry-down reactions and (b) dry/wet cycling of lactic acid (lac) and alanine (Ala).** ^{1}H NMR spectra of a mixture of **lac** and **Ala** in D_2O before (black) and after single-step dry-down reactions at 85 ˚C for seven days (red) show a 13.2% conversion of **Ala** into depsipeptide. In comparison, spectra after daily dry/wet cycling of 16 hr dry/8 hr wet for seven days (blue) show a conversion of 41.4%. α-protons of **lac** are labeled in red; α-proton of **Ala** is labeled in green.

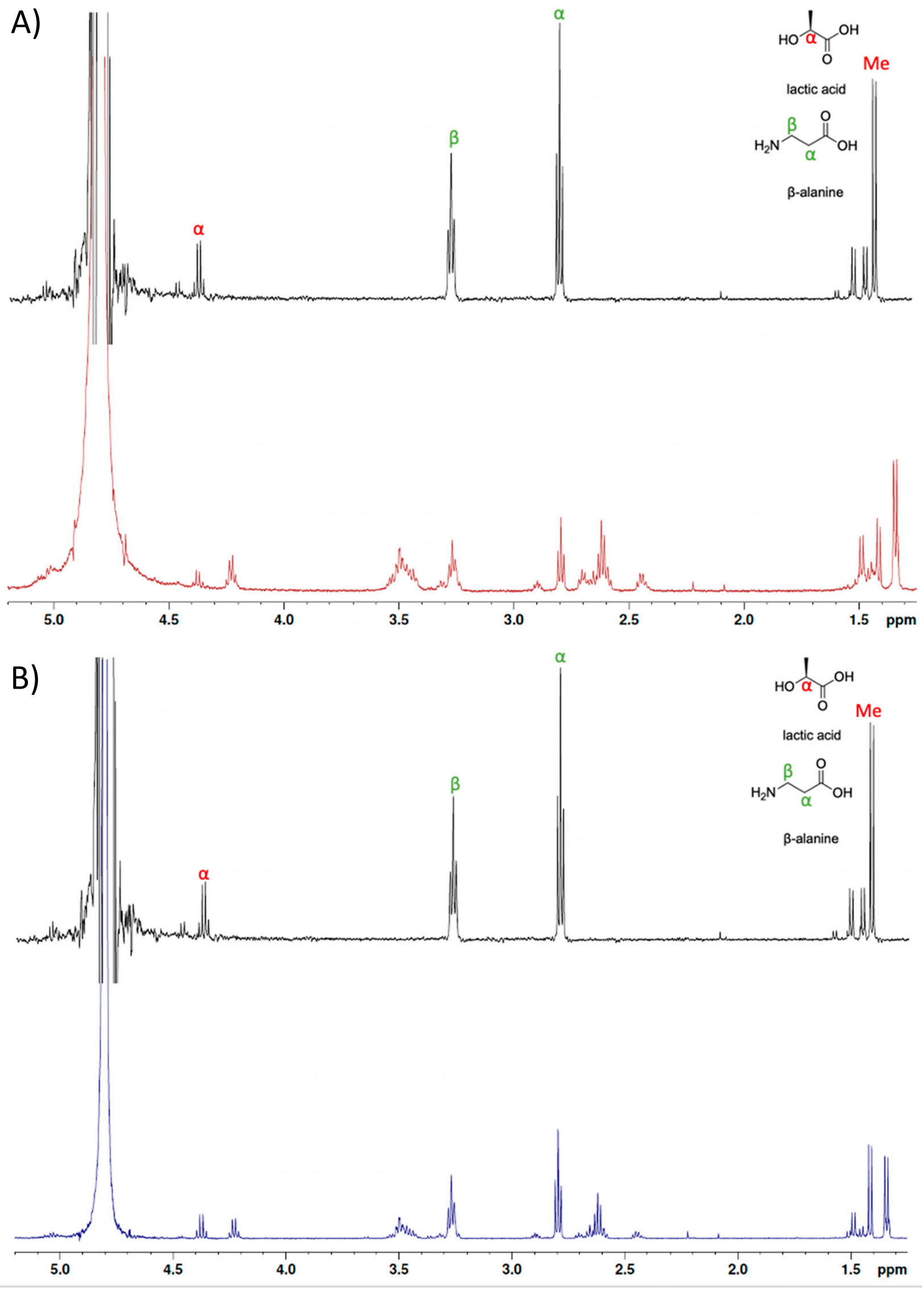

Figure S65. ^{1}H NMR spectra exhibit the extent of depsipeptide formation upon (a) single-step dry-down reactions and (b) dry/wet cycling of lactic acid (lac) and beta-alanine (β-Ala). ^{1}H NMR spectra of a mixture of **lac** and **β-Ala** in D_2O before (black) and after single-step dry-down reactions at 85 ˚C for seven days (red) show a 77.2% conversion of **β-Ala** into depsipeptide. In comparison, spectra after daily dry/wet cycling

of 16 hr dry/8 hr wet for seven days (blue) show a conversion of 59.7%. α-protons of **lac** are labeled in red; protons of **β-Ala** are labeled in green.

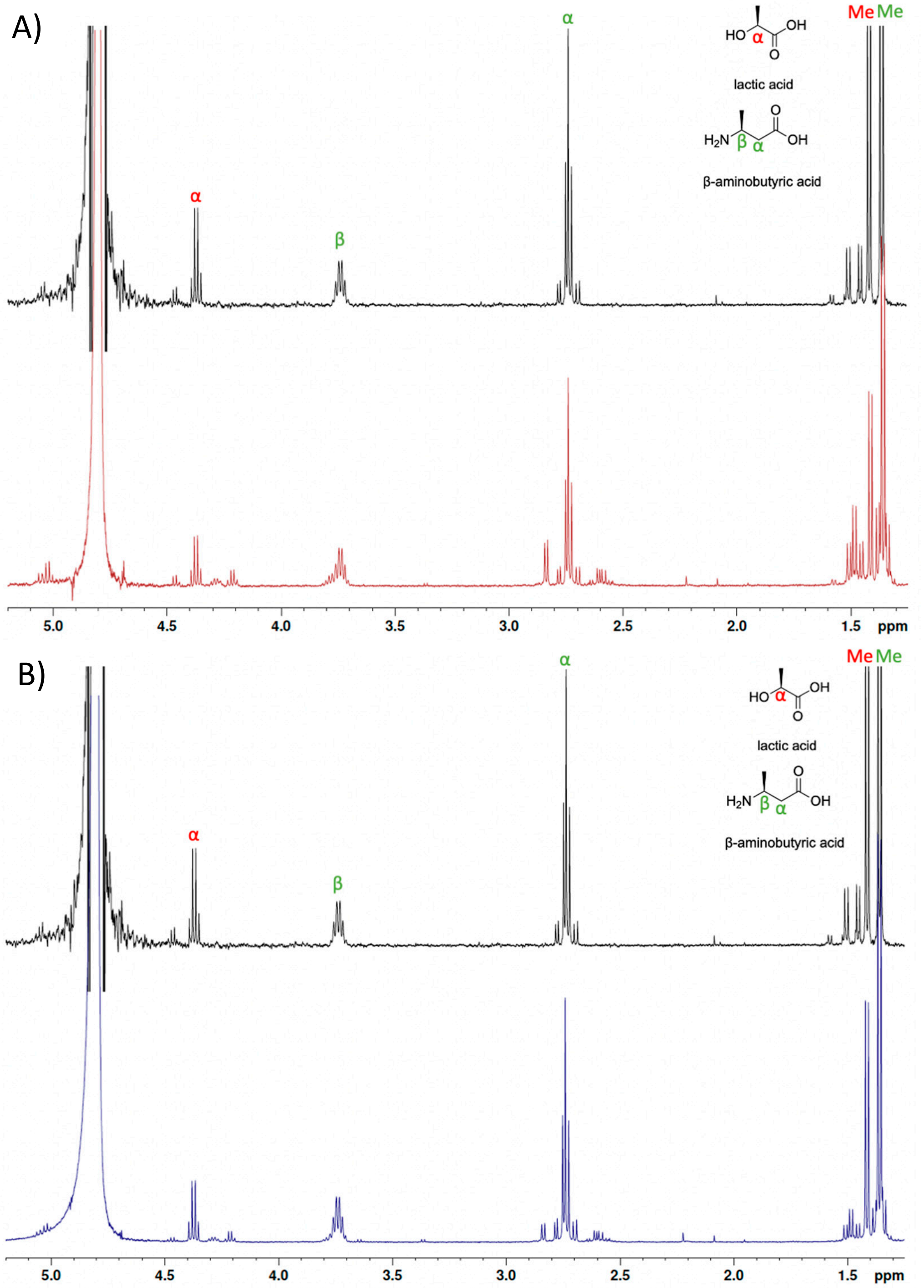

Figure S66. ^{1}H NMR spectra exhibit the extent of depsipeptide formation upon (a) single-step dry-down reactions and (b) dry/wet cycling of lactic acid (lac) and beta-aminobutyric acid (β-Aba). ^{1}H NMR spectra of a mixture of **lac** and **β-Aba** in D_2O before (black) and after single-step dry-down reactions at 85 ˚C for seven days (red) show a

32.5% conversion of **β-Aba** into depsipeptide. In comparison, spectra after daily dry/wet cycling of 16 hr dry/8 hr wet for seven days (blue) show a conversion of 19.1%. α-protons of **lac** are labeled in red; protons of **β-Aba** are labeled in green.

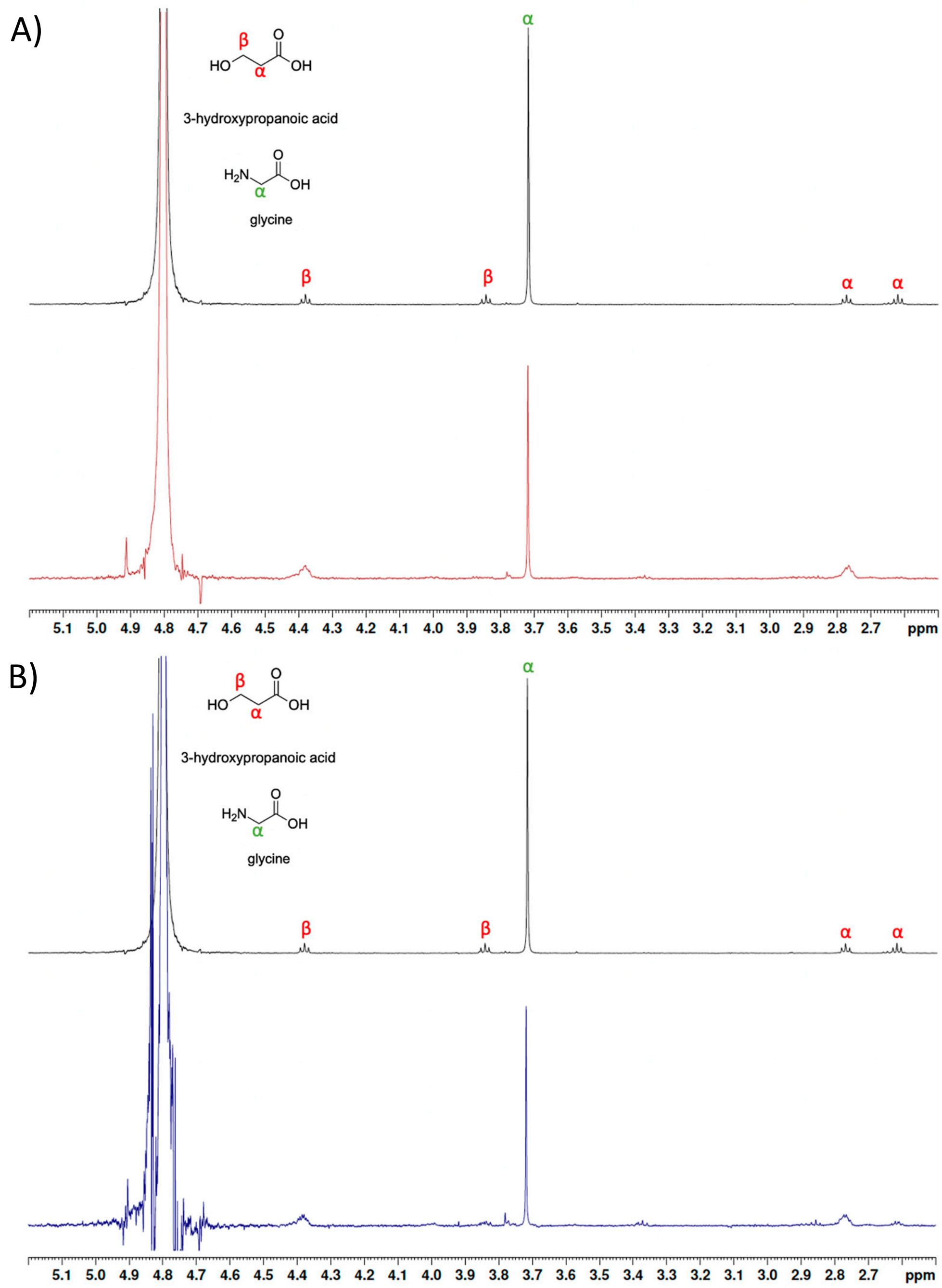

Figure S67. ^{1}H NMR spectra exhibit the extent of depsipeptide formation upon (a) single-step dry-down reactions and (b) dry/wet cycling of 3-hydroxypropanoic acid (hpa) and glycine (Gly). ^{1}H NMR spectra of a mixture of **hpa** and **Gly** in D_2O before (black) and after single-step dry-down reactions at 85 ˚C for seven days (red) show a

28.8% conversion of **Gly** into depsipeptide. In comparison, spectra after daily dry/wet cycling of 16 hr dry/8 hr wet for seven days (blue) show a conversion of 29.9%. Protons of **hpa** are labeled in red; α-protons of **Gly** are labeled in green.

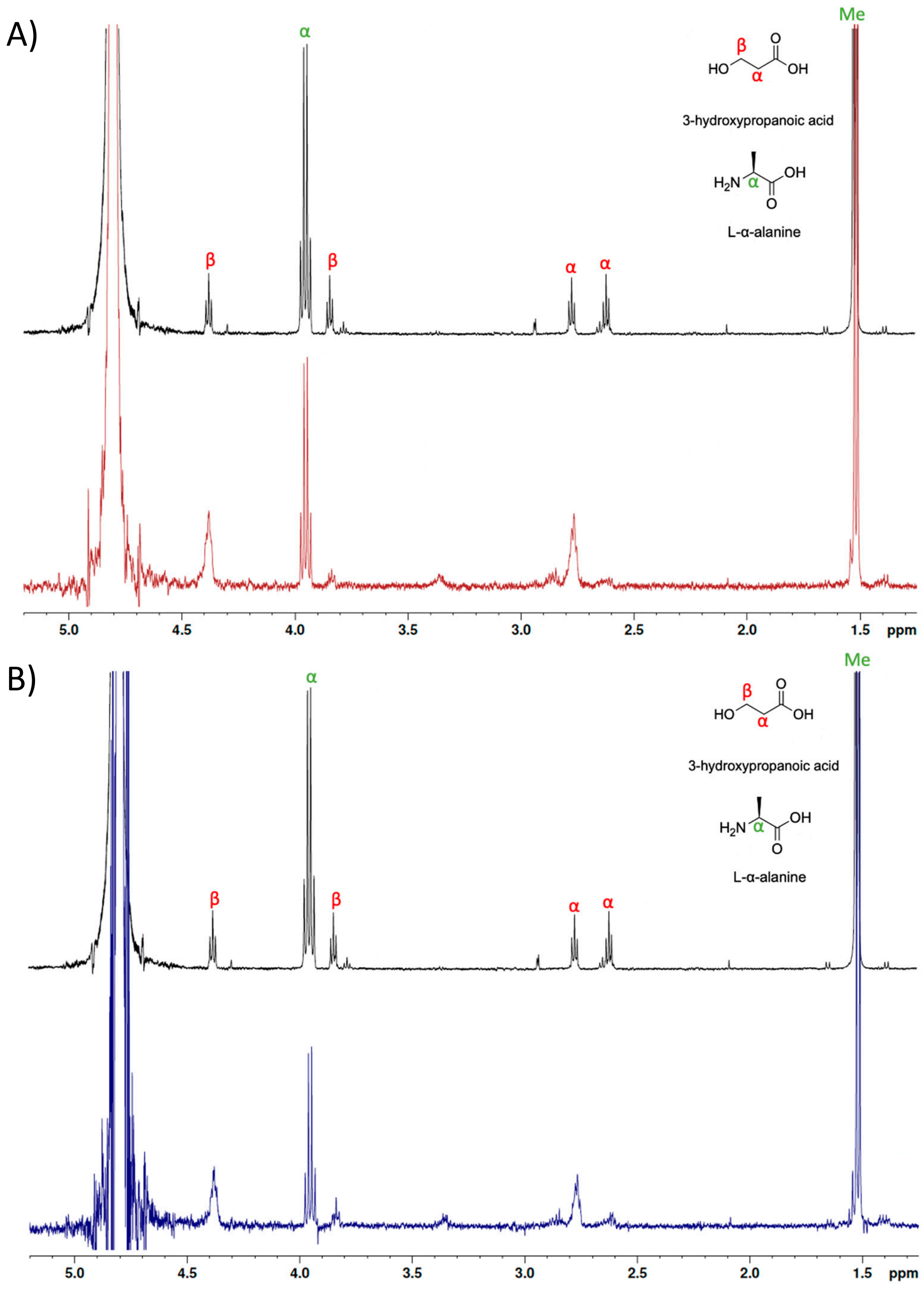

Figure S68. ¹H NMR spectra exhibit the extent of depsipeptide formation upon (a) single-step dry-down reactions and (b) dry/wet cycling of 3-hydroxypropanoic acid (hpa) and alanine (Ala). ¹H NMR spectra of a mixture of **hpa** and **Ala** in D_2O before (black) and after single-step dry-down reactions at 85 ˚C for seven days (red) show a

13.1% conversion of **Ala** into depsipeptide. In comparison, spectra after daily dry/wet cycling of 16 hr dry/8 hr wet for seven days (blue) show a conversion of 28.2%. Protons of **hpa** are labeled in red; α-proton of **Ala** is labeled in green.

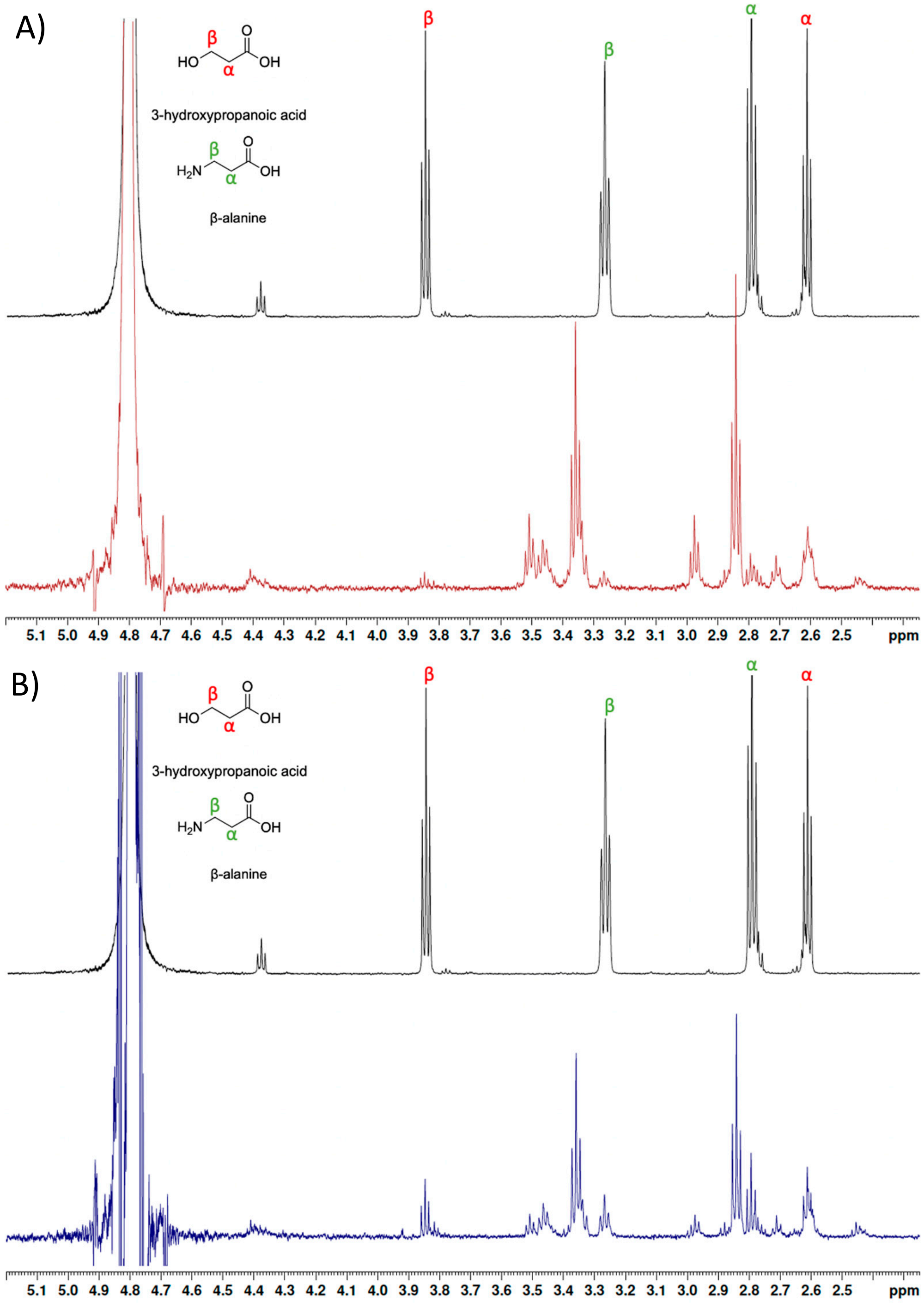

Figure S69. ^{1}H NMR spectra exhibit the extent of depsipeptide formation upon (a) single-step dry-down reactions and (b) dry/wet cycling of 3-hydroxypropanoic acid (hpa) and beta-alanine (β-Ala). ^{1}H NMR spectra of a mixture of **hpa** and **β-Ala** in D_2O before (black) and after single-step dry-down reactions at 85 ˚C for seven days (red) show a 94.1% conversion of **β-Ala** into depsipeptide. In comparison, spectra after daily dry/wet cycling of 16 hr dry/8 hr wet for seven days (blue) show a conversion of 79.1%. Protons of **hpa** are labeled in red; protons of **β-Ala** are labeled in green.

A)

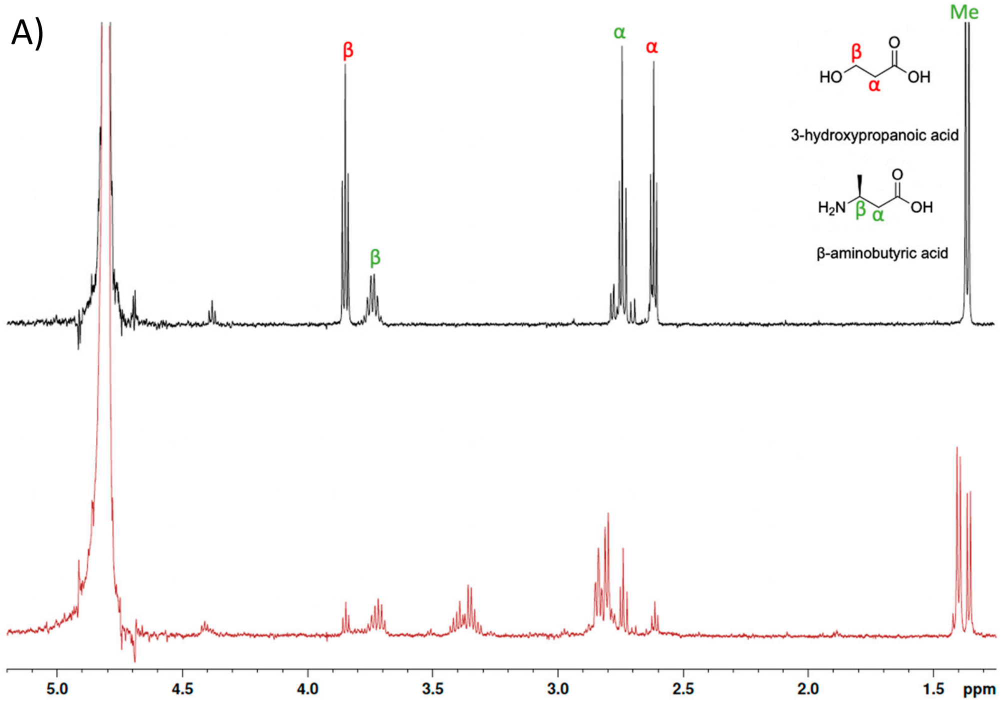

B)

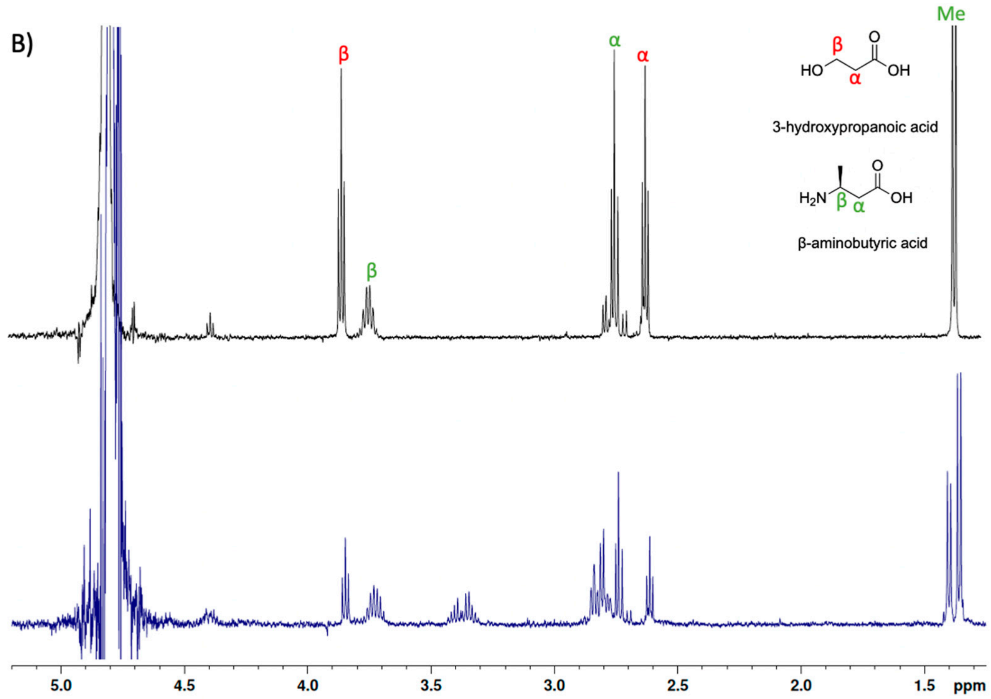

Figure S70. ^{1}H NMR spectra exhibit the extent of depsipeptide formation upon (a) single-step dry-down reactions and (b) dry/wet cycling of 3-hydroxypropanoic acid (hpa) and beta-aminobutyric acid (β-Aba). ^{1}H NMR spectra of a mixture of **hpa** and **β-Aba** in D_2O before (black) and after single-step dry-down reactions at 85 ˚C for seven days (red) show a 72.3% conversion of **β-Aba** into depsipeptide. In comparison, spectra after daily dry/wet cycling of 16 hr dry/8 hr wet for seven days (blue) show a conversion of 62.9%. Protons of **hpa** are labeled in red; protons of **β-Aba** are labeled in green.

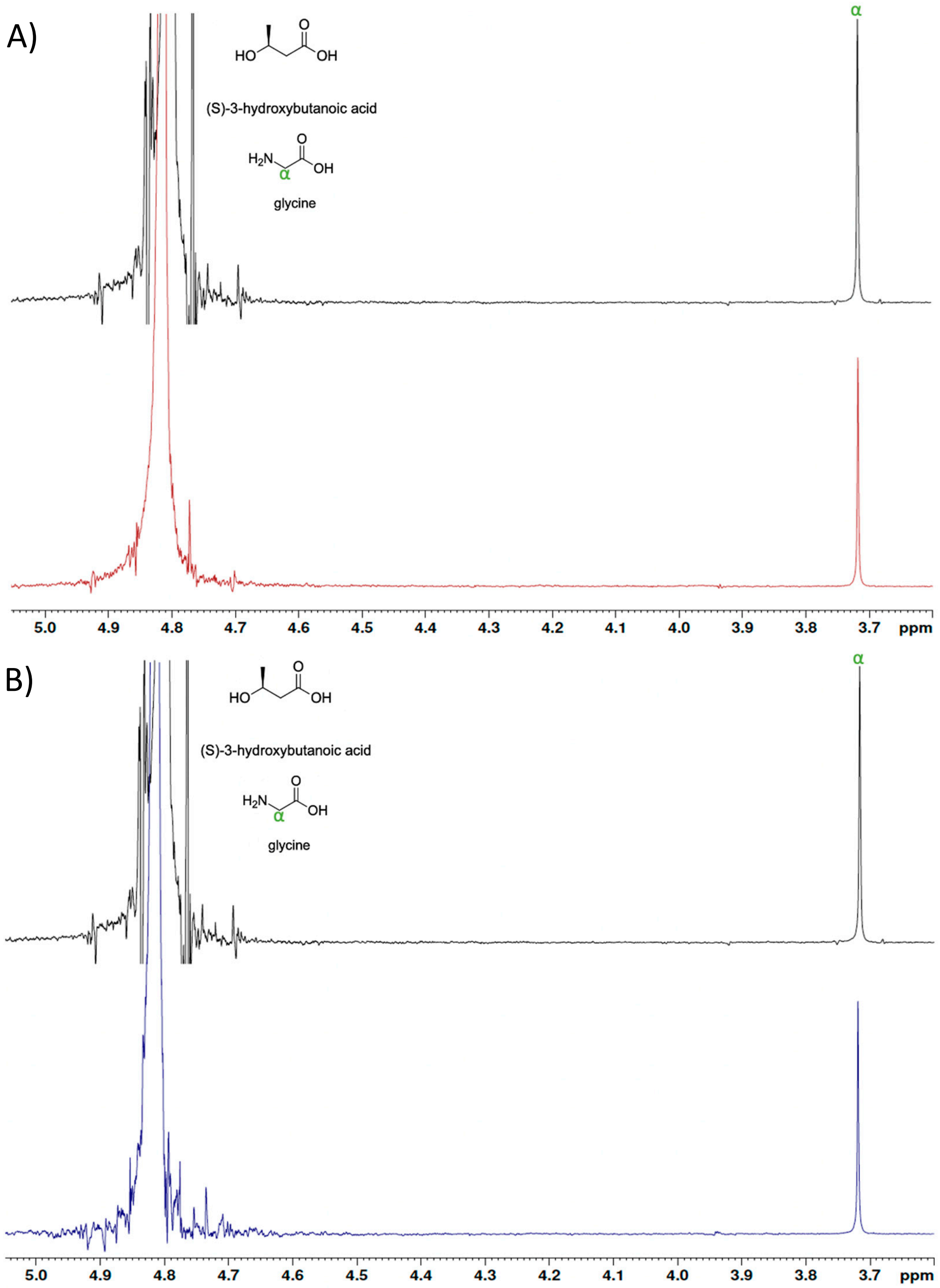

Figure S71. ^{1}H NMR spectra exhibit the extent of depsipeptide formation upon (a) single-step dry-down reactions and (b) dry/wet cycling of 3-hydroxybutanoic acid (hba) and glycine (Gly). ^{1}H NMR spectra of a mixture of **hba** and **Gly** in D_2O before (black) and after single-step dry-down reactions at 85 ˚C for seven days (red) show a 16.3% conversion of **Gly** into depsipeptide. In comparison, spectra after daily dry/wet cycling of 16 hr dry/8 hr wet for seven days (blue) show a conversion of 19.2%. α-protons

of **Gly** are labeled in green. **hba** was completely lost during lyophilization, which is carried out prior to the NMR analysis.

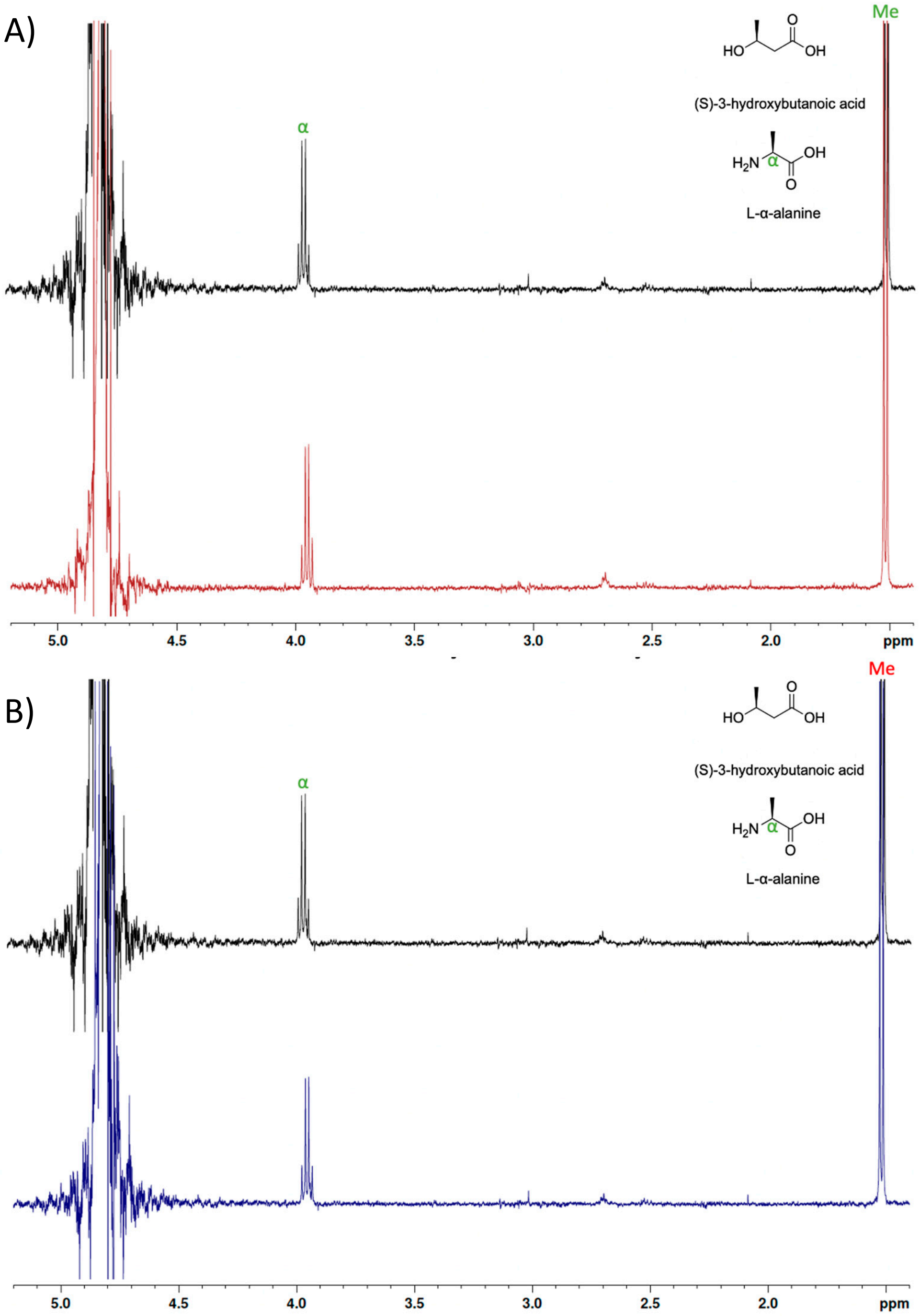

Figure S72. ^{1}H NMR spectra exhibit the extent of depsipeptide formation upon (a) single-step dry-down reactions and (b) dry/wet cycling of 3-hydroxybutanoic acid (hba) and alanine (Ala). ^{1}H NMR spectra of a mixture of **hba** and **Ala** in D_2O before (black) and after single-step dry-down reactions at 85 ˚C for seven days (red) show an 8.1% conversion of **Ala** into depsipeptide. In comparison, spectra after daily dry/wet

cycling of 16 hr dry/8 hr wet for seven days (blue) show a conversion of 29.5%. Protons of **hba** are labeled in red; α-proton of **Ala** is labeled in green. **hba** was completely lost during lyophilization, which is carried out prior to the NMR analysis.

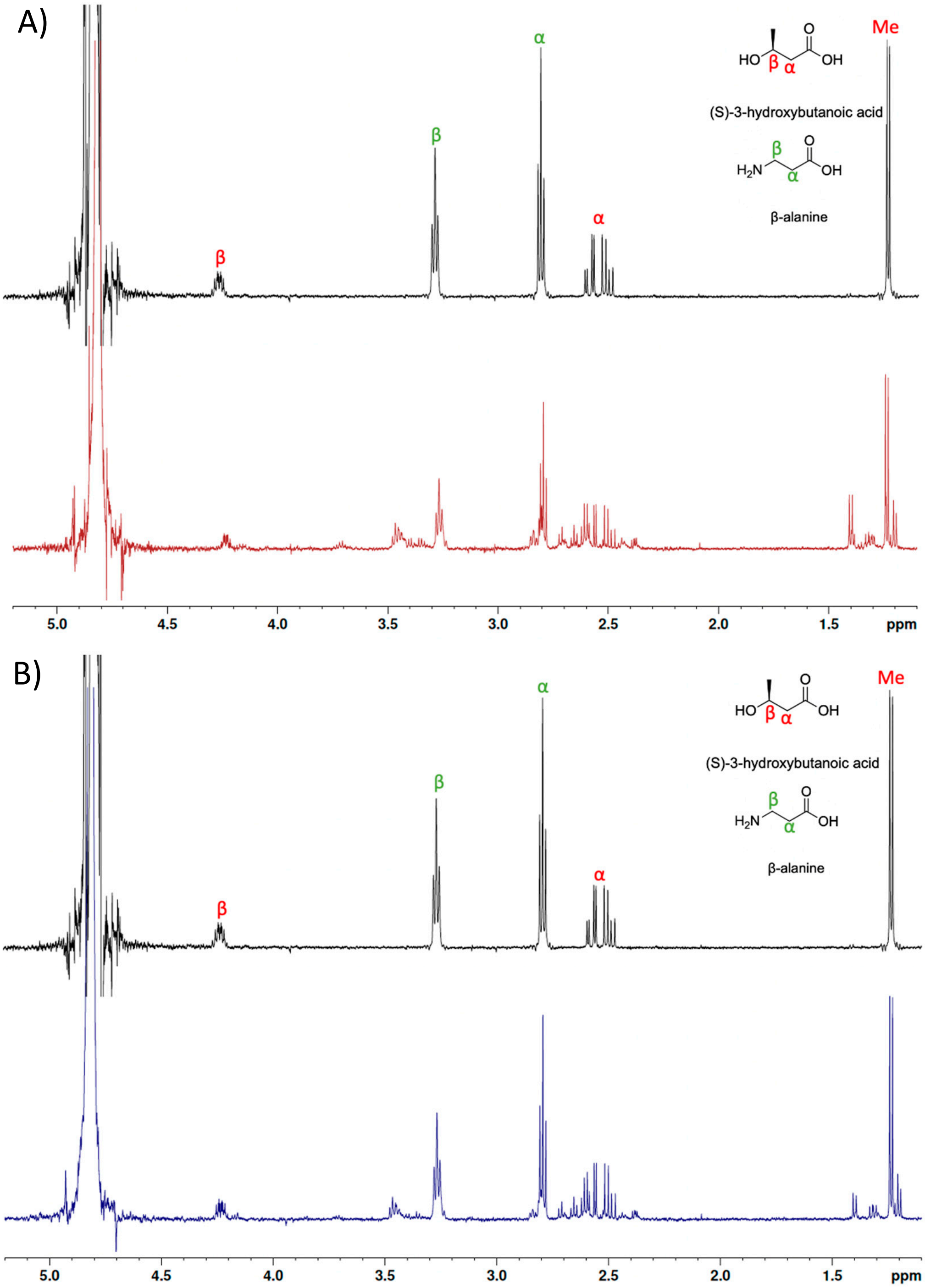

Figure S73. ^{1}H NMR spectra exhibit the extent of depsipeptide formation upon (a) single-step dry-down reactions and (b) dry/wet cycling of 3-hydroxybutanoic acid (hba) and beta-alanine (β-Ala). ^{1}H NMR spectra of a mixture of **hba** and **β-Ala** in D_2O

before (black) and after single-step dry-down reactions at 85 ˚C for seven days (red) show a 62.2% conversion of **β-Ala** into depsipeptide. In comparison, spectra after daily dry/wet cycling of 16 hr dry/8 hr wet for seven days (blue) show a conversion of 40.2%. Protons of **hba** are labeled in red; protons of **β-Ala** are labeled in green.

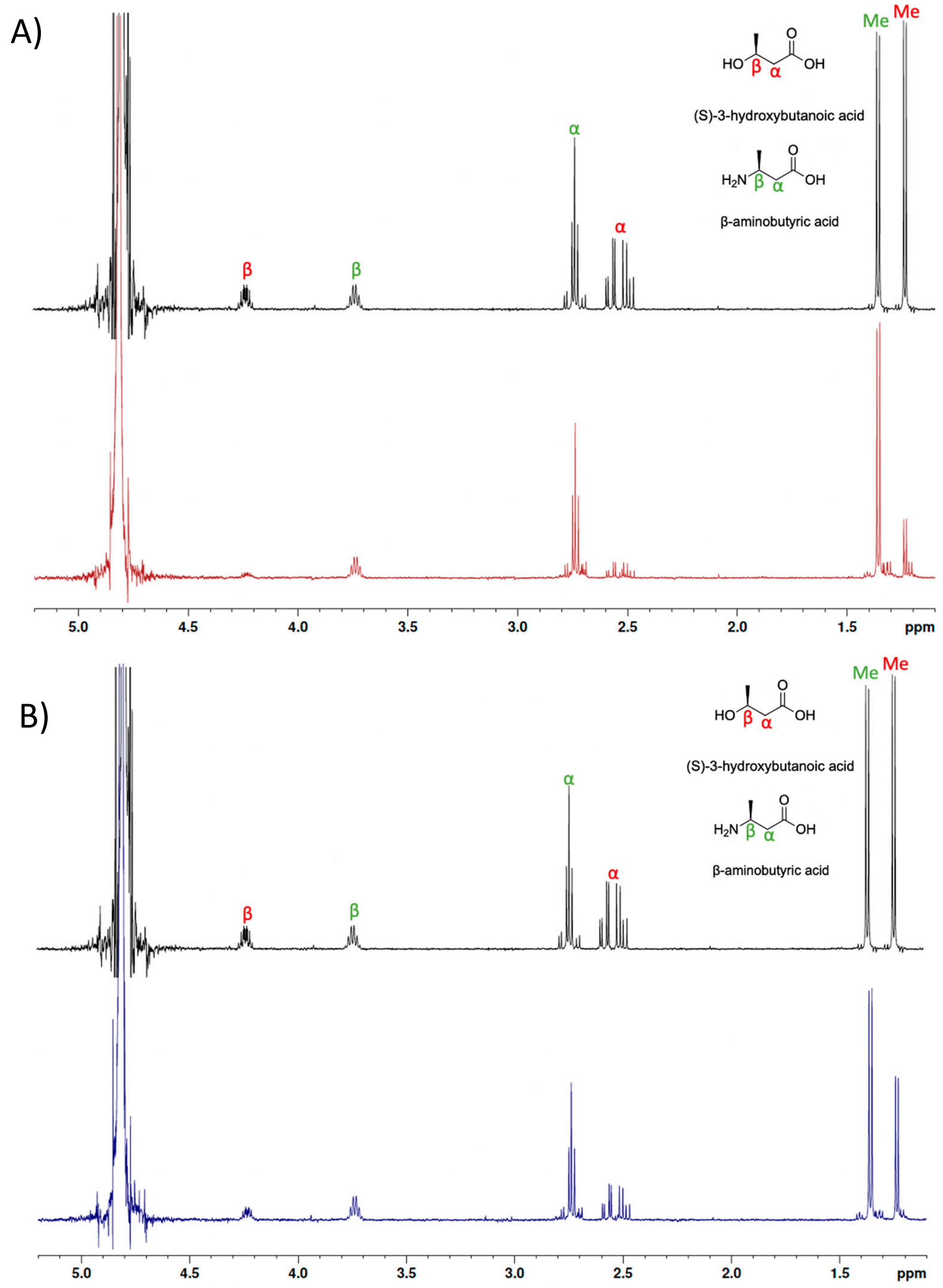

Figure S74. ^{1}H NMR spectra exhibit the extent of depsipeptide formation upon (a) single-step dry-down reactions and (b) dry/wet cycling of 3-hydroxybutanoic acid (hba) and beta-aminobutyric acid (β-Aba). ^{1}H NMR spectra of a mixture of **hba** and **β-Aba** in D_2O before (black) and after single-step dry-down reactions at 85 ˚C for seven

days (red) show a 3.8% conversion of **β-Aba** into depsipeptide. In comparison, spectra after daily dry/wet cycling of 16 hr dry/8 hr wet for seven days (blue) show a conversion of 11.2%. Protons of **hba** are labeled in red; protons of **β-Aba** are labeled in green.

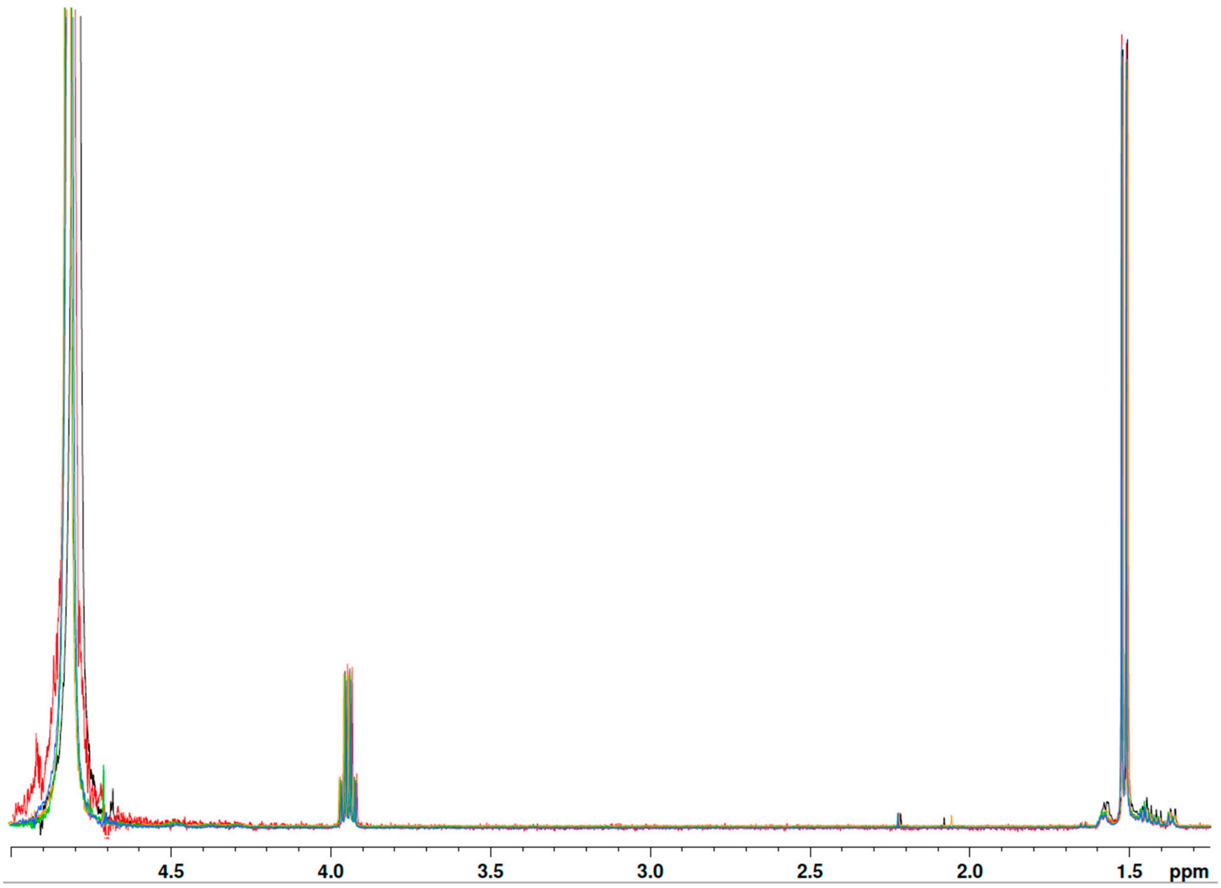

Figure S75. ^{1}H NMR spectra of replicates of lactic acid (lac) and alanine (Ala) single-step dry-down reactions confirms reproducibility of extent of amino acid conversion. ^{1}H NMR spectra of four independent replicates following single-step dry-down reactions of **lac** and **Ala** for seven days at 85˚C confirms reproducibility of observed percent conversion (standard deviation ±3.48 %).

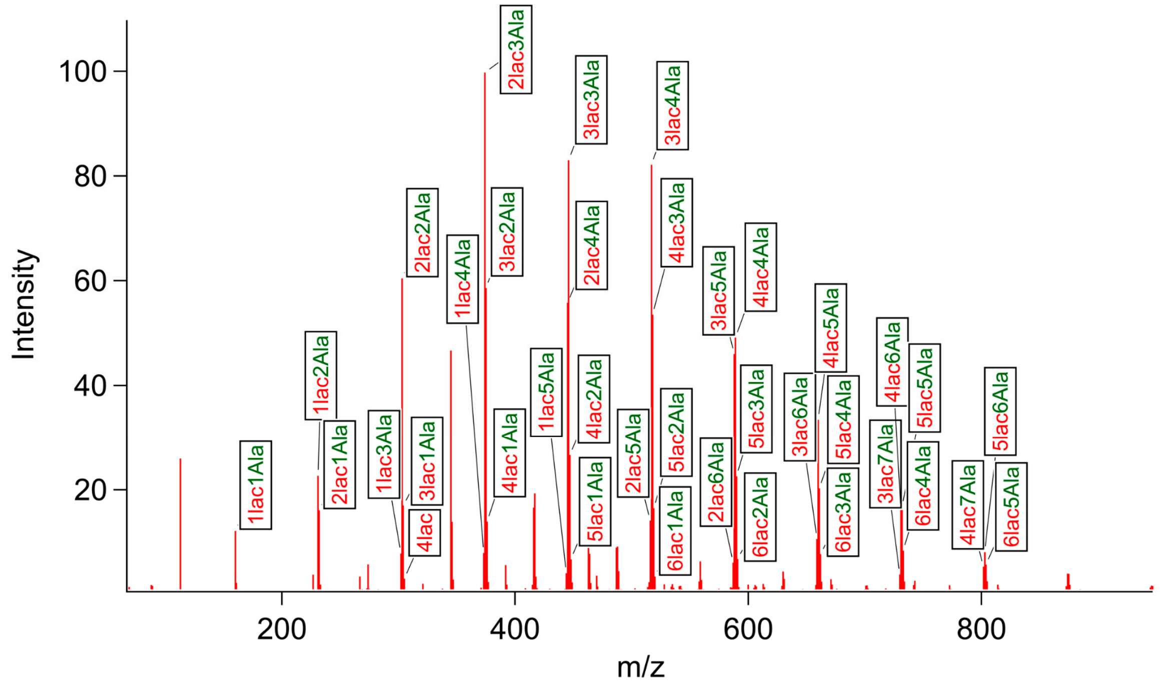

Figure S76. ESI-MS of the last cycle in the extended dry/wet cycling reaction of lactic acid (lac) and alanine (Ala) supports the formation of depsipeptides. lac and **Ala** were subjected to weekly dry/wet cycles (4 days dry/3 days wet) at 85 ˚C for 8 weeks and the resulting depsipeptides from the last cycle (C8) were analyzed by negative-mode ESI-MS, indicating a variety of depsipeptides. **lac** is labeled in red; **Ala** is labeled in green. All labeled species correspond to [M-H]$^-$ ions.

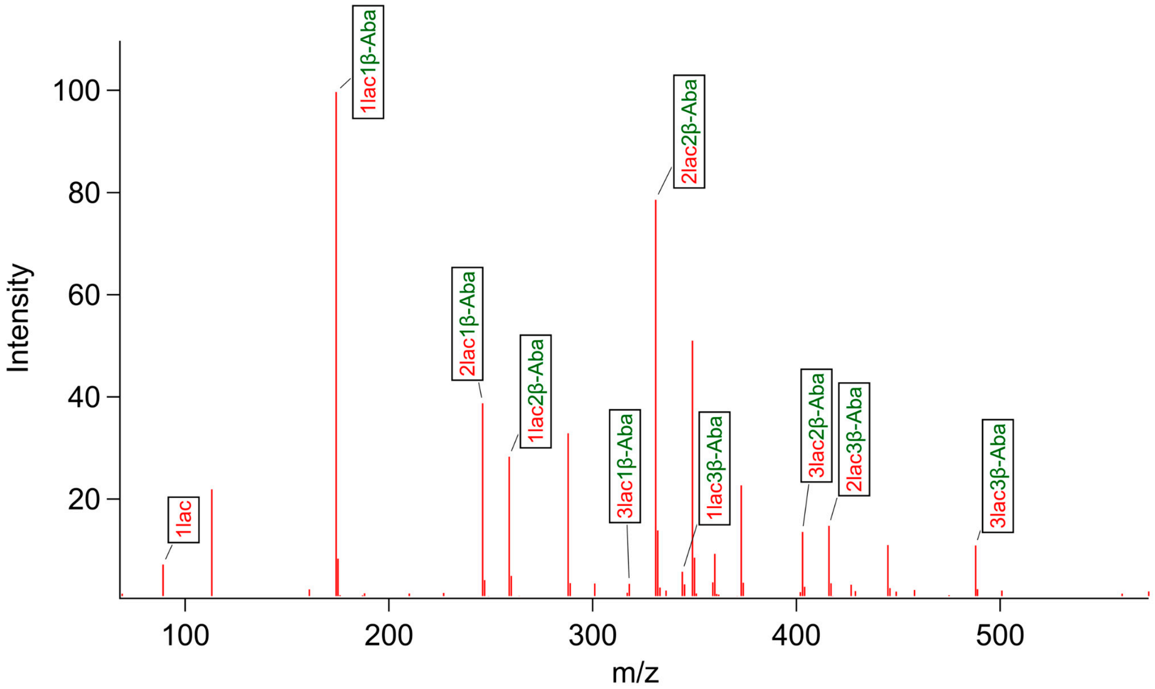

Figure S77. ESI-MS of the last cycle in the extended dry/wet cycling reaction of lactic acid (lac) and β-aminobutyric acid (β-Aba) supports the formation of depsipeptides. **lac** and **β-Aba** were subjected to weekly dry/wet cycles (4 days dry/3 days wet) at 85 ˚C for 8 weeks and the resulting depsipeptides from the last cycle (C8) were analyzed by negative-mode ESI-MS, indicating a variety of depsipeptides. **lac** is labeled in red; **β-Aba** is labeled in green. All labeled species correspond to $[M-H]^-$ ions.

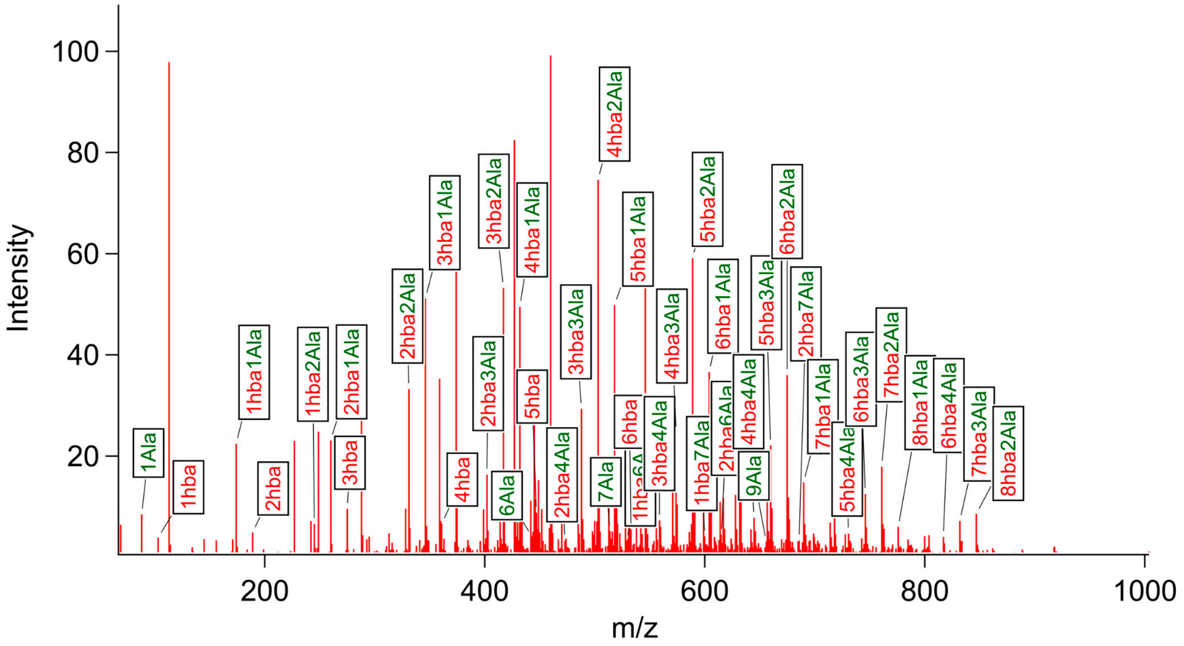

Figure S78. ESI-MS of the last cycle in the extended dry/wet cycling reaction of 3-hydroxybutanoic acid (hba) and alanine (Ala) supports the formation of depsipeptides. **hba** and **Ala** were subjected to weekly dry/wet cycles (4 days dry/3 days wet) at 85 °C for 8 weeks and the resulting depsipeptides from the last cycle (C8) were analyzed by negative-mode ESI-MS, indicating a variety of depsipeptides. **hba** is labeled in red; **Ala** is labeled in green. All labeled species correspond to $[M-H]^-$ ions.

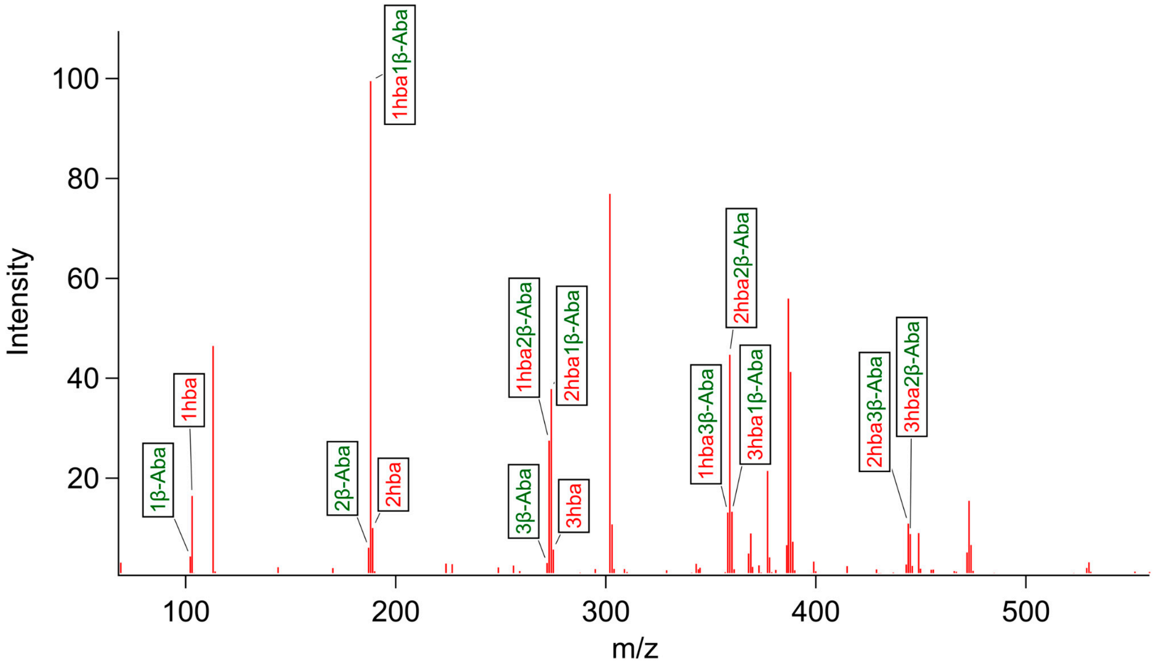

Figure S79. ESI-MS of the last cycle in the extended dry/wet cycling reaction of 3-hydroxybutanoic acid (hba) and β-aminobutyric acid (β-Aba) supports the formation of depsipeptides. hba and **β-Aba** were subjected to weekly dry/wet cycles (4 days dry/3 days wet) at 85 ˚C for 8 weeks and the resulting depsipeptides from the last cycle (C8) were analyzed by negative-mode ESI-MS, indicating a variety of depsipeptides. **hba** is labeled in red; **β-Aba** is labeled in green. All labeled species correspond to $[M-H]^-$ ions.

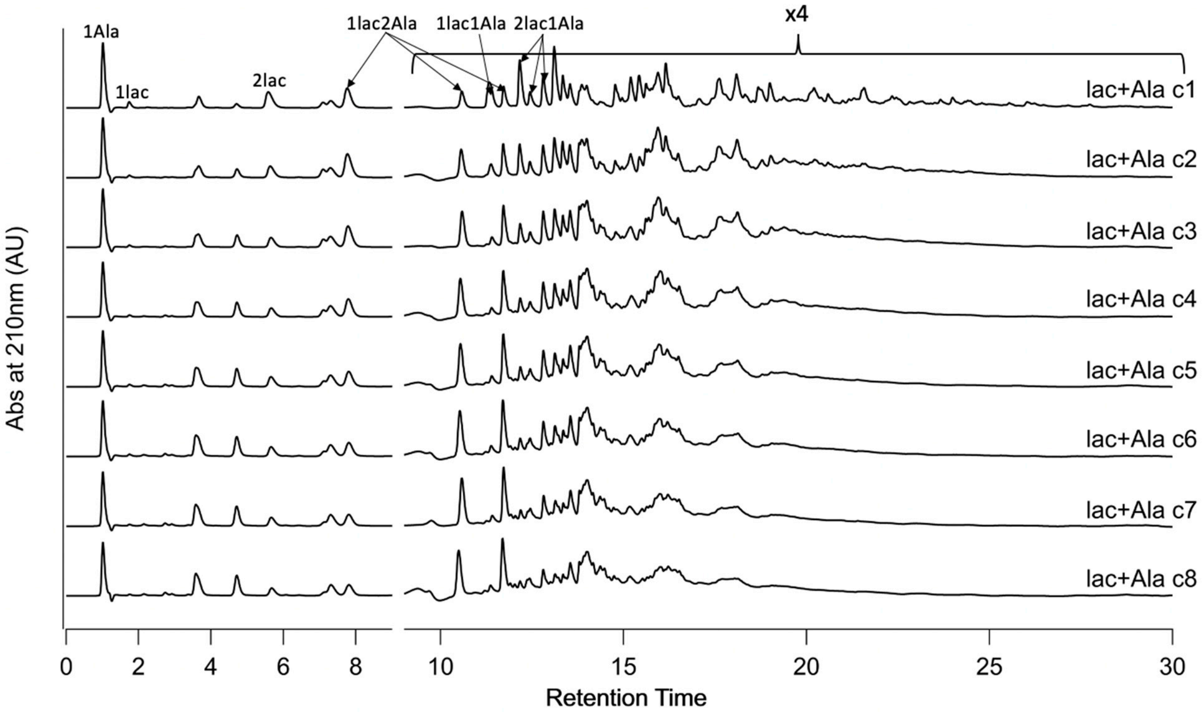

Figure S80. C18-HPLC analysis following weekly dry/wet cycling of lactic acid (lac) and Alanine (Ala) verifies the formation of depsipeptides. A mixture of **lac** and **Ala** was subjected to weekly dry/wet cycles (4 days dry/3 days wet) at 85 ˚C for 8 weeks with an aliquot taken at the end of each dry-state for analysis. The resulting product mixture was analyzed by hydrophobicity-based separation using C18-HPLC and the peaks were identified by LC-MS.

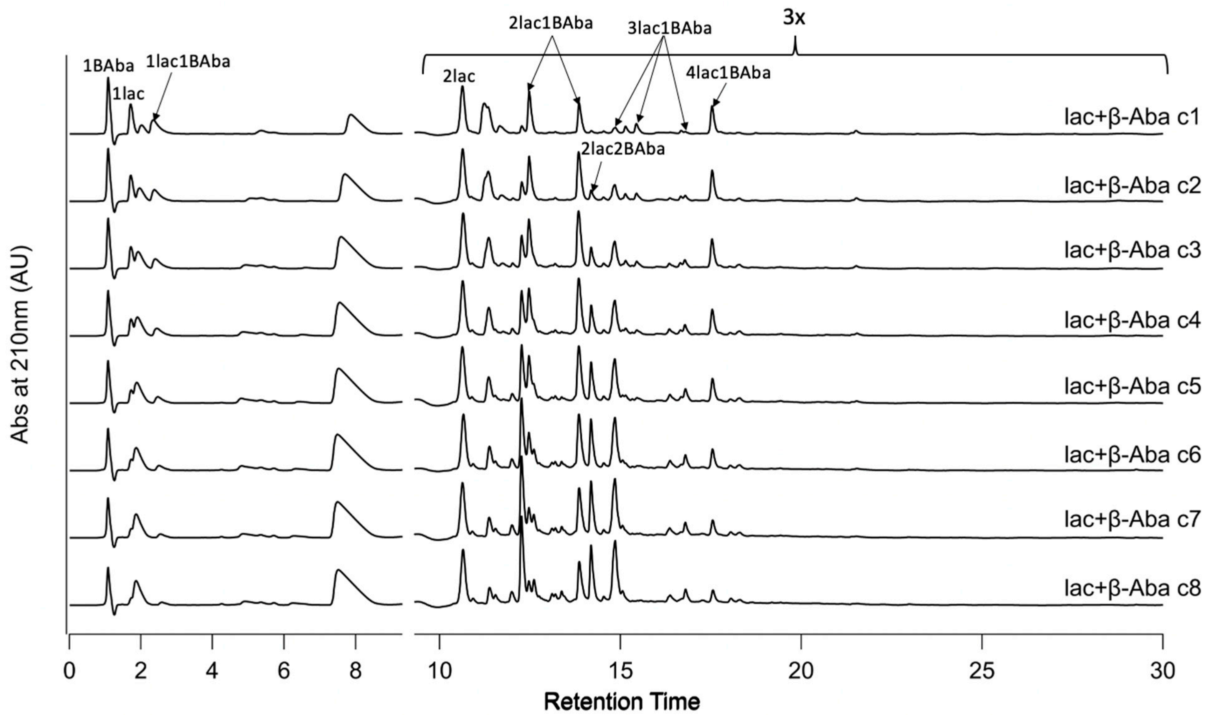

Figure S81. C18-HPLC analysis following weekly dry/wet cycling of lactic acid (lac) and β-aminobutyric acid (β-Aba) verifies the formation of depsipeptides. A mixture of **lac** and **β-Aba** was subjected to weekly dry/wet cycles (4 days dry/3 days wet) at 85 ˚C for 8 weeks with an aliquot taken at the end of each dry-state for analysis. The resulting product mixture was analyzed by hydrophobicity-based separation using C18-HPLC and the peaks were identified by LC-MS.

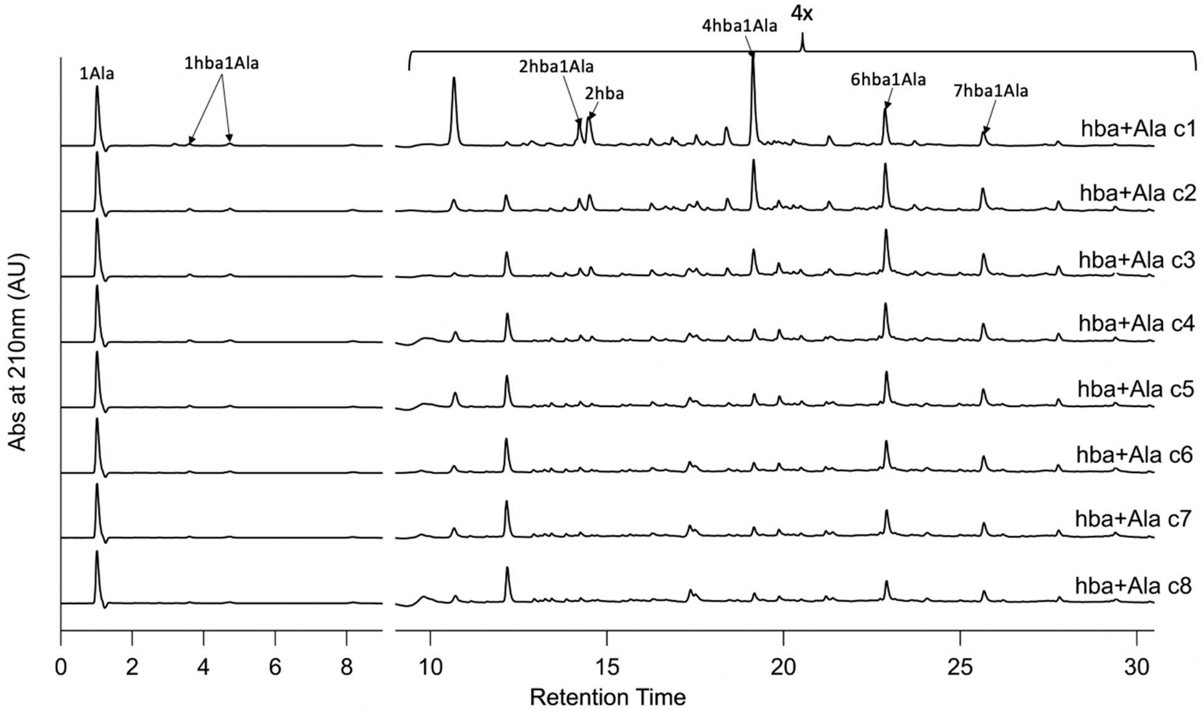

Figure S82. C18-HPLC analysis following weekly dry/wet cycling of 3-hydroxybutanoic acid (hba) and alanine (Ala) verifies the formation of depsipeptides. A mixture of **hba** and **Ala** was subjected to weekly dry/wet cycles (4 days dry/3 days wet) at 85 ˚C for 8 weeks with an aliquot taken at the end of each dry-state for analysis. The resulting product mixture was analyzed by hydrophobicity-based separation using C18-HPLC and the peaks were identified by LC-MS.

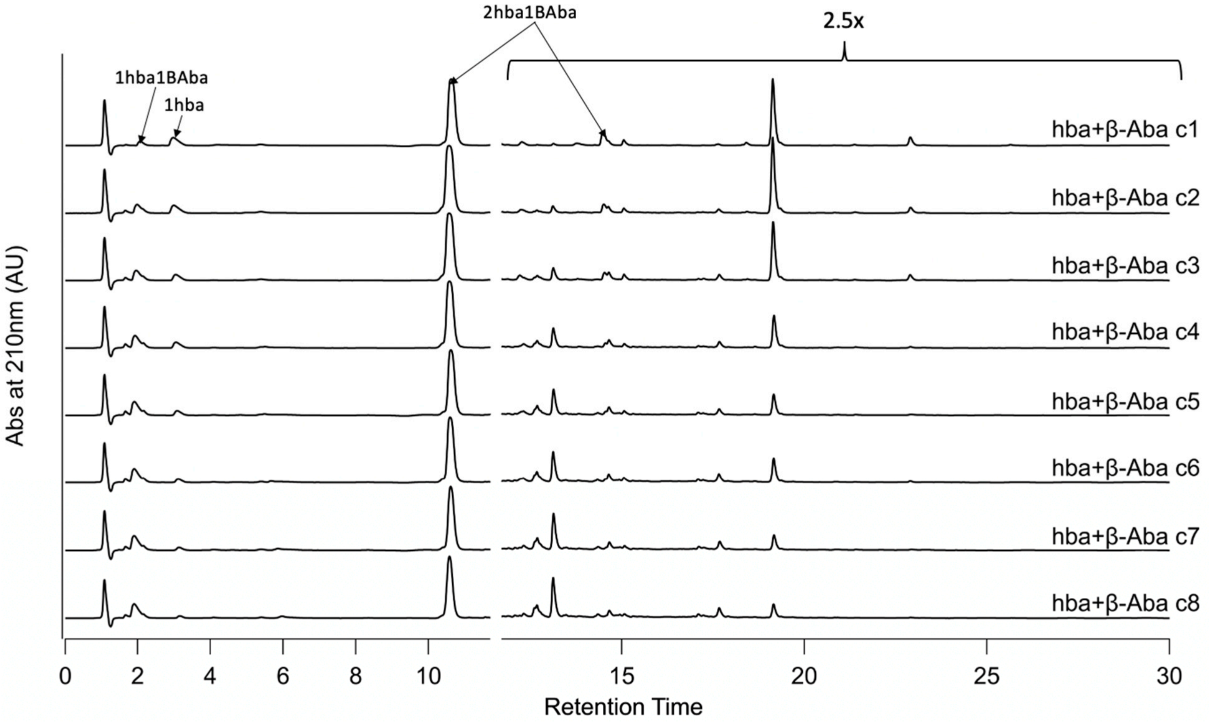

Figure S83. C18-HPLC analysis following weekly dry/wet cycling of 3-hydroxybutanoic acid (hba) and β-aminobutyric acid (β-Aba) verifies the formation of depsipeptides. A mixture of **hba** and **β-Aba** was subjected to weekly dry/wet cycles (4

days dry/3 days wet) at 85 ˚C for 8 weeks with an aliquot taken at the end of each dry-state for analysis. The resulting product mixture was analyzed by hydrophobicity-based separation using C18-HPLC and the peaks were identified by LC-MS.

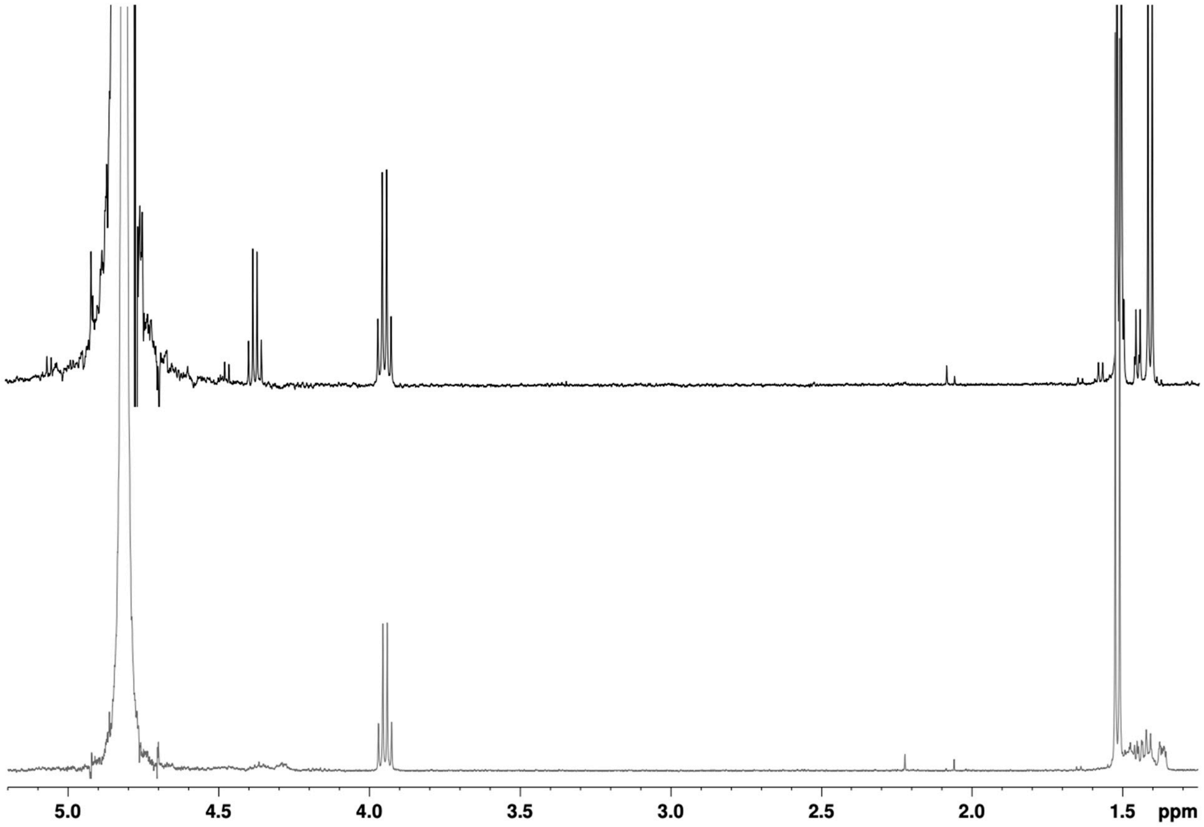

Figure S84. ^1H NMR spectra exhibit the extent of depsipeptide formation upon the final cycle of the extended dry/wet cycling of lactic acid (lac) and alanine (Ala). ^1H NMR spectra of a mixture of **lac** and **Ala** in D_2O before (black) and weekly dry/wet cycling (4 days dry/3 days wet) at 85 ˚C for 8 weeks (grey) show a 40.1% conversion of **Ala** into depsipeptide.

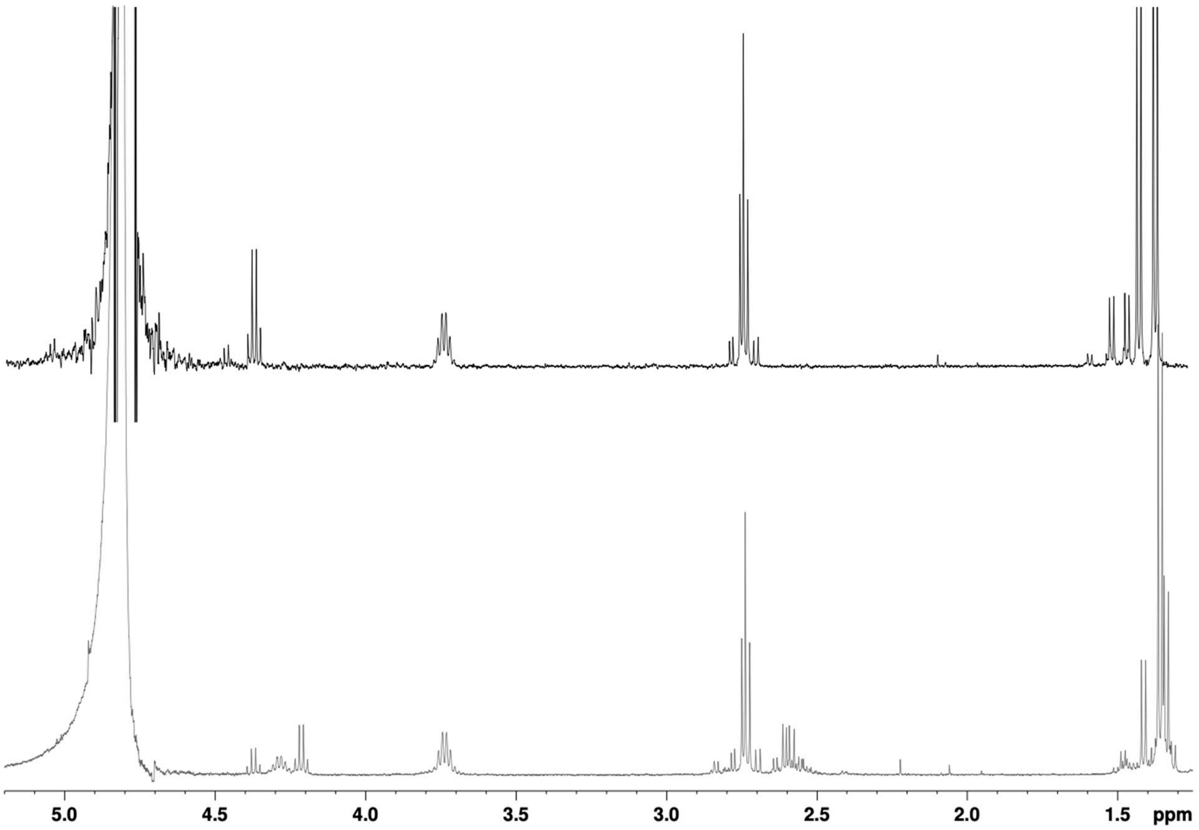

Figure S85. ^{1}H NMR spectra exhibit the extent of depsipeptide formation upon the final cycle of the extended dry/wet cycling of lactic acid (lac) and β-aminobutyric acid (β-Aba). ^{1}H NMR spectra of a mixture of **hba** and **β-Aba** in D_2O before (black) and weekly dry/wet cycling (4 days dry/3 days wet) at 85 ˚C for 8 weeks (grey) show a 47.6% conversion of **β-Aba** into depsipeptide.

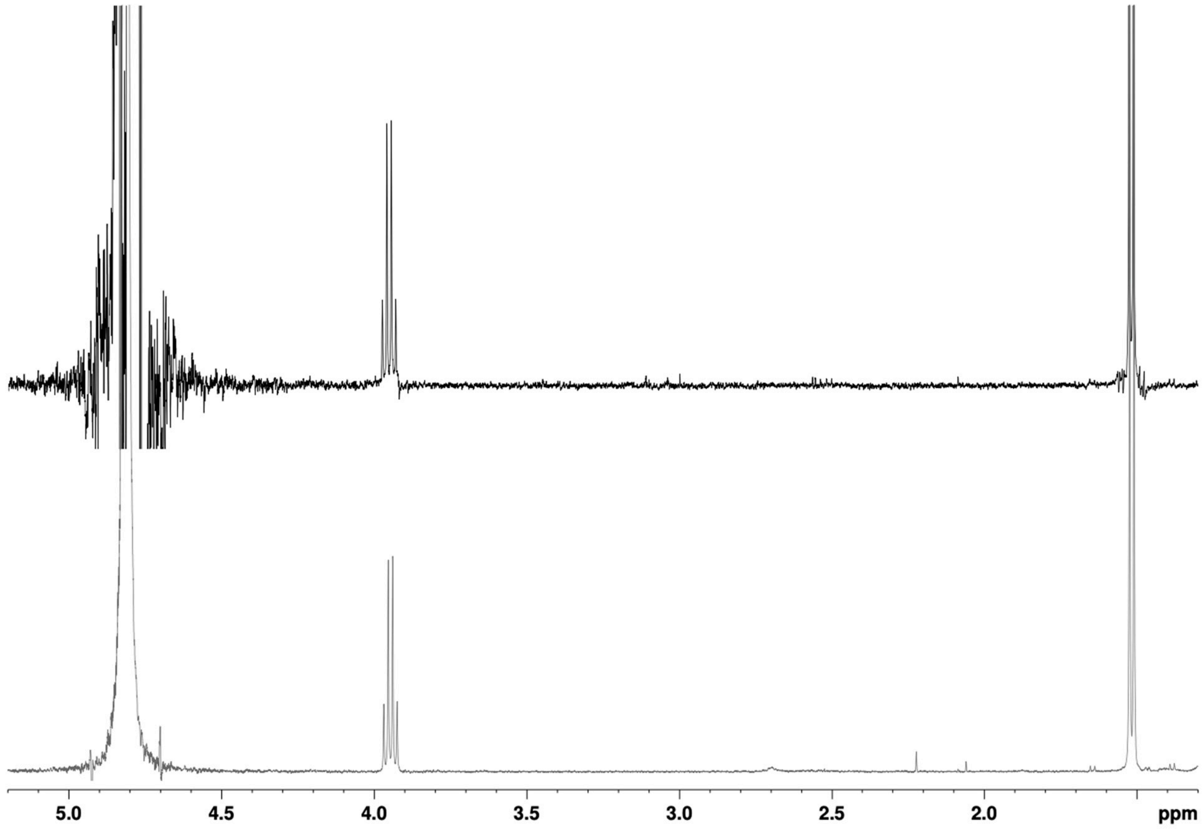

Figure S86. ^{1}H NMR spectra exhibit the extent of depsipeptide formation upon the final cycle of the extended dry/wet cycling of hydroxybutanoic acid (hba) and alanine (Ala). ^{1}H NMR spectra of a mixture of **hba** and **Ala** in D_2O before (black) and weekly dry/wet cycling (4 days dry/3 days wet) at 85 °C for 8 weeks (grey) show a 26.1% conversion of **Ala** into depsipeptide.

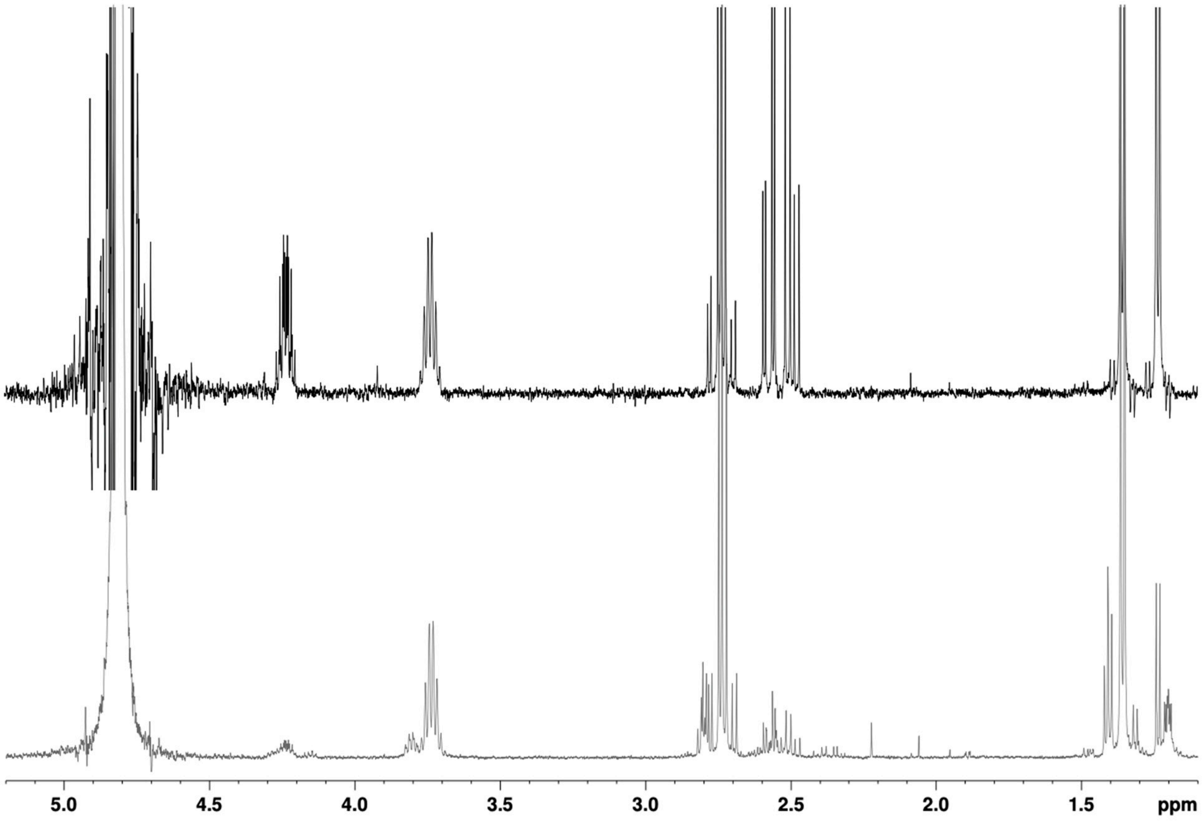

Figure S87. ^{1}H NMR spectra exhibit the extent of depsipeptide formation upon the final cycle of the extended dry/wet cycling of hydroxybutanoic acid (hba) and β-aminobutyric acid (β-Aba). ^{1}H NMR spectra of a mixture of **hba** and **β-Aba** in D_2O before (black) and weekly dry/wet cycling (4 days dry/3 days wet) at 85 ˚C for 8 weeks (grey) show a 25.8% conversion of **β-Aba** into products.
